# Redox-Active Ligand-Stabilized Lithium Iron Phosphate Nanoparticles for High-Performance Lithium-Ion Battery Cathode with High Capacities and Long-Term Stability

**DOI:** 10.1007/s40820-026-02313-6

**Published:** 2026-07-30

**Authors:** Jiwon Bok, Jeongyeon Ahn, Bogeun Park, Donghyeon Nam, Hee Seung Ryu, Uijun Lee, Jaeyeong Jang, Shihyun Chang, Sungha Choi, Minseong Kwon, Woojae Chang, Du Yeol Ryu, Daegun Kim, Hee-Dae Lim, Byung-Hyun Kim, Yongmin Ko, Jinhan Cho

**Affiliations:** 1 Department of Chemical and Biological Engineering, Korea University, Seoul, 02841 Republic of Korea; 2 Department of Applied Chemistry, Hanyang University ERICA, Ansan, 15588 Republic of Korea; 3 George W. Woodruff School of Mechanical Engineering, Georgia Institute of Technology, Atlanta, 30332 USA; 4 Department of Chemical Engineering, Hanyang University, Seoul, 04763 Republic of Korea; 5 Department of Battery Engineering, Hanyang University, Seoul, 04763 Republic of Korea; 6 KU-KIST Graduate School of Converging Science and Technology, Korea University, Seoul, 02841 Republic of Korea; 7 Department of Chemical and Biomolecular Engineering, Yonsei University, Seoul, 03722 Republic of Korea; 8 School of Chemical, Biological and Battery Engineering, Gachon University, Seongnam, 13120 Republic of Korea; 9 Division of Energy and Environmental Technology, Daegu Gyeongbuk Institute of Science and Technology (DGIST), Materials Research Institute, Daegu, 42988 Republic of Korea

**Keywords:** Rechargeable energy storage, Lithium-ion battery, High-energy lithium iron phosphate

## Abstract

Developing cathodes that simultaneously deliver high capacity, superior rate capability, and long-term cycling stability remains a major challenge in lithium-ion batteries. Here, we report a high-performance textile cathode constructed via interfacial interaction-mediated assembly of high-energy porphyrin (PP) ligand-stabilized LiFePO_4_ nanoparticles (LFP NPs). For this, 19 nm LFP NPs with olivine-type intercalation mechanism were covalently integrated with amine-functionalized PP to enable multi-electron redox activity, followed by encapsulation with multi-walled carbon nanotube (MWCNT) multilayers. Subsequent thermal annealing transformed the MWCNT layers into a covalently cross-linked conductive network. As a result, the textile cathode delivers an unprecedented specific capacity of ~260 mAh g^−1^ at ~0.1 C, excellent rate capability, and retains over 93% of its initial capacity after 2,000 cycles at 1 C with nearly 100% Coulombic efficiency. This work highlights interfacial interaction-mediated ligand assembly as a powerful strategy for next-generation high-capacity and durable cathodes.

**Supplementary Information:**

The online version contains supplementary material available at 10.1007/s40820-026-02313-6.

## Highlights

- High-energy porphyrin ligand-stabilized LiFePO_4_ (LFP) nanoparticles act as cathode components delivering high capacity and outstanding cycling stability.
- Interfacial interaction–mediated assembly effectively prevent the dissolution of porphyrin in electrolyte solution.
- The specific capacity and stability of cathodes based on high-energy LFP nanoparticles surpass those of traditional Ni-rich oxide and LFP cathodes.

**Supplementary Information:**

The online version contains supplementary material available at 10.1007/s40820-026-02313-6.

## Graphical abstract

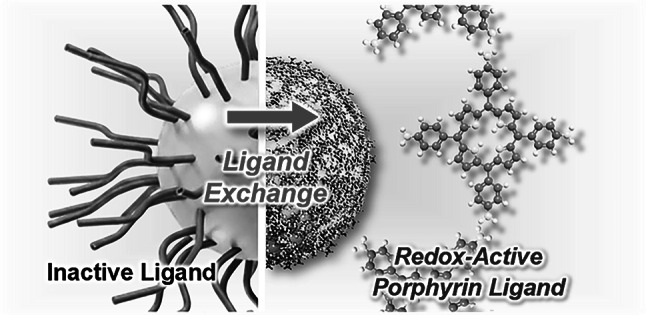

## Introduction

The rapid advancement of smart electronic devices and electric vehicles has led to high demand for high-performance lithium-ion-batteries (LIBs) that can offer a combination of high energy and power densities alongside a long lifespan. The energy density of LIBs is largely governed by the cathode—operate at relatively high voltages (3.5–4.2 V vs. Li/Li^+^) but exhibit limited specific capacity which is significantly lower than that of common anode materials such as graphite (~372 mAh g^−1^) or silicon (~3580 mAh g^−1^) [1, 2].

Widely adopted cathodes composed of inorganic transition-metal oxides such as Ni-rich layered oxides—LiNi_1−*x*−*y*_Mn_*x*_Co_*y*_O_2_ (NMC), LiNi_1−*x*−*y*_Co_*x*_Al_*y*_O_2_ (NCA), and their derivatives—and olivine-type LiFePO_4_ (LFP) represent two contrasting paradigms: Ni-rich oxides offer high capacity but suffer from limited structural and cycling stability, whereas LFP provides excellent durability and safety at the expense of restricted capacity [3–5]. As a result, most conventional cathode materials have been inherently designed to favor either capacity or stability, highlighting a fundamental materials-level dilemma in cathode engineering.

This intrinsic limitation originates from the intercalation-only mechanism that governs the traditional inorganic cathodes. Although both NMC (e.g., NMC811 with ~80% Ni) and LFP store charge via Li^+^ intercalation/deintercalation, their electrochemical behaviors diverge markedly at high states of charge. In Ni-rich NMC, deep lithiation (Li_1−*x*_MO_2_, *x* ~0.8–0.9) enables high specific capacities of 170–200 mAh g^−1^ at 1 C, but simultaneously induces pronounced anisotropic lattice distortions, phase transition, and microstructural degradation that compromise structural stability [6–8]. In contrast, LFP undergoes a two-phase intercalation mechanism within a robust olivine framework, exhibiting minimal volume change and exceptional structural integrity, albeit with low practical capacity (typically 140–165 mAh g^−1^ at 1 C) and reduced energy density [9–11]. Consequently, intercalation mechanism-based cathodes remain persistently constrained by a trade-off between attainable capacity and sustained structural stability.

Organic redox-active materials present an attractive alternative to inorganic intercalation-type cathodes, as their ability for multi-electron redox reactions enables higher theoretical gravimetric capacities [12, 13]. In principle, integrating such active organic moieties with robust inorganic intercalation hosts can provide a pathway to overcome the long-standing compromise between energy storage capability and structural/chemical stability. However, the prevailing electrode configurations—typically heterogeneous composites prepared via mechanical slurry casting—largely rely on macroscopic physical blending, resulting in weak electronic and ionic communication across component interfaces. This insufficient interfacial integration often leads to phase segregation, increased interfacial resistance, and accelerated performance degradation, thereby preventing the effective realization of synergistic functionality [14–17]. Beyond interfacial considerations, the intrinsic redox characteristics of organic materials impose an additional challenge to electrode longevity. As already mentioned above, their high theoretical energy densities are intrinsically coupled to multi-electron reaction pathways, which involve substantial molecular rearrangement and ion-pair-induced volumetric expansion during repeated cycling. These coupled chemo-mechanical stresses disrupt continuous electronic percolation networks and intensify the dissolution of redox-active species—effects that become particularly severe under bulk-loading conditions imposed by conventional slurry-based processing [12, 18].

**Scheme 1:**
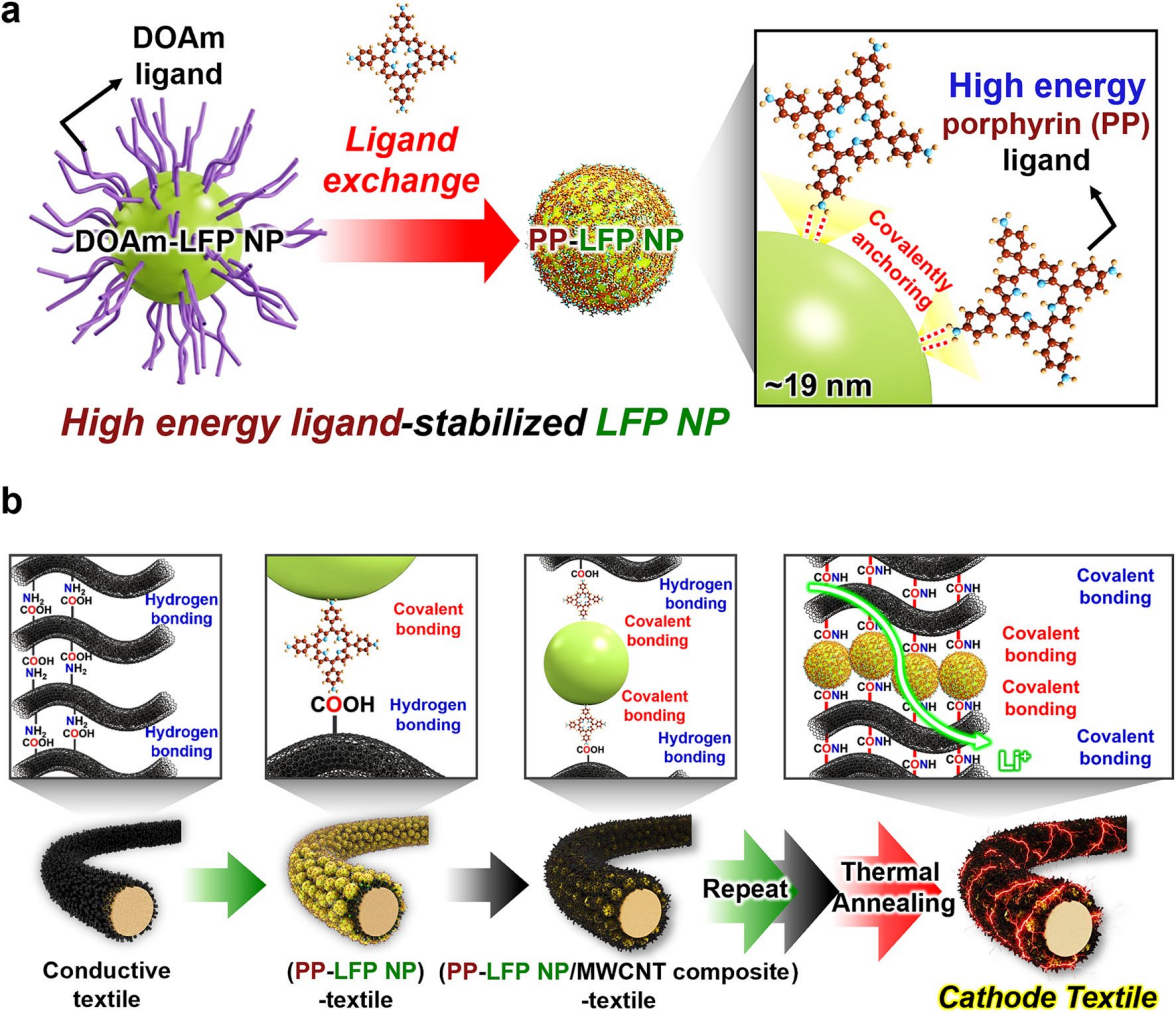
Schematic illustration. Schematic illustration of **a** PP-LFP NP prepared through the ligand-exchange reaction and **b** LbL-assembled (PP-LFP NP/MWCNT composite)_*m*_-coated textile electrode

Here, we report a textile cathode based on organic energy ligand-stabilized LFP nanoparticles (NPs), in which amine (–NH_2_)-functionalized apo-porphyrins (PPs) serve as redox-active ligands, simultaneously delivering high capacity and outstanding cycling stability (Scheme 1). In this architecture, all components are interconnected through robust covalent interactions, forming a unified cathode system (hereafter referred to as ‘PP-LFP NP’) that synergistically couples the two-phase lithiation/delithiation behavior of olivine-type LFP with the multi-electron redox activity of the organic ligands. Importantly, the PPs are directly assembled onto the NP surface via strong covalent-bonding interactions between the NH_2_ moieties of PP and the bare surface of LFP. This strong coordination effectively prevents PP dissolution in the electrolyte—a persistent limitation in conventional organic-based cathodes. The resulting precise interfacial architecture ensures the well-defined charge-storage mechanisms for the individual active components while maintaining excellent structural integrity, distinguishing it from previously reported conventional slurry-based inorganic-organic hybrid composites. Remarkably, the resulting cathode delivers ~260 mAh g^−1^ at 20 mA g^−1^ (~0.1 C), surpassing the performance of Ni-rich oxides. Even more impressively, it retains 93% of its initial capacity (~200 mAh g^−1^) after 2000 cycles at 200 mA g^−1^ and 80% of its initial capacity (~144 mAh g^−1^) after 1300 cycles at 2000 mA g^−1^—durability far beyond that of Ni-rich cathodes. Additionally, this design effectively suppresses electrolyte-induced dissolution of organic energy ligands, eliminates the need for polymeric binders, and ensures stable cycling with nearly 100% Coulombic efficiency. Furthermore, simple multi-stacking of the textile cathodes enables a proportional increase in areal capacity with stacking number, without the need for additional current collectors—an advantage that is fundamentally inaccessible to conventional nonporous planar electrodes. These results reveal the synergistic interplay between molecular-level and structural optimization, demonstrating that robust, ligand-directed interfacial interactions with inorganic active components can fundamentally enhance electrochemical performance, offering a compelling pathway toward next-generation energy storage systems.

## Experimental Section

### Materials

Cellulose textile (thickness: ~600 μm) was purchased from NamYang Nonwoven Fabric Co. Ltd. (Republic of Korea), and multiwalled carbon nanotubes (MWCNTs, diameter 7~9 nm, length 10~50 μm, purity >80%) were purchased from Nanosolution Co. Ltd. (Republic of Korea). 5,10,15,20-Tetrakis(4-aminophenyl)porphyrin (i.e., NH_2_-PP, 95% purity) was purchased from Aladdin. Oleic acid (>98% purity) was purchased from Uniam Co. Ltd. (Republic of Korea). 1-(3-Dimethylaminopropyl)-3-ethylcarbodiimide methiodide (i.e., EDC, 98% purity) was purchased from Alfa Aesar. Organic solvents (ethanol, and toluene) were obtained from Daejung Chemicals & Metals (Republic of Korea). All other chemical reagents were purchased from Sigma-Aldrich and used without further purification.

## Results and Discussion

### Adsorption Behavior of (PP-LFP NP/MWCNT composite)_***m***_ Multilayers

**Fig. 1:**
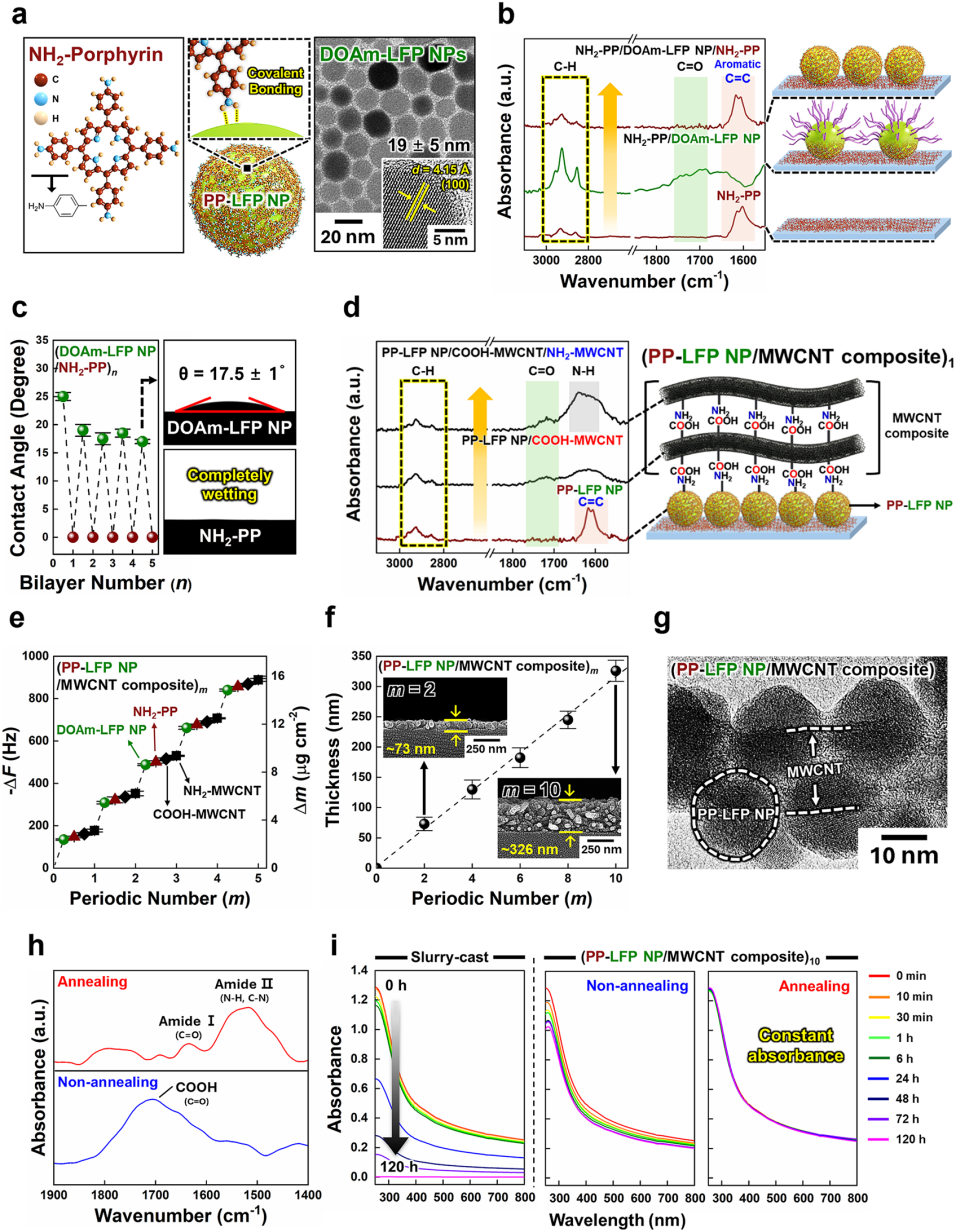
**a** Schematic illustration of the LFP NP covalently integrated with NH_2_-PP ligands (middle), along with the molecular structure of NH_2_-PP (left) and the HR-TEM image of DOAm-LFP NPs (right). The insets of the HR-TEM image indicate the lattice fringe spacing (*d*) and corresponding crystal plane of LFP NP. **b** FTIR spectra and schematic illustration of (NH_2_-PP/DOAm-LFP NP/NH_2_-PP) multilayers obtained by sequentially depositing each layer. **c** Electrolyte contact angles of the outermost layer-dependent (DOAm-LFP NP/NH_2_-PP)_*n*_ multilayers.** d** FTIR spectra and schematic illustration of (PP-LFP NP/MWCNT composite)_1_ multilayers obtained by sequentially depositing each layer.** e** QCM analysis of the (PP-LFP NP/MWCNT composite)_*m*_ multilayers as a function of periodic layer number (*m*).** f** Film thickness of the (PP-LFP NP/MWCNT composite)_*m*_ multilayers as a function of the periodic layer number (*m*).** g** HR-TEM image of the PP-LFP NP/MWCNT composite multilayers. **h** FTIR spectra of (PP-LFP NP/MWCNT composite)_10_ multilayers without (blue) and with (red) thermal annealing at 250 °C. **i** UV–Vis absorption spectra of the time-dependent dissolution behavior of PP components in the (PP-LFP NP/MWCNT composite)_10_ multilayers and the slurry-cast films immersed in an electrolyte solution

First, high-energy NH_2_-PP organics were assembled onto the as-synthesized and highly crystalline 19±5 nm-sized DOAm-LFP NPs dispersed in toluene (Figs. 1a and S1). During this NH_2_-PP assembly, the bulky and electrochemically inactive DOAm ligands initially bound to the LFP NP surface were replaced by the NH_2_ groups of NH_2_-PP through a thermodynamically favorable ligand-exchange process. This exchange occurs because the *-*NH_2_ groups exhibit a stronger binding affinity toward the exposed LFP surface than the native DOAm ligands.

As illustrated in Fig. 1a, the NH_2_-PP possesses a macrocyclic porphyrin structure containing four nitrogen heteroatoms and an 18π-electron conjugated system, as further evidenced by its characteristic UV–Vis absorption profile showing a Soret band at 425 nm and *Q* bands at 522, 564, and 656 nm (Fig. S2a). Owing to this highly conjugated electronic structure, porphyrin derivatives typically exhibit a narrow HOMO–LUMO gap (~2.552 eV), endowing them with strong electrochemical activity as redox-active electrode materials (Fig. S2b) [19]. In addition, the NH_2_ groups positioned at the *para*-sites of the meso-phenyl rings facilitate interfacial bonding with adjacent electrode components to form robust multilayered architectures and enhance interfacial wettability with the electrolyte, thereby promoting efficient ion transport. (A more detailed discussion is provided in a subsequent section.)

The progression of the ligand-exchange-driven assembly was clearly monitored by Fourier transform infrared (FTIR) spectroscopy during the stepwise LbL-assembly process. As shown in Fig. 1b, the initial deposition of LFP NPs onto the NH_2_-PP-coated substrate produced distinct vibrational bands associated with the outermost DOAm ligands (*e.g.*, C-H and C=O) (Fig. S3a) [20]. In this stage, the characteristic absorption features of NH_2_-PP substantially overlapped with those of the native DOAm ligands in the 1600 to 1200 cm^−1^ region—comprising aromatic C=C and C-N stretching as well as C-H bending modes (Fig. S3b) [21–23]. Therefore, the ligand-exchange process was evaluated by tracking distinct C=O stretching at 1749 cm^−1^ and C-H stretching bands at ~ 2925 and 2854 cm^−1^ (middle spectrum in Fig. 1b). Upon subsequent deposition of NH_2_-PP onto the LFP NP-coated substrate, the C=O and C-H stretching bands corresponding to the bulky DOAm ligands notably decreased, while absorption bands emerged at 1602 cm^−1^, which can be assigned to the aromatic C=C stretching vibrations of NH_2_-PP (top spectrum in Fig. 1b). These spectral evolutions strongly confirm the effective replacement of the native DOAm ligands with NH_2_-PP molecules during the LbL-assembly process, leading to the formation of NH_2_-PP ligand-stabilized LFP NPs (i.e., PP-LFP NPs) with a distinct interfacial architecture [24]. To further verify the effective removal of residual DOAm ligands during the ligand-exchange process, additional FTIR analysis was conducted before and after ethanol washing, as shown in Fig. S4. While the DOAm-LFP NPs exhibited characteristic C-H stretching peaks at ~ 2925 and 2854 cm^−1^, these peaks became significantly weakened after ethanol washing, indicating that the weakly adsorbed DOAm ligands were effectively removed during the ligand-exchange and washing processes. These results suggest that the remaining C-H stretching peaks in Fig. 1b primarily originate from NH_2_-PP molecules rather than residual DOAm species. Furthermore, the robust covalent anchoring of NH_2_-PP ligands onto LFP NP surface is expected to prevent the detachment or dissolution of active organic components during electrochemical operation, thereby ensuring enhanced structural integrity and long-term stability.

The successful functionalization of the NH_2_-PP on the LFP NPs was also confirmed by monitoring periodic changes in electrolyte contact angle (ECA) of LFP NP layers with different surface ligands (Fig. 1c). The DOAm-LFP NP layer exhibited a relatively high ECA of ~17.5° due to the presence of hydrophobic DOAm ligands. On the other hand, the PP-LFP NP layer displayed complete wetting behavior, indicative of significantly enhanced surface hydrophilicity. This dramatic change in wettability not only confirms the uniform and conformal coverage of the NH_2_-PP on the LFP NP surface but also highlights its ability to promote intimate electrolyte infiltration throughout the electrode structure, even within densely packed LFP NP domains.

Building on these results, conductive components consisting of carboxylic acid-functionalized multiwalled carbon nanotubes (COOH-MWCNT) and amine-functionalized carbon nanotubes (NH_2_-MWCNT) were additionally deposited on the PP-LFP NP layer via hydrogen-bonding interactions (Figs. 1d and S5). Specifically, interfacial hydrogen-bonding occurred between NH_2_-PP and COOH-MWCNT, and subsequently between COOH-MWCNT and NH_2_-MWCNT, facilitating a stepwise assembly. These hydrogen-bonding interactions were evidenced by increased FTIR absorption intensities in 1740–1700 and 1650–1550 cm^−1^ regions, corresponding to the characteristic vibrations of COOH and NH_2_ functional groups, respectively (Fig. S6) [25–28]. The resulting COOH-MWCNT/NH_2_-MWCNT multilayers (hereafter referred to ‘MWCNT composite’) form a highly porous and interconnected conductive network, which effectively reduces cell impedance and enhances charge transport kinetics by facilitating the movement of both electrons and solvated ions throughout the electrode matrix [29].

The stepwise deposition behavior of the (PP-LFP NP/MWCNT composite)_*m*_ multilayers during the LbL-assembly process was further analyzed systematically using quartz crystal microbalance (QCM) and UV–Vis absorption spectra (Figs. 1e and S7). As the number of deposition cycles (*m*) increased, the multilayers exhibited highly regular and linear growth (Fig. 1e), with an average mass increment (Δ*m*) of approximately 2.6 µg cm^−2^ per PP-LFP NP layer (consisting of 2.36 µg cm^−2^ for LFP NPs and 0.24 µg cm^−2^ for NH_2_-PP) and 0.53 µg cm^−2^ per MWCNT composite layer (comprising 0.24 µg cm^−2^ for COOH-MWCNT and 0.29 µg cm^−2^ for NH_2_-MWCNT) (see Detailed Experimental Section). Notably, in consideration of the detachment of DOAm ligands during the ligand-exchange LbL-assembly process, which resulted in a mass loss of approximately 0.28 µg cm^−2^, the recalculated mass contributions of LFP NPs and NH_2_-PP were approximately 2.08 and 0.52 µg cm^−2^, respectively. Using these corrected mass values, the mass fractions of LFP NPs, NH_2_-PP, and MWCNT composites in the electrode were estimated to be approximately 66.5%, 16.6%, and 16.9% by weight, respectively.

Field-emission scanning electron microscopy (FE-SEM) further confirmed that the total film thickness of the (PP-LFP NP/MWCNT composite)_*m*_ multilayers increased linearly from approximately ~73 to ~326 nm as *m* increased from 2 to 10, while retaining a porous and open architecture (Figs. 1f and S8). Energy-dispersive X-ray spectroscopy (EDS) elemental mapping revealed a homogeneous distribution of all constituent elements across the entire film without any signs of phase segregation or structural defects (Fig. S9). High-resolution transmission electron microscopy (HR-TEM) images further demonstrated that the PP-LFP NPs were uniformly anchored onto the MWCNT surfaces and were evenly distributed throughout the interconnected porous MWCNT network (Figs. 1g and S10).

To reinforce interfacial integrity and immobilize all components within the multilayered framework, the (PP-LFP NP/MWCNT composite)_*m*_ multilayers prepared via LbL assembly were thermally annealed at 250 °C under vacuum. Notably, this thermal annealing transformed the initially hydrogen-bonded multilayers into covalently cross-linked structures through thermally-induced amide bond formation. As shown in Fig. 1h, before annealing, a pronounced C=O stretching band associated with the -COOH groups of COOH-MWCNTs was observed in the 1740–1700 cm^−1^ range. After annealing, this peak markedly diminished, while new absorption bands appeared at 1691 and 1521 cm^−1^ corresponding to the Amide I and Amide II regions, respectively [30]. These spectral evolutions clearly verify the formation of amide bonds between COOH and NH_2_ groups, confirming the establishment of a chemically cross-linked framework that substantially enhances the interfacial robustness of the composite electrode.

The structural stability of the electrode components in electrolyte environments was further evaluated by monitoring the temporal mass variations of the (PP-LFP NP/MWCNT composite)_*m*_ multilayers upon immersion in a conventional liquid electrolyte (1 M LiPF_6_ in ethylene carbonate (EC) and dimethyl carbonate (DMC), 3:7 v/v) using UV–Vis absorption and QCM analysis (Figs. 1i and S11). As a comparison, slurry-cast mixtures composed of LFP, NH_2_-PP, MWCNTs, and poly(vinylidene fluoride) (PVDF) binder—lacking well-defined interfacial organization—exhibited a rapid decline in UV–Vis absorbance, indicating facile dissolution of NH_2_-PP components in the electrolyte. While the (PP-LFP NP/MWCNT composite)_*m*_ multilayers without thermal annealing underwent moderate degradation, the thermally annealed multilayers maintained nearly constant UV–Vis absorbance over the entire immersion period (0–120 h). Additionally, these phenomena could be quantitatively observed by QCM analysis. That is, the slurry-cast mixtures experienced a 31% mass loss after 24 h immersion, whereas the thermally annealed (PP-LFP NP/MWCNT composite)_*m*_ multilayers retained their full loading mass (Fig.S11). These results clearly demonstrate the superior chemical and structural robustness of the well-engineered multilayered architecture.

### Preparation of (PP-LFP NP/MWCNT composite)_***m***_-Assembled Textile Electrodes

**Fig. 2:**
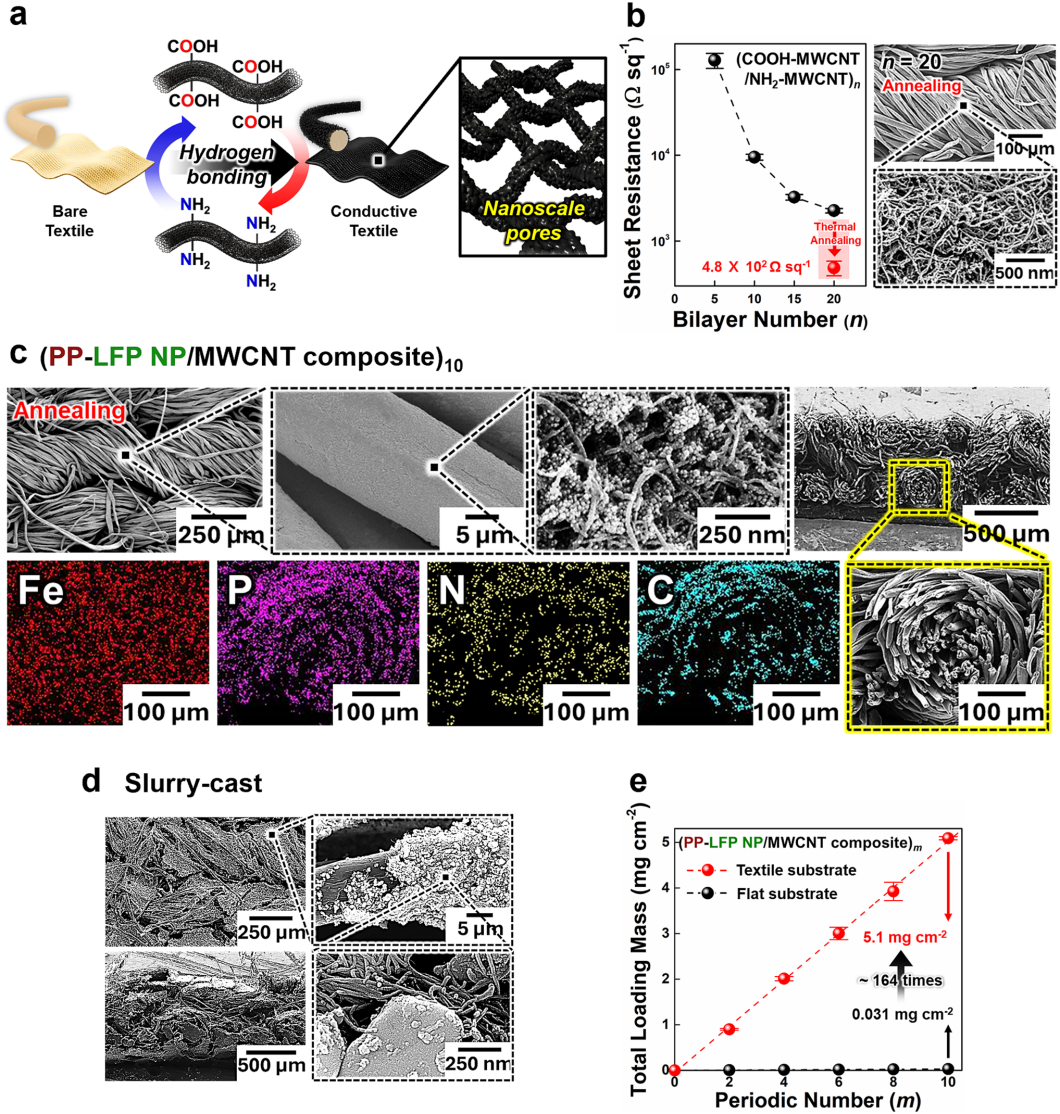
**a** Schematic illustration showing the fabrication of the conductive textile current collector via hydrogen-bonding interaction-mediated LbL assembly. **b** Sheet resistance of the (COOH-MWCNT/NH_2_-MWCNT)_*n*_-coated textiles as a function of the bilayer number (*n*) (left) and FE-SEM images of the (COOH-MWCNT/NH_2_-MWCNT)_20_-coated textiles (right).** c** Planar and cross-sectional FE-SEM images of (PP-LFP NP/MWCNT composite)_10_-coated textile electrodes. The bottom panels show the EDS elemental mapping of cross-sectional FE-SEM image. **d** Planar and cross-sectional FE-SEM images of slurry-cast textile electrodes. **e** Comparison of the total loading mass change of the (PP-LFP NP/MWCNT composite)_*m*_ multilayers deposited on the porous textile and the nonporous flat substrates as a function of periodic layer number (*m*)

To construct the (PP-LFP NP/MWCNT composite)_*m*_-assembled textile electrodes, a conductive textile substrate was preferentially prepared to function as the current collector. This was achieved by sequentially depositing COOH-MWCNTs and NH_2_-MWCNTs onto a cotton textile via LbL assembly, driven by hydrogen-bonding interactions (Fig. 2a). As the number of bilayers (*n*) in the (COOH-MWCNT/NH_2_-MWCNT)_*n*_ assembly increased up to 20, the sheet resistance of the resulting conductive textile decreased significantly, reaching approximately 2.2 × 10^3^ Ω sq^−1^ (Fig. 2b). Despite the repeated MWCNT deposition, the intrinsic fibrillar morphology of the pristine textile was well preserved, as confirmed by FE-SEM images. In addition, the MWCNT composite layers formed a conductive network enriched with abundant nanoscale pores, providing a large accessible surface area for active-material loading and thereby enabling enhanced areal capacity.

After establishing the conductive textile current collector, the (PP-LFP NP/MWCNT composite)_*m*_ multilayers were deposited onto its surface via LbL assembly. The resulting electrode was then thermally annealed at 250 °C under vacuum to improve its mechanical robustness and electrochemical stability. Following annealing, the sheet resistance of the conductive textile further decreased to ~4.8 × 10^2^ Ω sq^−1^, confirming the improved electrical connectivity within the composite framework.

A particularly distinctive feature of our approach is the uniform and conformal deposition of the LbL-assembled electrode components onto each individual fibril of the conductive textile substrate, achieved without clogging its intrinsic porous structure (Fig. 2c). Notably, the (PP-LFP/MWCNT composite)_*m*_ multilayers coated on the textile current collector exhibited nearly identical film thickness observed on the 2D substrate (Fig. S12), indicating that the interfacial interaction-mediated LbL-assembly process enables geometry-independent robust and conformal thin-film growth. As a result, the textile’s porous network was well preserved, even after thermal annealing (Fig. S13). In particular, given that the dipping process-based LbL assembly can be applied to various substrates regardless of their size and shape, our textile electrode is scalable to larger sizes while maintaining high uniformity (Fig. S14).

In sharp contrast, the conventional slurry-cast mixtures failed to produce homogeneous coatings onto the textile substrates, leading to severe blockage of the porous fibril networks and phase segregation between active particles and MWCNTs (Fig. 2d). These deficiencies mainly arise from the high viscosity of the slurry and the absence of strong interfacial interactions between the current collector and electrode materials. Consequently, slurry-based 3D porous electrodes cannot fully leverage the architectural advantages of the textile framework, resulting in nonuniform electron/ion transport pathways and weak interfacial adhesion—factors that ultimately lead to sluggish charge-transfer kinetics and poor electrochemical stability.

Importantly, our hierarchically organized yet highly porous electrode architecture facilitates efficient electron and ion transport throughout the textile framework, while simultaneously accommodating a substantially higher active-material loading per unit area compared to conventional nonporous 2D current collectors (Fig. 2e). For instance, the total loading mass of the (PP-LFP NP/MWCNT composite)_10_ multilayers on the textile substrate is approximately ~164 times greater than that achieved on a flat substrate. Moreover, the chemical stability of the textile electrode in electrolyte environments was validated by visual inspection of the immersed electrolyte and UV–Vis spectroscopy monitoring over time (Fig. S15). These results underscore the durability and robustness of the interfacial interaction-assembled textile electrode, confirming its strong potential for stable operation under practical electrochemical conditions.

### Electrochemical Properties of (PP-LFP NP/MWCNT composite)_***m***_***-***Textile Electrodes

Based on these findings, the electrochemical performance of the (PP-LFP NP/MWCNT composite)_*m*_-coated textile electrodes was systematically evaluated using cyclic voltammetry (CV), galvanostatic charge–discharge (GCD) measurements, and electrochemical impedance spectroscopy (EIS). As shown in Fig. S16, the representative CV curve of the (PP-LFP NP/MWCNT composite)_6_-coated textile electrode—with a total loading mass of approximately 3 mg cm^−2^ (mass ratio of PP-LFP NP to MWCNT composite is 4.9:1)—was recorded at a scan rate of 0.05 mV s^−1^ over a potential window of 1.4–4.3 V (vs. Li/Li^+^) (Fig. S17). As shown in Fig. S18, this voltage window was selected based on electrochemical analysis to sufficiently investigate the reversible behavior of the composite electrode while minimizing severe electrolyte decomposition. During prolonged cycling, no noticeable tailing behavior associated with parasitic electrolyte oxidation was observed in the d*Q*/d*V* profiles [31]. In addition, differential electrochemical mass spectroscopy (DEMS) analysis revealed negligible gas evolution during cycling up to 4.3 V, while noticeable gas generation was observed only beyond the applied voltage range [32, 33]. These results indicate that the selected voltage window is appropriate for investigating the intrinsic electrochemical behavior of the composite electrode. The CV curve exhibited a pair of well-defined redox peaks centered at approximately 3.40 V (cathodic) and 3.55 V (anodic), corresponding to the reversible Fe^2+^/Fe^3+^ redox couple of LFP, which reflects its characteristic two-phase lithiation/delithiation process (LiFePO_4_ ↔ FePO_4_) [34]. The redox reactions of PP molecules typically involve multiple electron transfers over broad potential ranges, exhibiting pseudocapacitive or surface-limited redox kinetics [35, 36]. As a result, each individual redox step produces relatively low-intensity CV signals compared to the strong sharp peaks of LFP NPs (Fig. S19).

**Fig. 3:**
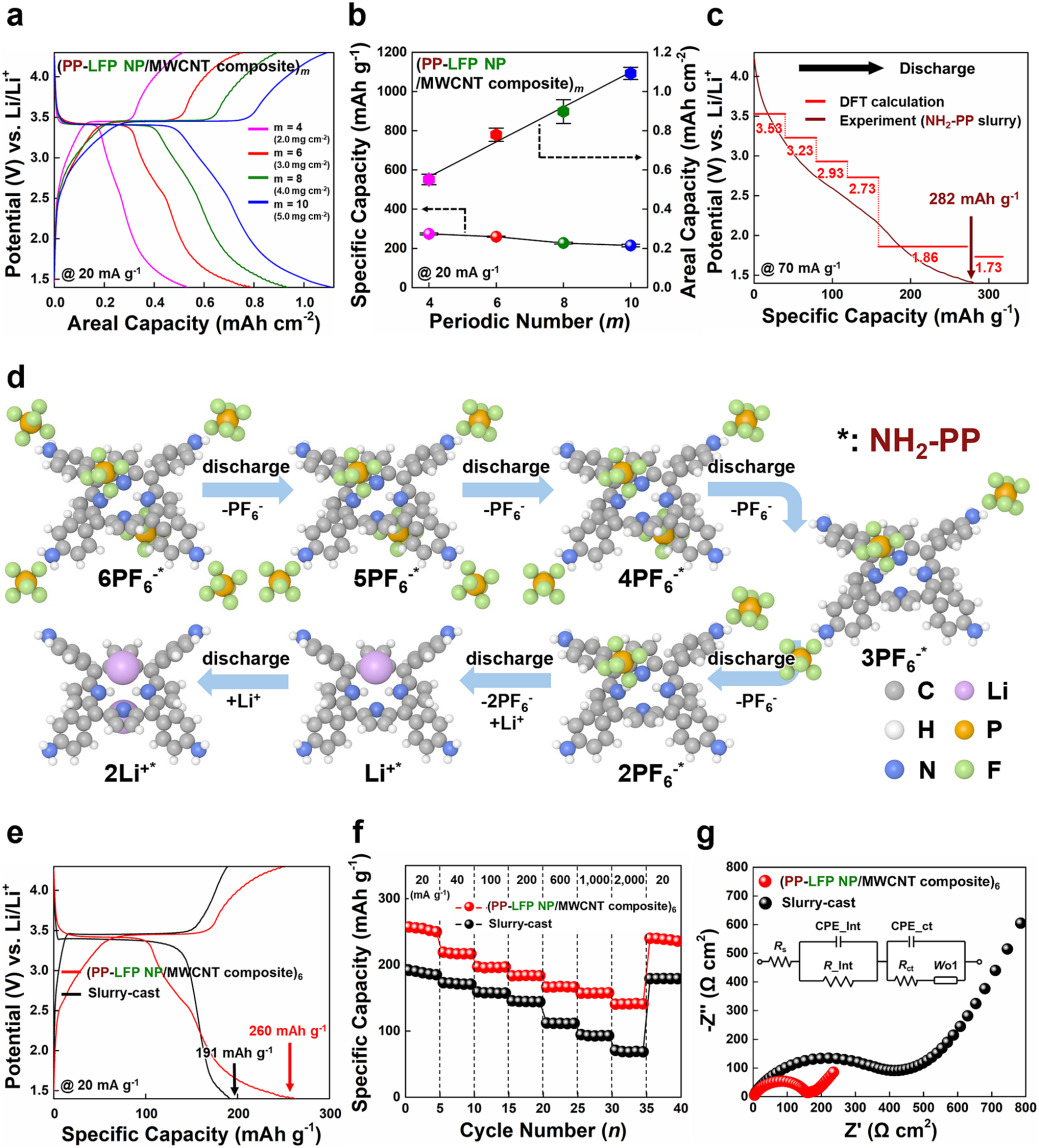
**a** GCD profiles of the (PP-LFP NP/MWCNT composite)_*m*_-coated textile electrodes with increasing periodic layer number (*m*) of 4, 6, 8, and 10 performed at a current density of 20 mA g^−1^.** b** Specific and areal capacities of the (PP-LFP NP/MWCNT composite)_*m*_-coated textile electrodes with increasing periodic layer number (*m*) of 4, 6, 8, and 10 performed at a current density of 20 mA g^−1^. **c** DFT-calculated equilibrium-voltage profiles compared with the experimentally measured GCD profile of the NH_2_-PP slurry-cast electrode performed at a current density of 70 mA g^−1^. **d** Schematic illustration of the structural evolution of NH_2_-PP during discharge, showing its reversible bipolar redox process with Li^+^ and PF_6_^−^ ions. **e** GCD profiles of the (PP-LFP NP/MWCNT composite)_6_-coated textile electrode and the slurry-cast electrode with same loading mass performed at a current density of 20 mA g^−1^.** f** Rate capability comparison between the (PP-LFP NP/MWCNT composite)_6_-coated textile electrode and the slurry-cast electrode performed over current densities from 20 to 2000 mA g^−1^. **g** Comparison of Nyquist plots for the (PP-LFP NP/MWCNT composite)_6_-coated textile electrode and the slurry-cast electrode

Figure 3a illustrates the GCD profiles of the (PP-LFP NP/MWCNT composite)_*m*_-coated textile electrodes, measured at a current density of 20 mA g^−1^, as a function of the periodic number (*m*). All electrodes displayed a distinct voltage plateau at around 3.4 V, consistent with the Fe^2+^/Fe^3+^ redox behavior observed in the CV curves. This plateau is indicative of LFP phase transformation during lithium-ion insertion and extraction, forming the Li_*x*_FePO_4_ phase (*x* = 0–1). Importantly, the areal capacities increased almost proportionally with increasing the periodic number (or total loading mass), while the overpotential remained nearly unchanged, reaching ~1.1 mAh cm^−2^ at *m* = 10 (total loading mass: ~5 mg cm^−2^). This behavior suggests a highly uniform compositional distribution and strong interfacial interactions between neighboring components within the multilayer electrode. Consequently, as the periodic number (*m*) increased from 4 to 10, the areal capacity increased substantially from ~0.55 to ~1.1 mAh cm^−2^ with only a slight decrease in specific capacity (Fig. 3b). Notably, the specific capacity (~260 mAh g^−1^ at *m* = 6) of PP-containing electrode significantly exceeded those of the PP-free (LFP NP/MWCNT composite)_6_ electrode (~194 mAh g^−1^ at 20 mA g^−1^) and the theoretical capacity of pristine LFP (~170 mAh g^−1^) (Fig. S20). The slightly higher capacity of the PP-free electrode can be attributed to the minor charge storage originating from the carboxyl groups on MWCNTs [37]. In contrast, the pronounced capacity enhancement in the PP-containing electrodes clearly demonstrates substantial additional charge storage provided by the redox-active PP ligands, highlighting their pivotal contribution to the Li^+^ storage. (A more detailed discussion regarding the redox mechanism and electrochemical contribution of the NH_2_-PP ligands is provided in the following sections.)

Furthermore, based on the mass fractions determined through QCM analysis, which accounts for the removal of DOAm ligands during the ligand-exchange LbL process (Fig. 1e), the theoretical capacity contributions of pure LFP, NH_2_-PP, and MWCNT composites within the (PP-LFP NP/MWCNT composite)_6_ textile electrode were estimated. This estimation utilized the specific capacities of pure LFP, NH_2_-PP, and MWCNT composites, which were prepared via the slurry casting method and evaluated at a current density of 100 mA g^−1^ (Fig. S21). The resulting estimated capacity contributions from LFP NPs, NH_2_-PP, and MWCNT composites were approximately 61.6%, 27.7%, and 10.6%, respectively. The theoretically obtainable capacity exhibited slight discrepancies from the measured value for the (PP-LFP NP/MWCNT composite)_6_-coated textile electrode, which can be attributed to variations in electrode configuration.

To clarify the electrochemical redox behavior of the (PP-LFP NP/MWCNT composite)_6_-coated textile electrode, the CV measurements were conducted at various scan rates, as illustrated in Fig. S22b. The electrode demonstrated broad but reversible redox features with increasing scan rates. To elucidate the origin of the enhanced capacity, which exceeds the theoretical capacity of LFP, additional electrochemical kinetic analyses were performed for the (PP-LFP NP/MWCNT composite)_6_ textile electrode, including *b*-value determination and analysis of capacitive versus diffusion-controlled contributions (refer to Fig. S22c–e), with reference to the NH_2_-PP slurry redox peaks displayed in Fig. S22a (see Detailed Experimental Section).

The calculated *b*-values for Peaks 1–6 were found to be 0.91, 0.87, 0.38, 0.72, 0.90, and 0.32, respectively. These values indicate the coexistence of diffusion-controlled Li⁺ intercalation (with *b* ~ 0.5) and NH_2_-PP-associated surface-controlled pseudocapacitive reactions (with *b* ~ 1) within the LbL-assembled architecture (see Fig. S22). Furthermore, the capacitive-controlled contribution progressively increased from 58.2% at 0.05 mV s^−1^ to 72.0% at 0.5 mV s^−1^, further supporting the substantial pseudocapacitive charge-storage behavior derived from the NH_2_-PP-containing interfacial structure (refer to Fig. S22d). These charge-storage mechanisms are believed to contribute significantly to the enhanced specific capacity, surpassing the theoretical capacity of pristine LFP.

To elucidate the redox mechanism of NH_2_-PP, additional analyses were conducted (Fig. S23). First, ex situ N 1*s* X-ray photoelectron spectroscopy (XPS) analysis was conducted at both the discharged (1.4 V) and charged (4.3 V) states after the 50th cycle at 200 mA g^−1^, as illustrated in Fig. S23a. In the discharged state, an additional N-Li related peak was observed at 398.1 eV, while upon charging, the N-Li feature disappeared, and a new N-PF_6_^−^ related peak emerged at 401.5 eV [38]. The reversible evolution of these features provides direct spectroscopic evidence for the proposed dual-ion redox behavior involving interactions between both Li⁺ and PF_6_^−^at the nitrogen sites of the porphyrin framework.

Additionally, d*Q*/d*V* analysis confirmed that NH_2_-PP continuously contributes to capacity evolution over a broad voltage range of 1.4–4.3 V during cycling at a current density of 70 mA g^−1^ (Fig. S23b). Furthermore, FTIR spectra were obtained before and after 300 GCD cycles to directly monitor the electrochemical participation of NH_2_-PP within the (PP-LFP NP/MWCNT composite)_6_ electrode (Fig. S23c). In this context, the emergence of the absorption band around 855 cm^−1^ following cycling can be attributed to the vibrational mode of PF_6_^−^ species, thereby confirming the incorporation of anions into the porphyrin-containing electrode during electrochemical operation. This observation provides strong evidence that the porphyrin moieties participate in p-type redox reactions, accompanied by the insertion and de-insertion of PF_6_^−^ to maintain charge neutrality throughout the oxidation and reduction processes [39, 40]. Consequently, we propose that the redox evolution of NH_2_-PP arises from a dual-ion redox mechanism that involves the participation of both Li^+^ and PF_6_^−^ ions.

To further confirm that NH_2_-PP functions as an electrochemically active-redox component within the LbL-assembled textile electrodes, its intrinsic redox behavior was systematically investigated through a combination of density functional theory (DFT) calculations and experimental analyses. As shown in Fig. S24a, NH_2_-PP delivers a high specific capacity of ~282 mAh g^−1^ performed at 70 mA g^−1^. A comparison between the theoretical capacity analysis and electrostatic potential (ESP) mapping reveals that this capacity corresponds to an eight-electron transfer process involving a reversible dual-ion insertion/extraction mechanism with two Li^+^ and six PF_6_^−^ ions, which is consistent with the full utilization of the nitrogen-based active sites (Fig. S24b–d). In this context, eight electrons per porphyrin are engaged in the redox process. Based on the DFT calculation, the overall dual-ion redox reaction can be described as follows:

1 $$\left[ {{\mathrm{PP}}^{6 + } \left( {{\mathrm{PF}}_{6}^{ - } } \right)_{6}} \right] + 2{\mathrm{Li}}^{ + } + 8e^{ - } \rightleftharpoons \left[ {\left( {{\mathrm{Li}}^{ + } } \right)_{2} {\mathrm{PP}}^{2 - } } \right] + 6{\mathrm{PF}}_{6}^{ - },$$

where PP denotes the NH_2_-PP framework. Additionally, convex-hull analysis indicates that the pristine and one PF_6_^−^-bound states are thermodynamically unstable (Fig. S25). Accordingly, the discharge is predicted to proceed via a direct transition from the two-PF_6_^−^-bound state to the one-Li^+^-bound state, bypassing these unstable intermediates. This energetically favorable pathway enables accurate calculation of equilibrium potentials, yielding a theoretical discharge profile that closely matches the experimentally measured GCD curve (Fig. 3c). The corresponding structural evolution during the redox process is schematically illustrated in Fig. 3d. These results indicate that the dual-ion redox behavior of NH_2_-PP enables it to function as an effective and integral charge-storage component within the (PP-LFP NP/MWCNT composite)_*m*_-coated textile electrodes. Therefore, the overall charge/discharge reaction of the PP-LFP NP system can be expressed as follows:

2 $$m{\mathrm{FePO}}_{4} + \left[ {{\mathrm{PP}}^{6 + } \left( {{\mathrm{PF}}_{6}^{ - } } \right)_{6} } \right] + \left( {m + 2} \right){\mathrm{Li}}^{ + } + \left( {m + 8} \right)e^{-} \rightleftharpoons m{\mathrm{LiFePO}}_{4} + \left[ {\left( {{\mathrm{Li}}^{ + } } \right)_{2} {\mathrm{PP}}^{2 - } } \right] + 6{\mathrm{PF}}_{6}^{ - }$$

This equation reflects the combined charge-storage reaction involving both Li^+^ intercalation into LFP and the dual-ion redox reaction of NH_2_-PP.

For comparison, a slurry-cast 2D Al foil-based electrode was prepared by blending LFP, NH_2_-PP, MWCNTs, and PVDF using the same composition ratio as the (PP-LFP NP/MWCNT composite)_*m*_ multilayers. As shown in Figs. 3e and S26a, the slurry-cast electrode (3 mg cm^−2^ active-material loading) delivered significantly lower specific and areal capacities (~191 mAh g^−1^ and 0.57 mAh cm^−2^) compared to those of (PP-LFP NP/MWCNT composite)_6_-coated textile electrode (~260 mAh g^−1^ and 0.78 mAh cm^−2^) at 20 mA g^−1^. The superior rate capability of the LbL-assembled textile electrode is further demonstrated in Figs. 3f and S26b, c. Even at a high current density of 2000 mA g^−1^ (10 C), the textile electrode delivered 140 mAh g^−1^, whereas the slurry-cast one exhibited only 70 mAh g^−1^. Upon returning to 20 mA g^−1^, the textile electrode recovered 94% of its original capacity, demonstrating stable reversibility and facile charge transport. Correspondingly, the textile electrode achieved specific energy and power densities of 686.5 Wh kg^−1^ and 53 W kg^−1^ at 20 mA g^−1^, and 386.4 Wh kg^−1^ and 5500 W kg^−1^ at 2000 mA g^−1^, respectively (Fig. S27 and Table S1).

Given that both electrodes share the same overall loading mass and composition ratio, the pronounced differences in electrochemical performance can be attributed primarily to their fundamentally distinct structural and interfacial characteristics. The LbL-assembled (PP-LFP NP/MWCNT composite)_6_ textile electrode offers a hierarchically porous and well-interconnected network that facilitates rapid ion diffusion, efficient electron transport, and uniform current distribution throughout the electrode. In contrast, the slurry-cast electrode suffers from tortuous ionic pathways, poor electronic percolation, and significant interfacial contact resistance, owing to its thick, inhomogeneous microstructure with poorly engineered interfaces.

These interfacial and structural disparities are clearly reflected in their electrochemical kinetics. In the CV profiles, the slurry-cast electrode displayed a considerably larger peak separation (Δ*E*_*p*_) between the cathodic and anodic peaks compared with the textile cathode (Fig. S28), indicating higher polarization and slower charge-transfer kinetics. By contrast, the LbL-assembled textile electrode displayed sharper and more intense redox peaks, signifying a more homogeneous distribution of active materials and more uniform redox reactions [41].

Further insight is provided by the Nyquist plots, which reveal marked differences in impedance characteristics between the two electrodes (Fig. 3g). As shown in Fig. S29, the slurry-cast electrode exhibited significantly higher charge-transfer resistance (*R*_ct_ ~416 Ω cm^2^) and Warburg impedance coefficient (σ_w_ ~409 Ω s^−0.5^) values compared with the (PP-LFP NP/MWCNT composite)_6_ textile electrode (approximately 160 Ω cm^2^ and 55 Ω s^−0.5^). Additionally, EIS analysis during electrochemical cycling revealed that the (PP-LFP NP/MWCNT composite)_6_ textile electrode maintained relatively stable impedance characteristics even after prolonged cycling performed at a current density of 1000 mA g^−1^, whereas the slurry-cast electrode exhibited a pronounced increase in the semicircle diameter in the Nyquist plots, indicating progressive interfacial degradation and deteriorated charge-transfer kinetics (Fig. S30). Quantitative fitting analysis further confirmed that the conformal LbL architecture and well-connected MWCNT network effectively stabilized the electrode/electrolyte interface and facilitated efficient charge-transfer behavior throughout repeated electrochemical reactions.

These results clearly highlight the superior interfacial quality and ion/electron transport efficiency of the LbL-engineered textile electrode. Overall, we believe that the enhanced electrochemical performance of the (PP-LFP NP/MWCNT composite)_6_ textile electrode arises from both the intrinsic redox contribution of NH_2_-PP and the structural integration between LFP NPs and NH_2_-PP enabled by the conformal LbL architecture.

To clearly illustrate the significance of both effects, comparative analyses were systematically conducted using the porphyrin-free (LFP NP/MWCNT composite)_6_ electrode and the slurry-cast electrode (Fig. S31). Notably, only the (PP-LFP NP/MWCNT composite)_6_ textile electrode exhibited distinct redox evolution during cycling, while the other two electrodes demonstrated significantly weaker electrochemical activity. Accordingly, these results convincingly demonstrate that both the presence of NH_2_-PP and its cooperative interaction with LFP NPs are essential for realizing the proposed charge-storage mechanism.

### Long-term Durability of the (PP-LFP NP/MWCNT Composite)_***m***_-Textile Electrodes

**Fig. 4:**
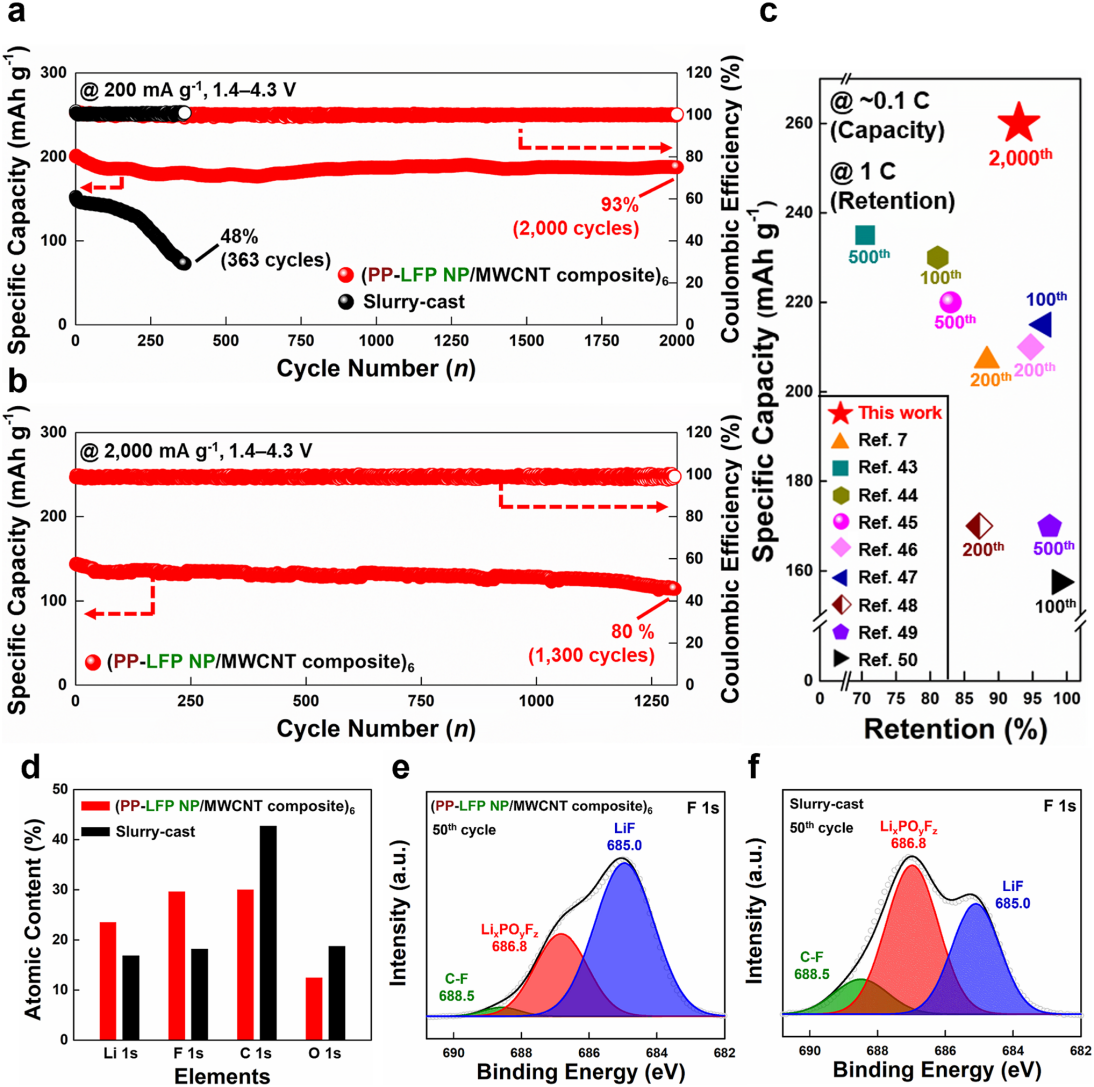
**a** Comparison of the cycling performance of the (PP-LFP NP/MWCNT composite)_6_-coated textile electrode and the slurry-cast electrode as a function of cycle number (*n*) performed at a current density of 200 mA g^−1^. **b** Cycling performance of the (PP-LFP NP/MWCNT composite)_6_-coated textile electrode as a function of cycle number (*n*) performed at a current density of 2,000 mA g^−1^. **c** Comparison of specific capacities and cycling retention of the (PP-LFP NP/MWCNT composite)_6_-coated textile electrode with previously reported cathodes based on Ni-rich oxides or LFP. **d** Elemental atomic contents (%) of the (PP-LFP NP/MWCNT composite)_6_-coated textile electrode and the slurry-cast electrode after the 50th cycle performed at a current density of 200 mA g^−1^. **e** Deconvoluted F 1*s* XPS spectra of the (PP-LFP NP/MWCNT composite)_6_-coated textile electrode after the 50th cycle performed at a current density of 200 mA g^−1^. **f** Deconvoluted F 1*s* XPS spectra of the slurry-cast electrode after the 50th cycle performed at a current density of 200 mA g^−1^

Long-term cycling performance and Coulombic efficiency were evaluated under continuous GCD cycling at a current density of 200 mA g^−1^ (Fig. 4a). The LbL-assembled textile electrode showed outstanding cycling stability, retaining approximately 93% of its initial specific capacity (~200 mAh g^−1^) even after 2000 cycles, while maintaining a nearly constant Coulombic efficiency of ~100% (Fig. S32a). In stark contrast, the slurry-cast electrode experienced a rapid capacity fading, retaining only 48% of its initial specific capacity (~152 mAh g^−1^) after 363 cycles. As shown in Fig. S32b, the GCD profiles of the LbL-assembled textile electrode recorded at the 1st, 50th, 100th, 500th, 1000th, and 2000th cycles displayed nearly identical shapes, demonstrating its excellent structural and electrochemical stability over prolonged cycling.

To investigate the origin of the cell failure after prolonged cycling performed at a current density of 200 mA g^−1^, the GCD profile at the failure cycle and post-cycling FE-SEM analyses were additionally examined, as shown in Fig. S33. The (PP-LFP NP/MWCNT composite)_6_-coated textile electrode maintained stable cycling performance up to the 2144th cycle, retaining approximately 92% of its initial capacity with a Coulombic efficiency of ~99% (Fig. S33a). However, abrupt failure occurred at the 2145th cycle, accompanied by a sudden voltage drop, indicating an internal short-circuit event rather than gradual electrochemical degradation. Post-cycling FE-SEM images revealed that the fibrillar multilayer framework of the textile electrode remained well preserved even after 2145th cycle. In contrast, the cycled Li metal anode exhibited pronounced dendritic growth compared to pristine Li metal. These results suggest that the ultimate cell failure primarily originated from dendrite-induced internal short circuiting at the Li metal anode rather than structural degradation of the multilayer cathode architecture.

Building upon its mechanically and electrochemically reinforced multilayer framework, the LbL-assembled textile electrodes also delivered excellent cycling stability under elevated current densities (Figs. 4b and S34). Even under fast charge/discharge operation, the electrode maintained ~80% of its initial capacity after 1600 cycles at 1000 mA g^−1^ and similarly retained ~80% after 1300 cycles at 2000 mA g^−1^. Such remarkable rate stabilities underscore the synergistic contributions of homogeneous ion/electron transport pathways and the covalently bonded multilayer architecture, which together ensure robust mechanical integrity and sustained electrochemical activity throughout long-term, high-rate cycling.

To investigate the role of thermal annealing on the electrochemical durability of the multilayered architecture, cycling performance was additionally compared between the annealed and non-annealed (PP-LFP NP/MWCNT composite)_6_-coated textile electrodes, as shown in Fig. S35. The annealed electrode delivered a high initial capacity of ~200 mAh g^−1^ and retained approximately 93% of its capacity after 2000 cycles. In contrast, the non-annealed electrode exhibited a lower initial capacity (~173 mAh g^−1^) and rapid capacity fading, retaining only ~62% of its initial capacity after 250 cycles. These results highlight the critical role of thermal annealing in stabilizing the multilayer electrode framework during prolonged electrochemical cycling. Specifically, thermal annealing is believed to promote the formation of a thermally induced amide network and strengthen the anchoring of NH_2_-PP within the LbL-assembled architecture, thereby reinforcing interfacial integrity and mitigating structural degradation during repeated cycling.

To evaluate the suppression of parasitic shuttle reactions originating from dissolved NH_2_-PP species, additional self-discharge analyses were conducted by monitoring the open-circuit voltage (OCV) retention behavior of the cells at different states of charge (SOC = 0% and 100%) over a 24 h resting period (Fig. S36. At SOC = 0%, the cell exhibited a stable low-voltage plateau with negligible voltage variation during the resting period, indicating minimal parasitic reactions under the fully discharged state. Likewise, at SOC = 100%, only a slight initial OCV relaxation was observed, followed by a largely stable voltage profile throughout the remaining resting period. Importantly, no progressive voltage decay characteristic of shuttle effects or continuous dissolution/redeposition processes was observed under either SOC condition. These results suggest that the covalently anchored multilayer architecture effectively suppresses the dissolution and migration of redox-active NH_2_-PP species, thereby minimizing internal parasitic reactions under open-circuit conditions.

To further investigate the immobilization stability of NH_2_-PP under realistic electrochemical conditions, additional post-cycling analyses were conducted after 300 cycles at a current density of 1000 mA g^−1^. As shown in Fig. S37, digital images of the disassembled separators after cycling revealed negligible color change for the (PP-LFP NP/MWCNT composite)_6_ textile electrodes, whereas noticeable coloration changes were observed for the slurry-cast electrodes. These observations support the conclusion that the structurally integrated electrode architecture effectively suppresses the detachment, dissolution, and migration of NH_2_-PP during long-term electrochemical cycling.

Furthermore, in-situ distribution of relaxation times (DRT) analysis revealed that the lithium metal anode assembled with the (PP-LFP NP/MWCNT composite)_6_ textile electrode maintained relatively stable interfacial resistance and lithium-ion-diffusion behavior during cycling (Fig. S38). In contrast, the lithium metal anode paired with the slurry-cast electrode exhibited pronounced increases in the *R*_SEI_, *R*_film_, and *R*_diff_ regions during cycling, indicating unstable interfacial evolution and deteriorated ion transport kinetics. We believe this behavior arises from the dissolution and migration of NH_2_-PP species in the slurry-cast electrode, which subsequently leads to the formation of resistive interfacial organic layers on the lithium metal surface during repeated electrochemical reactions [42]. Specifically, the dissolved porphyrin species are thought to migrate to the Li metal surface and form organic-rich interfacial films, thereby increasing the interfacial resistance associated with the *R*_SEI_ and *R*_film_ regions. Furthermore, the continuous accumulation of such interfacial layers is expected to hinder lithium-ion transport across the lithium metal/electrolyte interface, resulting in the pronounced increase in the *R*_diff_ region during prolonged cycling. Accordingly, these results clearly demonstrate that the structurally integrated electrode design plays a critical role in stabilizing the electrode/electrolyte interface and facilitating the electrochemical behavior of the NH_2_-PP-based system.

To the best of our knowledge, the (PP-LFP NP/MWCNT composite)_6_-coated textile electrodes deliver a significantly higher specific capacity than previously reported cathodes based on Ni-rich oxides or LFP, while maintaining comparable operational stability (Fig. 4c) [7, 43–50].

We also evaluated the electrochemical performance of the (PP-LFP NP/MWCNT composite)_6_-coated textile electrodes in a pouch-cell system to assess their practical applicability. The resulting pouch cell demonstrated stable cycling performance at a current density of 1,000 mA g^−1^, retaining 72% of its initial capacity after 500 cycles with an average Coulombic efficiency of ~100% (Fig. S39).

The enhanced capacity retention of the battery cells is closely associated with the formation of a stable cathode–electrolyte interphase (CEI). To directly visualize the CEI layer formed during the initial cycles, HR-TEM analysis was performed on the (PP-LFP NP/MWCNT composite)_6_ electrode after three cycles. As shown in Fig. S40, a conformal and ultrathin CEI layer (thickness <2.5 nm) uniformly covered the surface of PP-LFP NPs at both the discharged (1.4 V) and charged (4.3 V) states. This thin, homogeneous interfacial protective layer promotes efficient ion transfer while effectively suppressing parasitic side reactions with the electrolyte—processes that typically accelerate capacity fading during long-term cycling [51].

To achieve molecular level insight into the relationship between the electrolyte system and CEI formation in the PP-LFP NP/MWCNT composite electrode, we performed DFT adsorption energy calculations using Li^+^, PF_6_^−^, EC, and DMC as representative electrolyte-related species. The PP-LFP NP is composed of NH_2_-PP ligands that are covalently bound to LFP NPs. Therefore, the NH_2_-PP and the LFP (010) surface were considered as representative interfacial sites for comparing their relative affinities toward electrolyte-derived species.

A more negative adsorption energy indicates a more favorable interaction. The calculated adsorption energies of Li^+^, PF_6_^−^, EC, and DMC on NH_2_-PP were -0.569, 3.647, 0.775, and -0.212 eV, respectively **(**see Fig. S41a–d**)**. For the LFP (010) surface, the corresponding adsorption energies were determined to be 0.381, 2.047, -3.795, and -1.045 eV, respectively **(**see Fig. S41e–h**)**. These results indicate the presence of species-dependent interfacial affinity within the PP-LFP NP system. Notably, EC and DMC exhibited significantly higher negative adsorption energies on the LFP (010) surface compared to NH_2_-PP, suggesting that carbonate-based electrolyte solvent molecules demonstrate enhanced interactions with the LFP surface. In contrast, Li^+^ showed a more favorable adsorption energy on NH_2_-PP than on the LFP (010) surface, indicating a preferential interaction between Li^+^ and the NH_2_-PP ligand. The adsorption of PF_6_^−^ was found to be energetically unfavorable on both NH_2_-PP and LFP surfaces.

These adsorption energy trends suggest that CEI-related interfacial chemistry in the PP-LFP NP/MWCNT composite electrode is influenced not by a single component alone, but rather by species-dependent interactions with both the LFP surface and the NH_2_-PP ligand environment. Specifically, the enhanced binding of EC and DMC to the LFP (010) surface highlights a close association between the carbonate-solvent-derived interfacial reactions and the LFP/electrolyte interface. Furthermore, NH_2_-PP may enhance the local interfacial environment through favorable interactions with Li⁺. The resulting CEI layer has been shown to act as a passivating interphase, thereby suppressing continuous electrolyte decomposition and contributing to the improved cycling stability of the PP-LFP NP/MWCNT composite electrode. In addition, this interpretation was experimentally supported by the N 1*s* XPS analysis performed before cycling and after the 1st discharge process, where the appearance of an N-Li-related peak after discharge suggested the involvement of NH_2_-PP in Li^+^-associated interfacial chemistry and its contribution to CEI formation (Fig. S42).

Comprehensive interfacial analyses were conducted on both the (PP-LFP NP/MWCNT composite)_6_-coated textile electrode and the slurry-cast electrode after the 50th cycle at 200 mA g^−1^, using X-ray photoelectron spectroscopy (XPS). As shown in Figs. 4d and S43, the LbL-assembled textile electrode contained significantly higher Li 1*s* and F 1*s* signal intensities, associated with the formation of stable, inorganic-rich interphases. In contrast, the slurry-cast electrode showed elevated C 1*s* and O 1*s* contents, suggesting the accumulation of organic decomposition species derived from electrolyte degradation, which is typically associated with unstable CEI layers [52]. This distinction becomes more pronounced in the deconvoluted F 1*s* XPS spectra after the 50th cycle at 200 mA g^−1^ (Fig. 4e, f). The textile electrode displayed a dominant LiF peak at 685.0 eV and a comparatively smaller Li_*x*_PO_*y*_F_*z*_ component at 686.8 eV, consistent with the presence of a more robust and chemically resilient interphase [53]. In contrast, the slurry-cast electrode featured a much larger Li_*x*_PO_*y*_F_*z*_ component, indicative of a less stable CEI layer—correlating with the continuous capacity decay observed in slurry-cast cells [54]. Additional XPS analyses of the C 1*s* and O 1*s* regions further support these conclusions (Fig. S44) [53, 55].

Furthermore, the planar FE-SEM images of LbL-assembled textile electrodes after 50th, 1000th, and 2000th cycles (Fig. S45) confirmed that the porous fibrillar architecture remained intact and that the (PP-LFP NP/MWCNT composite) multilayers were strongly anchored to the textile fibers. These observations demonstrate the outstanding mechanical robustness and interfacial stability of the LbL-assembled electrodes. Overall, our results highlight that the molecularly engineered, covalently bonded LbL framework effectively stabilizes all critical interfaces within the cell—including the internal junctions among electrode components and the electrode–electrolyte boundary—thereby synergistically enhancing structural durability and long-term electrochemical performance.

### Electrode Stacking for Improving Areal Capacity

**Fig. 5:**
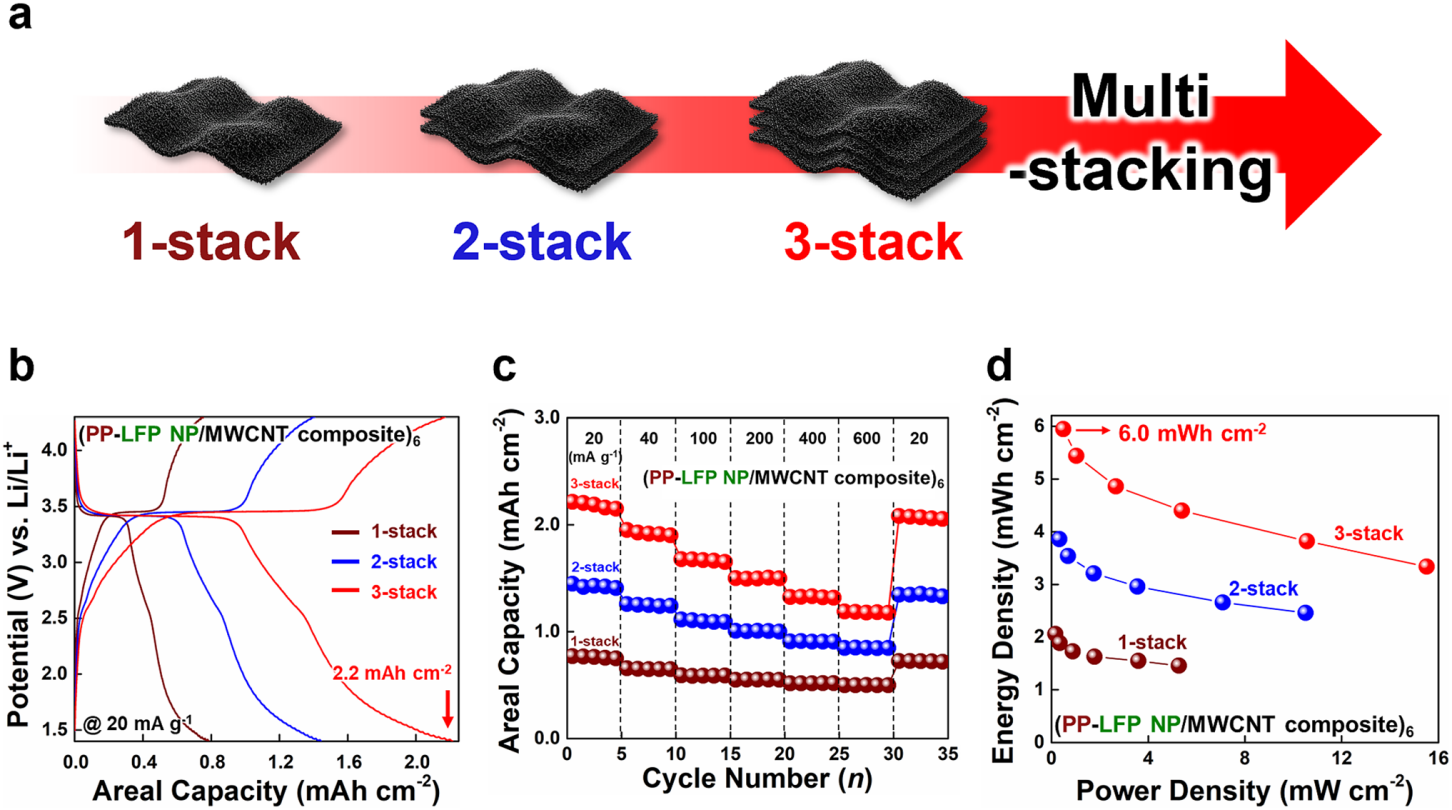
**a** Schematic illustration of the stacked cell configuration of the (PP-LFP NP/MWCNT composite)_6_-coated textile electrodes. **b** GCD profiles of the *n*-stack (PP-LFP NP/MWCNT composite)_6_-coated textile electrode as a function of stacking number (*n*) at a current density of 20 mA g^−1^. **c** Rate capability of the *n*-stack (PP-LFP NP/MWCNT composite)_6_-coated textile electrode according to the stacking number (*n*) at the current densities ranging from 20 to 600 mA g^−1^. **d** Energy and power densities of the *n*-stack (PP-LFP NP/MWCNT composite)_6_-coated electrodes as a function of stacking number (*n*)

To further enhance the areal capacity without compromising charge transport efficiency, we designed a stacked architecture of the (PP-LFP NP/MWCNT composite)_*m*_-coated textile electrode, where each electrode was capped with a well-connected MWCNT composite outermost layer that serves as a highly conductive interfacial pathway (Fig. 5a). This configuration enables seamless electron transfer across adjacent electrodes while leveraging the intrinsic porosity and mechanical flexibility of the textile framework, thereby offering a scalable and effective strategy for achieving high-energy capability. Importantly, such multi-stacking of electrodes cannot be readily implemented using conventional 2D nonporous current collectors.

Although the areal capacity of conventional electrodes employing nonporous current collectors (i.e., metal foils) can be increased by simply raising the loading amounts of active components within a single electrode, this strategy inevitably thickens the electrode layer. Such excessive thickness results in increased internal resistance, reduced energy efficiency, and an elevated risk of delamination arising from residual mechanical stresses at the active material–collector interface [56].

On the other hand, our approach based on a porous textile current collector effectively overcomes these intrinsic limitations of conventional electrodes. To verify this advantage, electrochemical impedance spectroscopy (EIS) measurements were conducted on a 3-stack cell assembled using (PP-LFP NP/MWCNT composite)_2_-coated textile electrodes, a single (PP-LFP NP/MWCNT composite)_6_-coated textile electrode, and a slurry-cast electrode, all with an identical total mass loading of 3 mg cm^−2^. Notably, the 3-stack cell exhibited *R*_ct_ and *σ*_*w*_ values (~151 Ω cm^2^ and ~90 Ω s^−0.5^) comparable to those of the single electrode with similar values (~160 Ω cm^2^ and ~55 Ω s^−0.5^). In sharp contrast, the slurry-cast electrode showed substantially higher values (~416 Ω cm^2^ and ~409 Ω s^−0.5^). These results clearly demonstrate that the electrode multi-stacking does not significantly compromise charge-transfer and ion-diffusion kinetics relative to a single thick electrode layer, while effectively avoiding the drawbacks associated with excessive electrode thickening (Fig. S46).

As shown in Fig. 5b, the areal capacity of the *n*-stack (PP-LFP NP/MWCNT composite)_6_ textile electrodes increased linearly with stacking number (*n*), increasing substantially from ~0.78 to 2.2 mAh cm^−2^ at 20 mA g^−1^ for the 3-stack configuration (total loading mass ~9.2 mg cm^−2^). Although a moderate increase in impedance was observed (Fig. S47), the 3-stack cell still exhibited superior rate capability and performance recovery, retaining 94% of its initial capacity (Figs. 5c and S48). In particular, with increasing multi-stacking from 1 to 3, the areal energy density was significantly increased from ~2.1 to ~6.0 mWh cm^−2^ (Fig. 5d). Furthermore, the consistent linear relationship in stacking behavior suggests significant potential for achieving even higher areal capacities through further increases in the stacking number.

It is worth noting that the promising electrochemical performance of the (PP-LFP NP/MWCNT composite)_6_ electrode originates from the well-defined interfacial interaction-mediated LbL assembly of all electrode components, which enables conformal and uniform thin-film deposition throughout the 3D porous textile substrate. Such hierarchical electrode architecture effectively maximizes the accessible specific surface area while simultaneously establishing continuous charge transport pathways across the entire electrode. Although the textile-supported electrode systems may exhibit relatively lower volumetric (e.g., ~20.6 mAh cm^−3^) or electrode-level specific performance (e.g., 30.7 mAh g^−1^; 82 Wh kg^−1^) owing to the intrinsic physical characteristics of the textile substrate, favorable electrochemical kinetics and charge transport characteristics remained reasonably maintained even at increased mass loading (Fig. S49). Consequently, the LbL-assembled (PP-LFP NP/MWCNT composite)_6_ electrode achieved high areal capacity despite the comparatively low active-material loading relative to conventional slurry-cast electrodes (Table S5), indicating highly efficient electrochemical utilization of the active materials within the porous textile framework.

Furthermore, to demonstrate the scalability and practical applicability of the stacking strategy, a larger-area pouch-cell electrode was additionally fabricated using a 3 × 4 cm^2^ textile electrode with a 3-stack configuration (Fig. S50). When operated at a current density of 400 mA g^−1^, the pouch cell exhibited a high initial areal capacity of approximately 13.7 mAh and maintained a capacity retention of approximately 82% after 100 cycles, while simultaneously maintaining a coulombic efficiency of ~100%.

Overall, this simple yet powerful stacking strategy—enabled by the well-defined interfacial design of the LbL-assembled textile framework—ensures structural integrity, minimizes interfacial resistance, and enables efficient charge transport. This approach represents a critical advancement toward high-energy–density, structurally adaptive cathode systems.

## Conclusions

In conclusion, our research elucidates the feasibility of achieving high-performance cathodes characterized by exceptional specific capacities, scalable areal capacities, superior rate capabilities, and long-term operational stability via the assembly of high-energy PP ligand-stabilized LFP NPs mediated by robust interfacial interactions. This approach effectively addresses the inherent trade-off between capacity and stability that typically plagues conventional cathodes by synergistically integrating the Li^+^ ion intercalation mechanism of LFP with the redox activity of PP, facilitated through a covalently bonded PP-LFP NP architecture. Moreover, the uniform, binder-free deposition of active and conductive components throughout the 3D textile framework enhances areal capacity while ensuring efficient charge transfer within the electrode. As a result, the textile cathodes delivered a high specific capacity of approximately 260 mAh g^−1^ at ~0.1 C and exhibited excellent rate capability. They also demonstrate remarkable cycling durability, retaining over 93% of their initial capacity (200 mAh g^−1^) after 2000 cycles at 1 C and 80% of initial capacity (144 mAh g^−1^) after 1300 cycles at 10 C, while maintaining nearly 100% Coulombic efficiency. Furthermore, the straightforward multi-stacking of the porous textile electrodes facilitates a proportional increase in areal capacity with increasing stacking number, achieving ~2.2 mAh cm^−2^ for three stacked textile cathodes (total loading mass of ~9.2 mg cm^−2^). Taken together, these results demonstrate the effectiveness of our approach and provide a basis for developing the next-generation energy storage systems that combine high-energy density with long-term operational reliability.

## Supplementary Information

Below is the link to the electronic supplementary material.

Supplementary file1 (PDF 7527 kb)
