# Supplementary material for: Redox-Active Ligand-Stabilized Lithium Iron Phosphate Nanoparticles for High-Performance Lithium-Ion Battery Cathode with High Capacities and Long-Term Stability
Source: Nanomicro Lett. 2026 Jul 30;19:12. doi: 10.1007/s40820-026-02313-6 (PMC13424056; doi:10.1007/s40820-026-02313-6)
Supplement: Supplementary file 1 — Supplementary file1 (PDF 7527 kb) [file 40820_2026_2313_MOESM1_ESM.pdf]

Supporting Information for

Redox-Active Ligand-Stabilized Lithium Iron Phosphate Nanoparticles for High-Performance Lithium Ion Battery Cathode with High Capacities and Long-Term Stability

Jiwon Bok[1, †], Jeongyeon Ahn[1, †], Bogeun Park[2, †], Donghyeon Nam[1, 3, †], Hee Seung Ryu[4], Uijun Lee[5], Jaeyeong Jang[6], Shihyun Chang[1], Sungha Choi[1], Minseong Kwon[1], Woojae Chang[1], Du Yeol Ryu[7], Daegun Kim[8], Hee-Dae Lim[4,5], Byung-Hyun Kim[2, *], Yongmin Ko[9, *], Jinhan Cho[1, 6, *]

[1] Korea University, Department of Chemical and Biological Engineering, Seoul 02841, Republic of Korea

[2] Hanyang University ERICA, Department of Applied Chemistry, Ansan 15588, Republic of Korea

[3] George W. Woodruff School of Mechanical Engineering, Georgia Institute of Technology, Atlanta 30332, USA

[4] Hanyang University, Department of Chemical Engineering, Seoul 04763, Republic of Korea

[5] Hanyang University, Department of Battery Engineering, Seoul 04763, Republic of Korea

[6] Korea University, KU-KIST Graduate School of Converging Science and Technology, Seoul 02841, Republic of Korea

[7] Yonsei University, Department of Chemical and Biomolecular Engineering, Seoul 03722, Republic of Korea

[8] Gachon University, School of Chemical, Biological and Battery Engineering, Seongnam

13120, Republic of Korea

[9] Daegu Gyeongbuk Institute of Science and Technology (DGIST), Division of Energy & Environmental Technology, Materials Research Institute, Daegu 42988, Republic of Korea

† These authors equally contributed to this work.

* Corresponding authors. E-mail: jinhan71@korea.ac.kr (Jinhan Cho); yongmin.ko@dgist.ac.kr (Yongmin Ko); bhkim00@hanyang.ac.kr (Byung-Hyun Kim)

S1 Detailed Experimental Section

S1.1 Synthesis of DOAm-stabilized LFP NPs

To synthesize the dioleamide (DOAm) stabilizer, oleic acid (90 mmol) and oleylamine (90 mmol) were first mixed at a 1:1 molar ratio and reacted at 70 °C for 30 min under vacuum to through a condensation reaction. Subsequently, iron(II) diacetate (4 mmol) and lithium acetate (4 mmol) were added to the reaction mixture, which was maintained at 70 °C for an additional 30 min. During this process, the solution gradually turned into a deep black color. To ensure complete replacement of the acetate ligands coordinated to the Li and Fe metal ions with oleate, the mixture was heated to 150 °C under an inert atmosphere, and then maintained at 70 °C under vacuum for a further 30 min, yielding a transparent black solution. Phosphoric acid (4 mmol) was then introduced, and the reaction was continued at 70 °C under vacuum for 30 min to remove residual moisture. The mixture was subsequently heated to 350 °C under an inert atmosphere and held at this temperature for 1 h to complete the LFP NP formation. Upon reaching the target temperature, the solution color changed to a milky, opaque white, indicating particle nucleation and growth. After cooling to room temperature, excess organic byproducts were removed by sequential washing with toluene (good solvent) and ethanol (poor solvent), followed by centrifugation. The resulting DOAm-stabilized LFP NPs were re-dispersed in toluene and used for subsequent experiments.

S1.2 Surface functionalization of MWCNTs

Carboxylic acid-functionalized MWCNTs (COOH-MWCNTs) were prepared by treating pristine MWCNTs in a mixed acid solution containing 30 mL of sulfuric acid (H_2SO_4) and 10 mL of nitric acid (HNO_3) at 70 °C for 3 h. After completion of the reaction, the mixture was cooled to room temperature and purified by repeated centrifugation and washing with deionized water until neutral pH was reached. The obtained COOH-MWCNT film was finally re-dispersed in ethanol for subsequent use. Amine-functionalized MWCNTs (NH_2-MWCNTs) were subsequently synthesized *via* an ester coupling reaction using as-prepared COOH-

MWCNTs. Specifically, 1 g of 1-(3-dimethylaminopropyl)-3-ethylcarbodiimide methiodide (EDC) was dissolved in 40 mL of deionized water and then added to 40 mL of COOH-MWCNT solution (1 mg mL^{-1}). Ethylenediamine (10 mL) was then introduced, and the reaction mixture was vigorously stirred overnight to promote amide bond formation. The resulting dispersion was centrifuged at 10,000 rpm for 3 min and vacuum-filtered three times to thoroughly remove residual reactants. The obtained NH_2-MWCNT film was finally re-dispersed in ethanol for subsequent use.

S1.3 Conductive textile current collector

Cellulose textile substrates were first cleaned by sonication in ethanol and subsequently immersed in a polyethyleneimine (PEI, M_w ~25,000) solution (2 mg mL^{-1}) for 1 hour. After drying, the resulting amine-functionalized cellulose textiles were immersed in a COOH-MWCNT dispersion (1 mg mL^{-1}) for 20 min, followed by drying to form an initial conductive layer. Subsequently, the (PEI/COOH-MWCNT)-coated textiles were alternately immersed in NH_2-MWCNT (1 mg mL^{-1}) and COOH-MWCNT (1 mg mL^{-1}) dispersions for 20 min each, with intermediate washing steps. This LbL deposition process was repeated until the desired electrical conductivity was achieved. The resulting samples were finally dried to obtain the textile-based current collectors.

S1.4 (PP-LFP NP/MWCNT composite)$_m$-coated textile electrodes

Cathode electrodes were fabricated via layer-by-layer (LbL) assembly of DOAm-stabilized LFP nanoparticles (DOAm-LFP NPs), NH_2-PP, COOH-MWCNTs, and NH_2-MWCNTs onto conductive textile current collectors using organic solvents. The conductive textile substrates were first immersed in a DOAm-LFP NP dispersion (4 mg mL^{-1} in toluene) for 20 min. After deposition, the substrates were rinsed with pure toluene to remove loosely bound particles and then fully dried. The LFP NP-coated textiles were subsequently immersed in an NH_2-PP solution (0.3 mg mL^{-1} in ethanol) for 20 min, followed by washing with pure ethanol and drying. During this step, the bulky and electrically insulating DOAm ligands on the LFP NP surface were replaced by the amine groups of NH_2-PP through a ligand-exchange reaction involving covalent interactions. This process yields high-energy ligand-stabilized LFP nanoparticles, hereafter denoted as PP-LFP NPs. Next, the (PP-LFP NP)-coated textiles were sequentially immersed in COOH-MWCNT and NH_2-MWCNT dispersions (1 mg mL^{-1} in ethanol for each) for 20 min per step, with intermediate rinsing and drying. The resulting COOH-MWCNT/NH_2-MWCNT bilayers are referred to as the MWCNT composite. These LbL assembly steps were repeated for a desired periodic number (m) to construct the targeted multilayer electrode architecture. It is important to note that this LbL assembly approach allows each component to be robustly and uniformly adsorbed onto various substrates without the need for polymeric binders. Furthermore, it effectively removes weakly or excessively adsorbed components through washing steps, yielding the ultrathin layer per adsorption step. Finally, the as-fabricated multilayer cathodes were thermally treated at 250 °C under vacuum for 3 h to convert the hydrogen-bonding interactions between the PP-LFP NP layers and the MWCNT composite into covalent amide bonds, thereby significantly enhancing the mechanical robustness and

electrochemical stability of the cathode electrodes.

S1.5 Conventional slurry-cast electrodes

Conventional slurry-cast electrodes were prepared by blending active materials—LFP powder (average particle diameter ~5 μm), NH_2-PP, MWCNTs—with a polymer binder (polyvinylidene fluoride, PVDF). The weight ratio of the active components was fixed at 6.8:0.7:1.5:1, as determined from the mass composition obtained by quartz crystal microbalance (QCM) analysis of the (PP-LFP NP/MWCNT composite)$_m$ multilayers. The components were dispersed in 1-methyl-2-pyrrolidinone (NMP) as the solvent and stirred continuously for 24 h to form a homogeneous slurry. The resulting slurry was cast onto aluminum foil (thickness: ~20 μm) current collectors using a doctor-blade technique, with the active-material loading adjusted according to the experimental conditions. The coated electrodes were then dried under vacuum at 70 °C for 12 h to completely remove the solvent.

S1.6 Characterization

High-resolution transmission electron microscopy (HR-TEM) was performed using a Tecnai 20 microscope (FEI). High-angle annular dark-field scanning transmission electron microscopy (HAADF-STEM) and corresponding energy-dispersive X-ray spectroscopy (EDS) elemental mapping were carried out using a Talos F200X instrument (Thermo Fisher Scientific). The adsorption behavior and interfacial interactions of the LbL-assembled multilayers were examined by Fourier transform infrared (FTIR) spectroscopy using a Cary 600 spectrometer (Agilent Technologies) operated in specular reflection mode with a spectral resolution of 4 cm^{-1}. Baseline correction of the FTIR spectra was conducted using OMNIC software (Thermo Fisher Scientific). X-ray diffraction (XRD) and X-ray photoelectron spectroscopy (XPS) analyses were performed using a SmartLab diffractometer (Rigaku) and a K-Alpha$^+$ system (Thermo Fisher Scientific), respectively. All XPS spectra were calibrated with reference to the C 1s peak at 284.5 eV. Field-emission scanning electron microscopy (FE-SEM) and EDS elemental mapping were conducted using an S-4800 microscope (Hitachi). Ultraviolet-visible (UV-vis) spectroscopy was employed to characterize the LbL-assembled multilayers and to monitor electrolyte-induced dissolution of the PP component, using a Lambda 35 spectrophotometer (PerkinElmer). The sheet resistance of the electrodes was measured using a four-point probe system (MCP-T610, Mitsubishi Chemical Analytech).

To measure the loading amounts of (PP-LFP NP/MWCNT composite)$_m$ multilayers per periodic layer (m) adsorbed onto a 2D current collector, quartz crystal microbalance (QCM) measurements were carried out using a QCM200 system (Stanford Research Systems). This approach was necessary due to the extremely low loading amounts per layer. The mass of each individual layer in the (PP-LFP NP/MWCNT composite)$_m$ multilayers, as well as mass loss associated with PP dissolution, was calculated from the frequency shifts using the simplified Sauerbrey equation:

$$-\Delta F\ (\mathrm{Hz}) = 56.6 \times \Delta m \tag{S1}$$

,where $-\Delta F$ and Δm represent the frequency and mass changes, respectively.

Based on the QCM measurements, the mass fractions of LFP NPs, NH_2-PP and MWCNT composites within the (PP-LFP NP/MWCNT composite)$_m$ multilayers were estimated to be approximately 66.5, 16.6, and 16.9 wt%, respectively, where the mass loss (~0.28 μg cm^{-2}) associated with the detachment of DOAm ligands from DOAm-LFP NPs during the ligand-exchange process was taken into account.

In contrast, for highly porous textiles composed of numerous fibrils with a large surface area, the adsorbed amounts per periodic layer are significantly higher compared to those on a 2D current collector [S1-S3]. Therefore, for the (PP-LFP NP/MWCNT composite)$_m$ multilayers adsorbed onto the textile current collector, we directly measured the loading amounts per periodic layer using an analytical balance. Importantly, since the LbL assembly process is governed by the same interfacial interactions regardless of substrate geometry [S4, S5], the average mass ratios and deposition behaviors of each component obtained from QCM measurement on flat substrate can be reasonably extrapolated to 3D porous textile current collector.

Based on these results, the significant increase in the overall specific capacity of the textile electrode, following the introduction of NH_2-PP ligands onto LFP NPs, is primarily attributable to the additive effect of the high-energy ligands.

S1.7 Cell assembly and electrochemical measurements

Electrochemical measurements were performed using CR2032 coin cells (MTI) connected to a WBCS3000 multichannel battery tester (WonAtech). Cell assembly was carried out in an argon-filled glovebox (MBraun; O_2 < 0.1 ppm, H_2O < 0.1 ppm) using lithium metal foil (35 μm) as the anode, the as-prepared (PP-LFP NP/MWCNT composite)$_m$-coated textile electrode (total electrode mass including the textile: ~0.045g, total thickness of textile electrode after pressing for coin cell ~380 μm), a Celgard 2500 separator, spacer, spring and casing. Each cell was filled with 100 μL of electrolyte consisting of 1 M $LiPF_6$ dissolved in an ethylene carbonate (EC)/dimethyl carbonate (DMC) mixture (3:7 vol%) containing 10 wt% fluoroethylene carbonate (FEC). Cyclic voltammetry (CV) and Galvanostatic charge–discharge (GCD) measurements were conducted over a voltage window of 1.4–4.3 V, with CV curves obtained at the 3rd cycle and GCD profiles evaluated at the 5th cycle. Cells were initially subjected to three formation cycles at a current density of 20 mA g^{-1}, followed by cycling at various current rates. Specific capacities were calculated based on the total mass loading of the (PP-LFP NP/MWCNT composite)$_m$ electrode, including the masses of the LFP NPs, NH_2-PP ligands, and the MWCNT composite. The applied current density of 20 mA g^{-1} (approximately 0.1 C for m = 6) was applied to electrodes during cell operation. Electrochemical impedance spectroscopy (EIS) was performed over a frequency range from 100 kHz to 0.1 Hz with a perturbation amplitude of 10 mV. All electrochemical measurements were carried out at 25 °C.

Distribution of Relaxation Times (DRT) analysis was conducted using DRT tools implemented in MATLAB R2025b, in accordance with established procedures for EIS deconvolution [S6]. In-situ DRT measurements were carried out at a constant current density of 20 mA g^{-1} throughout both charge and discharge processes, with impedance spectra acquired

at 6 min intervals. A dual-channel configuration enabled the simultaneous recording of cell voltage and impedance response, employing a sinusoidal current perturbation of 10 mA over a frequency range spanning 1 MHz to 0.01 Hz (SP1, ZIVE). To monitor gaseous species generated during electrochemical cycling, in-situ differential electrochemical mass spectrometry (DEMS) was employed. The gas analysis setup was constructed by interfacing a mass spectrometer (HPR-20 R&D, Hiden Analytical) with a potentio-galvanostat (VSP, Biologic). Cells were fabricated using a custom-designed coin cell configuration, in which a 1 mm aperture was introduced at the center of the cap to facilitate the release of gases evolved at the cathode. Prior to measurement, each cell was allowed to rest for 8 h. Electrochemical cycling was subsequently performed at a constant rate of 20 mA g^{-1} within a voltage window of 1.4–4.5 V. Throughout the experiment, Ar carrier gas was delivered at a steady flow rate of 15 cc min^{-1} via a mass flow controller (WIZ-701C-LF), ensuring that evolved gases were continuously swept into the mass spectrometer for real-time detection.

The b-values were determined based on the power-law relationship ($i = av^b$) between the peak current (i) and scan rate (v), where a and b are adjustable parameters associated with the electrochemical reaction kinetics [S7]. The capacitive and diffusion-controlled contributions were quantitatively analyzed using Dunn's method according to

$$i(V) = k_1(V)v + k_2(V)v^{1/2} \tag{S2}$$

,where $i(V)$ represents the current response at a given potential (V), and $k_1(V)v$ and $k_2(V)v^{1/2}$ correspond to the capacitive-controlled and diffusion-controlled current contributions, respectively, at the scan rate (v) [S8].

S1.8 Assembly of pouch cells

The aluminum tab (cathode terminal) and nickel tab (anode terminal) were attached to their corresponding electrodes via ultrasonic welding (GN-800, Gelon). A laminated structure was then assembled by stacking a 40 μm-thick lithium-coated copper foil (11 μm) with a separator and the prepared cathode. Following electrolyte injection, the pouch cell was vacuum-sealed within an argon-filled glove box.

S1.9 Computational method

Density functional theory (DFT) calculations were performed using the Gaussian 09 Program embedded in Materials Studio to investigate the molecular electronic properties of NH_2-PP, including the highest occupied molecular orbital (HOMO), lowest unoccupied molecular orbital (LUMO), and electrostatic potential (ESP) distributions [S9]. The molecular geometries were optimized using the Becke 3-parameter Lee–Yang–Parr (B3LYP) exchange-correlation function [S10, S11]. The 6-31G(d,p) basis set was used for all constituent atoms (C, H, and N), explicitly including six Cartesian d-functions (6d) and standard polarization functions [S12].

In addition, periodic DFT calculations were conducted using the Vienna *Ab initio* Simulation Package (VASP) to evaluate formation energies and electrochemical potentials [S13, S14]. The exchange-correlation interactions were described within the generalized gradient approximation (GGA) using the Perdew–Burke–Ernzerhof (PBE) functional [S15,

S16]. The electron-ion interactions were treated using the projector augmented wave (PAW) method [S17]. The valence electron configurations were explicitly defined as H ($1s^1$), Li ($1s^2$ $2s^1$), C ($2s^2$ $2p^2$), N ($2s^2$ $2p^3$), F ($2s^2$ $2p^5$), and P ($3s^2$ $3p^3$). A plane-wave basis set with a kinetic energy cutoff of 500 eV was employed. The convergence criteria for electronic self-consistency and ionic relaxation were set to 10^{-6} eV and 0.02 eV Å^{-1}, respectively. Brillouin zone sampling was performed using a Γ-centered 1 × 1 × 1 k-point mesh for all structural models [S18].

To eliminate spurious interactions between periodic images, the molecular structures were placed at the center of a sufficiently large supercell, providing a vacuum spacing of at least 10 Å in the x, y, and z directions. Solvation effects were incorporated using the implicit solvation model implemented in VASPsol, thereby approximating the electrolyte environment relevant to battery operation [S19]. The electrolyte was modeled as a mixed solvent of ethylene carbonate (EC) and dimethyl carbonate (DMC) with a volume ratio of 3:7. To represent this solvent environment in the implicit solvation framework, the bulk dielectric constant (ε_b) was set to 18.5, in accordance with reported literature values [S20, S21]. The solvation model parameters were defined as follows: the cutoff charge density (n_c) was set to 0.0025 e Å^{-3}, and the dielectric cavity width (σ) was set to 0.6 Å. In addition, the effective Debye length (λ_D) was fixed at 1.35 Å, while the cavity surface-tension parameter (τ) was set to zero.

To identify thermodynamically stable intermediate phases and elucidate the realistic compositional pathway during the charge–discharge process, formation energies (E_f) were calculated and used to construct the convex hull. The formation energy for each composition was defined as follows:

$$E_f = E\left(\mathrm{Li}_x(\mathrm{PF}_6)_y{}^*\right) - E(*) - xE(\mathrm{Li}) - yE(\mathrm{PF}_6) \tag{S3}$$

Here, * denotes the pristine NH_2-PP host structure. E(*) and E($Li_x(PF_6)_y$) represent the total energies of the pristine host and the corresponding lithiated/doped complexes, respectively. With respect to the chemical potentials of the guest species, the reference energies were carefully calibrated to reflect the experimental conditions. Specifically, the chemical potential of lithium, μ_{Li}, was derived by assuming an equilibrium potential of 3.0 V versus Li/Li^+, as observed experimentally. Accordingly, the reference energy of the PF_6 species was determined from the calculated total energy of bulk $LiPF_6$ by subtracting the calibrated lithium energy, (i.e., $E(\mathrm{PF}_6) = E(\mathrm{LiPF}_6)_{bulk} - E(\mathrm{Li})$).

Based on the thermodynamically stable phases identified from the convex hull analysis, the theoretical discharge voltage profile was subsequently derived using the following relation:

$$V = \frac{E\left(\mathrm{Li}_x(\mathrm{PF}_6)_y{}^*\right) - E\left(\mathrm{Li}_{x+\Delta x}(\mathrm{PF}_6)_{y+\Delta y}{}^*\right) + \Delta x E(\mathrm{Li}) + \Delta y E(\mathrm{PF}_6)}{n} \tag{S4}$$

In this equation, V denotes the average voltage of the cell. The terms $E\left(\mathrm{Li}_x(\mathrm{PF}_6)_y{}^*\right)$ and $E\left(\mathrm{Li}_{x+\Delta x}(\mathrm{PF}_6)_{y+\Delta y}{}^*\right)$ represent the total energies of the porphyrin-based complex before and after the uptake of charge carriers, respectively. The variables Δx and Δy correspond to the precise changes in the number of Li cations and PF_6 anions during the electrochemical reaction

step. The parameter n indicates the number of electrons transferred during the reaction. The reference values $E(\mathrm{Li})$ and $E(\mathrm{PF_6})$ are consistent with those defined in the formation energy calculation previously outlined.

In order to ensure the comparability of the adsorption-energy analysis, both the NH_2-PP and LFP (010) adsorption models were subjected to vacuum conditions, with a vacuum spacing of 15 Å [S22]. Preliminary implicit-solvation calculations for the LFP (010) surface resulted in the detachment/protrusion of surface Li atoms, thereby yielding an ambiguous clean-surface reference state. Consequently, the solvation effect was excluded from the ultimate comparative adsorption calculations, thereby enabling a direct comparison of the adsorption behavior of representative electrolyte-related species on NH_2-PP and LFP under equivalent computational conditions.

S2 Supporting Figures

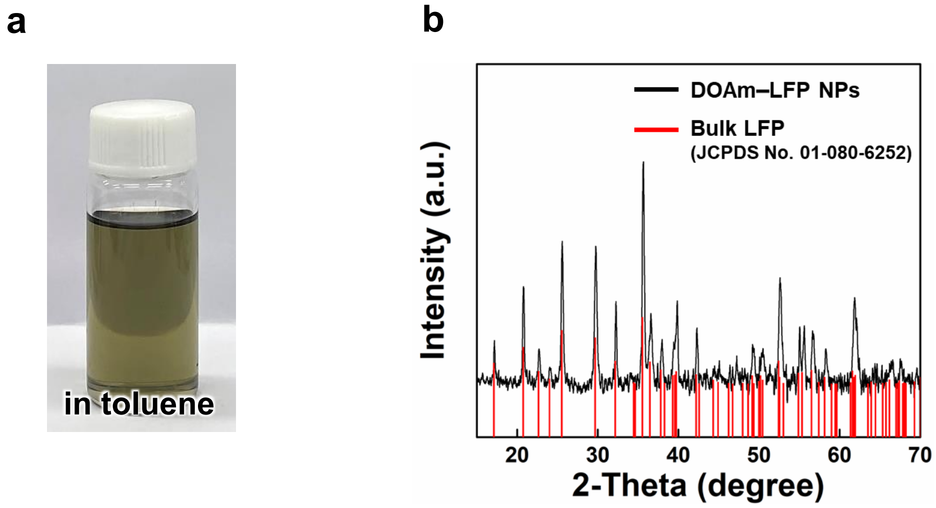

Fig. S1 Solution dispersibility and phase identification of DOAm-LFP NPs. a) Digital image of DOAm-LFP NP solution in toluene without any noticeable precipitation. b) X-ray diffraction (XRD) patterns of synthesized DOAm-LFP NPs and bulk $LiFePO_4$ crystals (JCPDS No. 01-080-6252). The XRD patterns of the DOAm-LFP NPs closely match the reference patterns of orthorhombic olivine-type $LiFePO_4$ (space group *Pnma*) with no additional diffraction peaks, indicating the high phase purity.

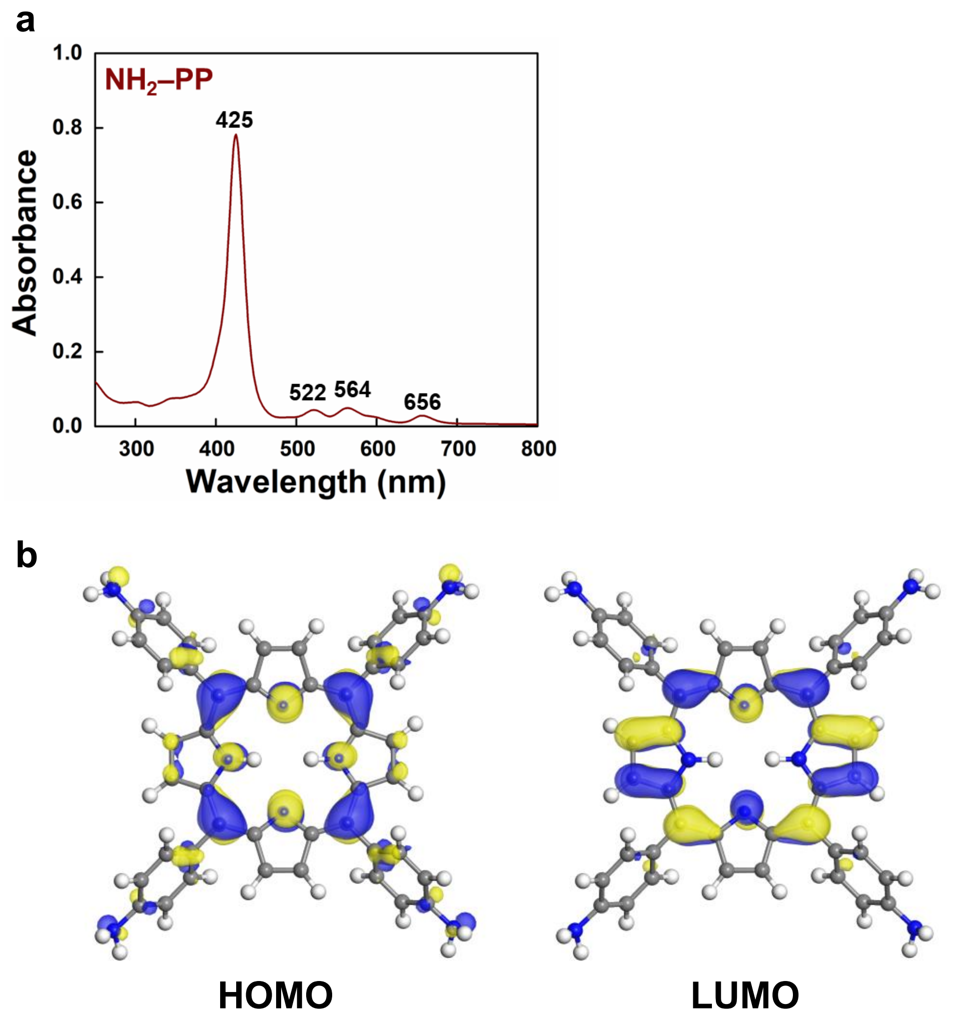

Fig. S2 Optical and electronic properties of NH_2-PP. a) UV-vis absorption spectra of NH_2-PP. In this case, the absorbance peaks corresponding to the Soret band (425 nm) and Q bands (522, 564, and 656 nm) originate from the macrocyclic ring of NH_2-PP. b) Frontier molecular orbitals of NH_2-PP molecule. It shows the electron-density distributions and energy levels of the HOMO and LUMO. In this case, the energy gap between HOMO and LUMO (ΔE) was calculated to be ~2.552 eV. (HOMO=-4.412 eV, LUMO=-1.860 eV).

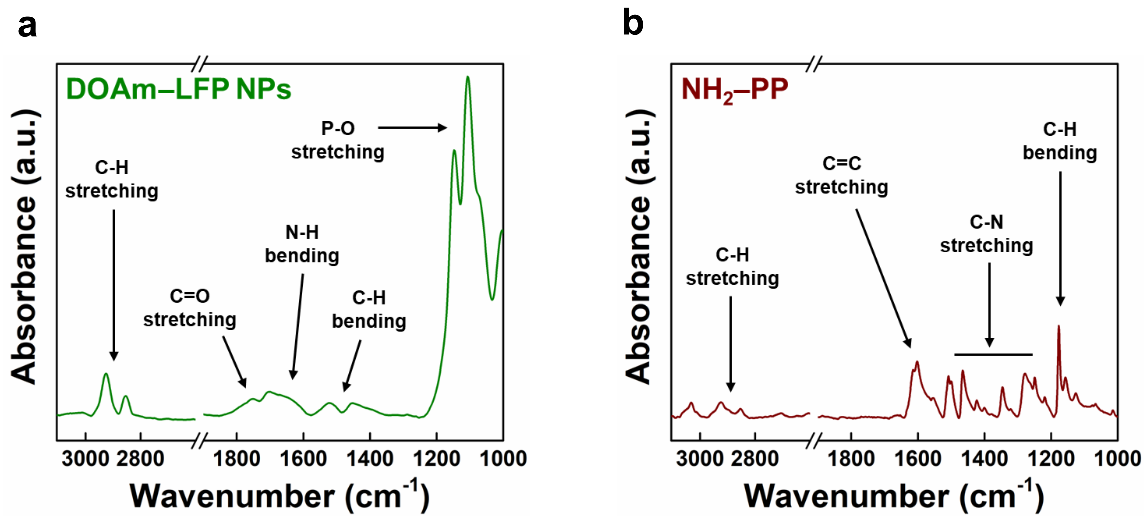

Fig. S3 FTIR analyses. FTIR analyses of a) Pristine DOAm-LFP NPs and b) NH_2-PP. The pristine DOAm ligand exhibits prominent vibrational features associated with its long aliphatic chains, including C-H stretching modes at ~2925 and 2854 cm^{-1}, C=O stretching at ~1749 cm^{-1}, N-H bending at ~1650 cm^{-1}, and C-H bending modes at ~1523 and 1452 cm^{-1}. In addition, strong bands corresponding to the phosphate groups of the LFP NP lattice (P-O stretching) are observed. In contrast, the NH_2-PP molecule displays distinct vibrational signatures, such as C=C stretching at ~1602 cm^{-1}, C-N stretching modes at ~1508, 1465, 1345, and 1278 cm^{-1}, and C-H bending at ~1176 cm^{-1}, which partially overlap with those of the DOAm ligand.

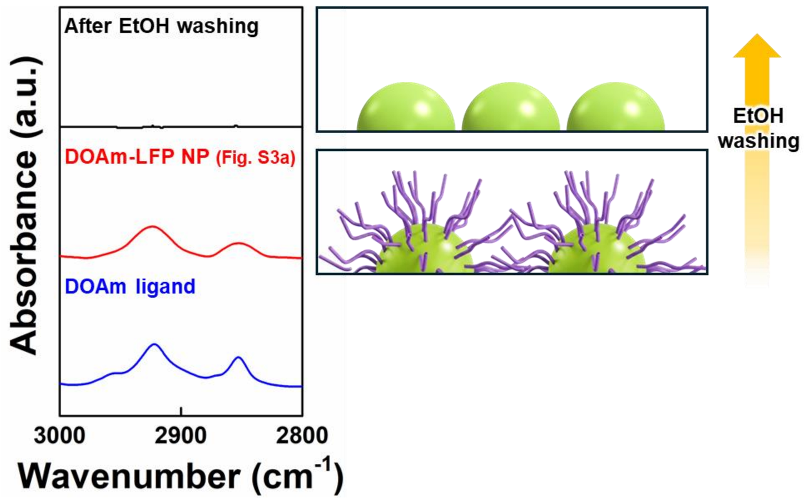

Fig. S4 Detachment of DOAm lignads. FT-IR analysis of pristine DOAm ligand (bottom), DOAm-LFP NP before EtOH washing (middle), and DOAm-removed LFP NP after EtOH washing (top). The significant reduction of the alkyl C-H stretching modes at ~2925 and 2854 cm^{-1} after EtOH washing indicates the effective removal of DOAm ligands from the LFP NP surface. The corresponding schematic illustration depicts the ligand-stabilized (middle) and ligand-removed surface (top) states before and after EtOH washing, respectively.

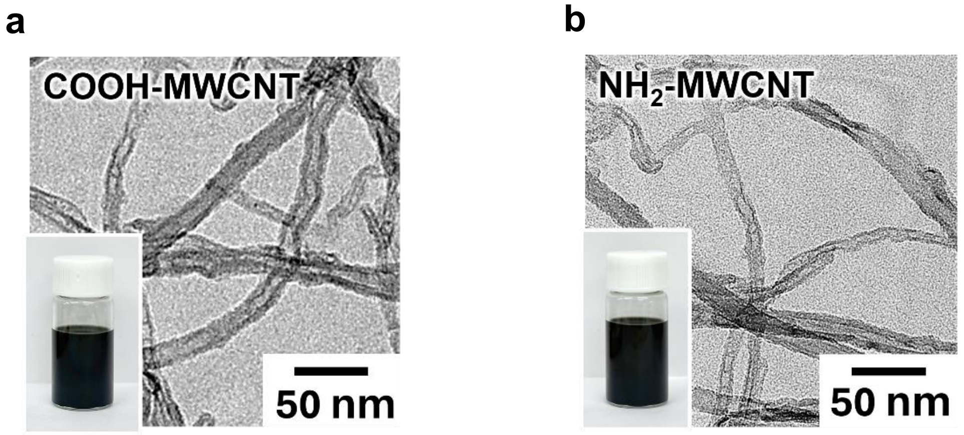

Fig. S5 Morphological characterization. HR-TEM images of a) COOH-MWCNT and b) NH_2-MWCNT. The inset images show the excellent dispersion behavior of COOH-MWCNT and NH_2-MWCNT in ethanol.

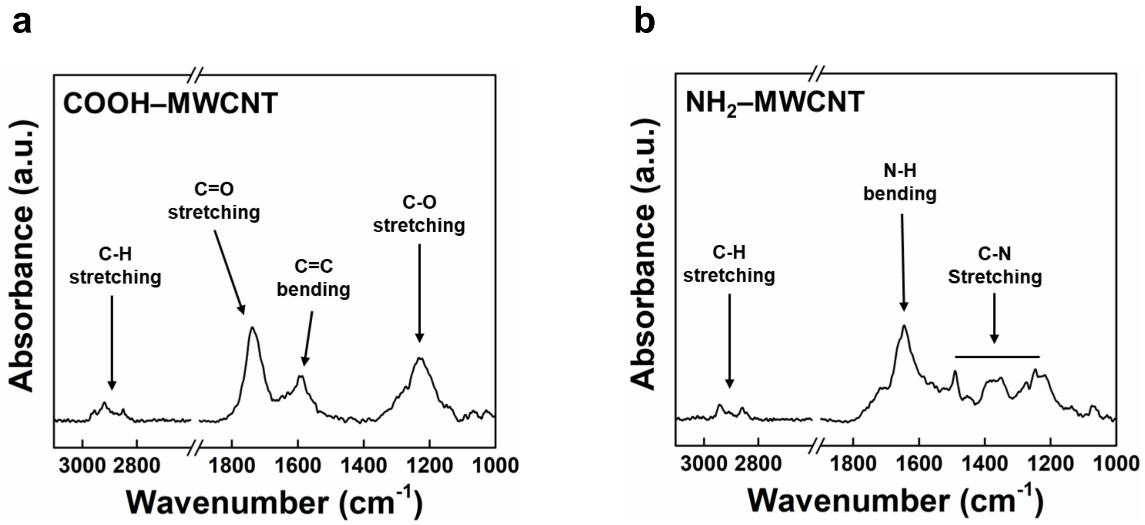

Fig. S6 FTIR analyses. FTIR analyses of a) COOH-MWCNT and b) NH_2-MWCNT. The COOH-MWCNT exhibits a characteristic vibrational band in the range of 1740–1700 cm^{-1}, corresponding to the C=O stretching vibration of carboxylic acid groups. In contrast, NH_2-MWCNT shows a pronounced absorption band in the 1650–1550 cm^{-1} region, which is attributed to N-H bending vibrations, including contributions from N-H, C-N, and $-NH_2$ modes.

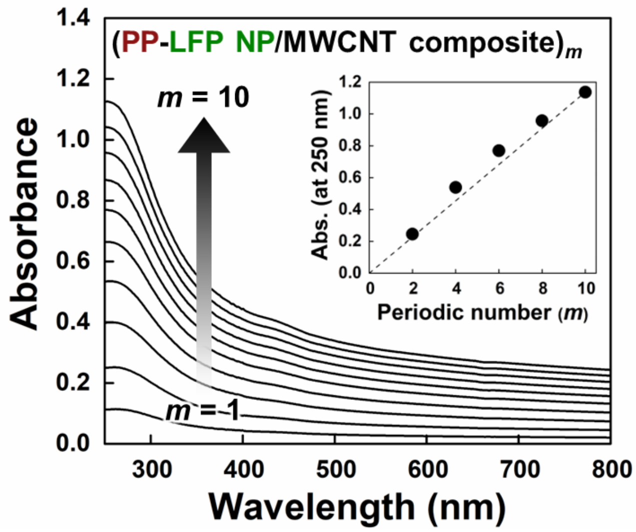

Fig. S7 UV-vis absorption spectra. UV-vis absorption spectra of the (PP-LFP NP/MWCNT composite)$_m$ multilayers as a function of periodic layer number (*m*). The inset shows the periodic layer number dependent-absorbance measured at 250 nm.

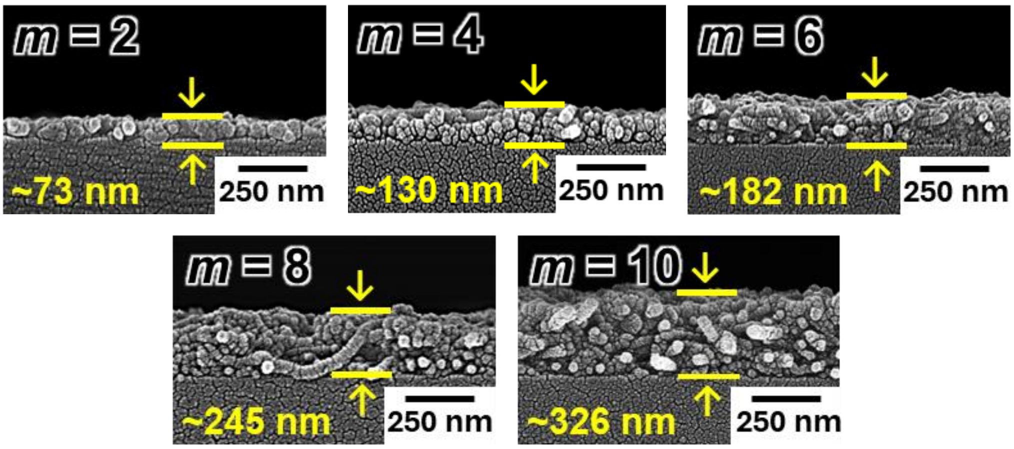

Fig. S8 Multilayer Thickness. Cross-sectional FE-SEM images of the (PP-LFP NP/MWCNT composite)$_m$ multilayers with different peridic number (m) of 2, 4, 6, 8, and 10.

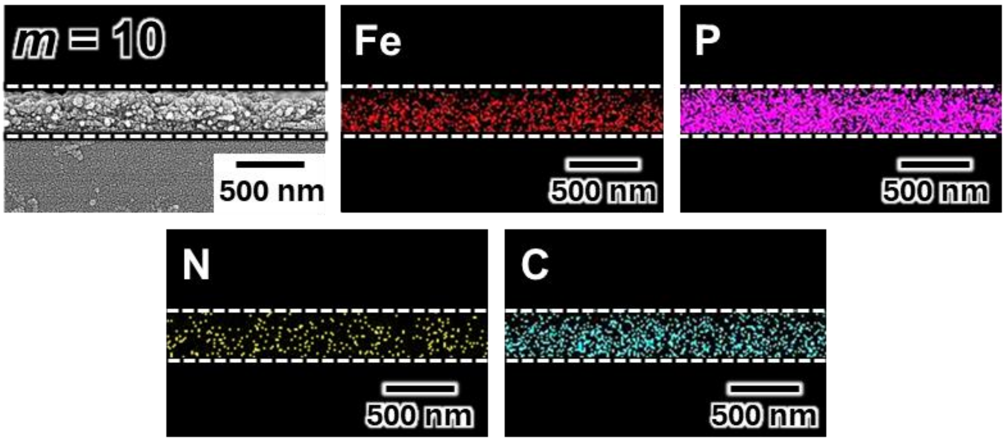

Fig. S9 Multilayer elemental distribution. Cross-sectional FE-SEM images and corresponding EDS elemental mapping images of the (PP-LFP NP/MWCNT composite)$_{10}$ multilayers. The images show the distributions of Fe, P, N, and C and confirming the well-stacked architecture of the (PP-LFP NP/MWCNT composite).

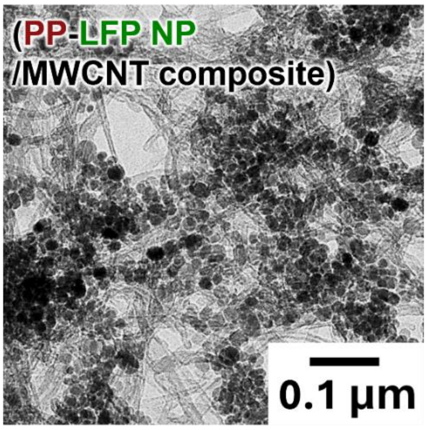

Fig. S10 HR-TEM image of the (PP-LFP NP/MWCNT composite) multilayers.

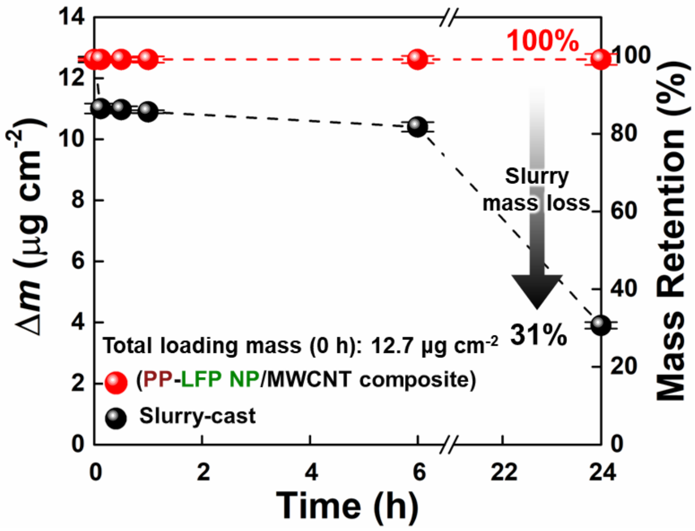

Fig. S11 QCM analysis. QCM analysis of mass retention for thermally annealed (PP-LFP NP/MWCNT composite) multilayers and slurry-cast films as a function of immersion time in the electrolyte. The initial areal loading mass of each sample was fixed at 12.7 μg cm^{-2}. The slurry-cast film exhibited a rapid mass loss within 24 h, retaining only 31% (3.9 μg cm^{-2}) of its initial mass. In contrast, the thermally annealed (PP-LFP NP/MWCNT composite) multilayers showed complete (100%) mass retention throughout the entire immersion period, indicating robust structural integrity and strong interfacial adhesion among the constituent components.

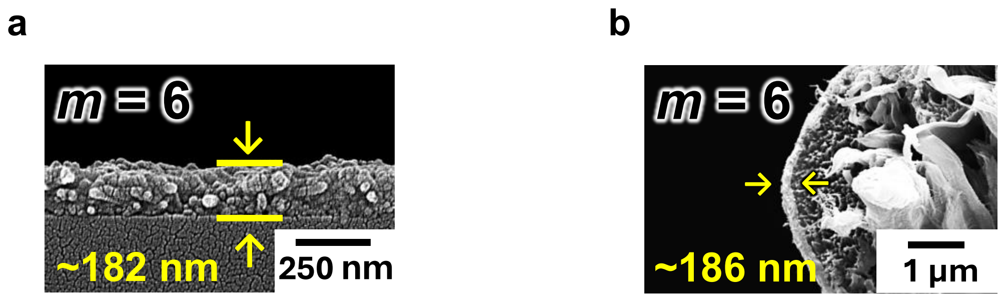

Fig. S12 Film thickness. Cross-sectional FE-SEM images of a) the (PP-LFP NP/MWCNT composite)$_6$ multilayers coated on the flat substrate and b) the (PP-LFP/MWCNT composite)$_6$ multilayers coated onto the textile current collector.

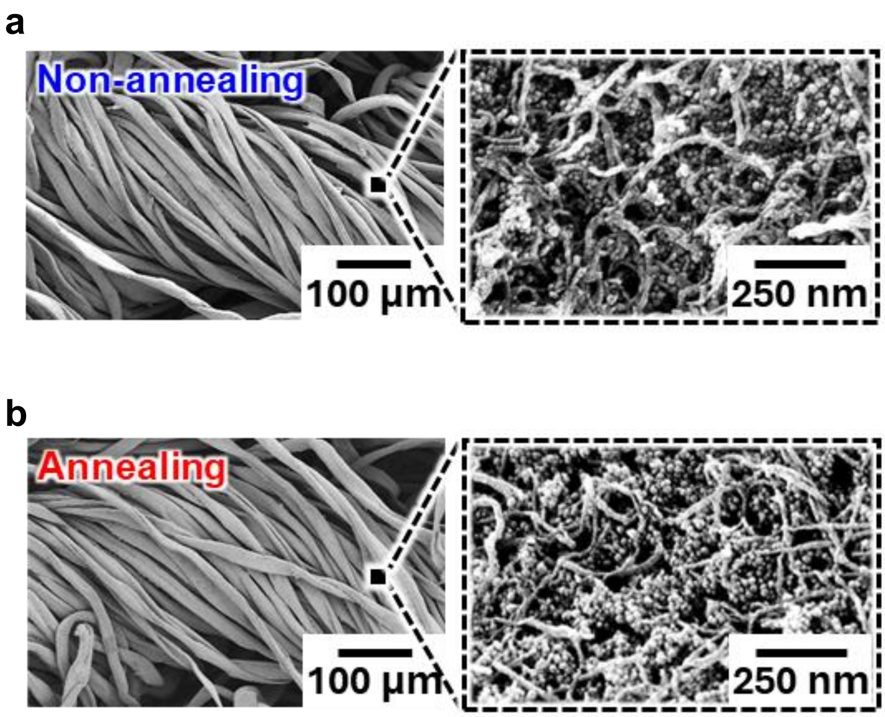

Fig. S13 Morphological characteristics. Planar FE-SEM images of the (PP-LFP NP/MWCNT composite)$_{10}$-coated textile electrodes a) without and b) with thermal annealing at 250 °C.

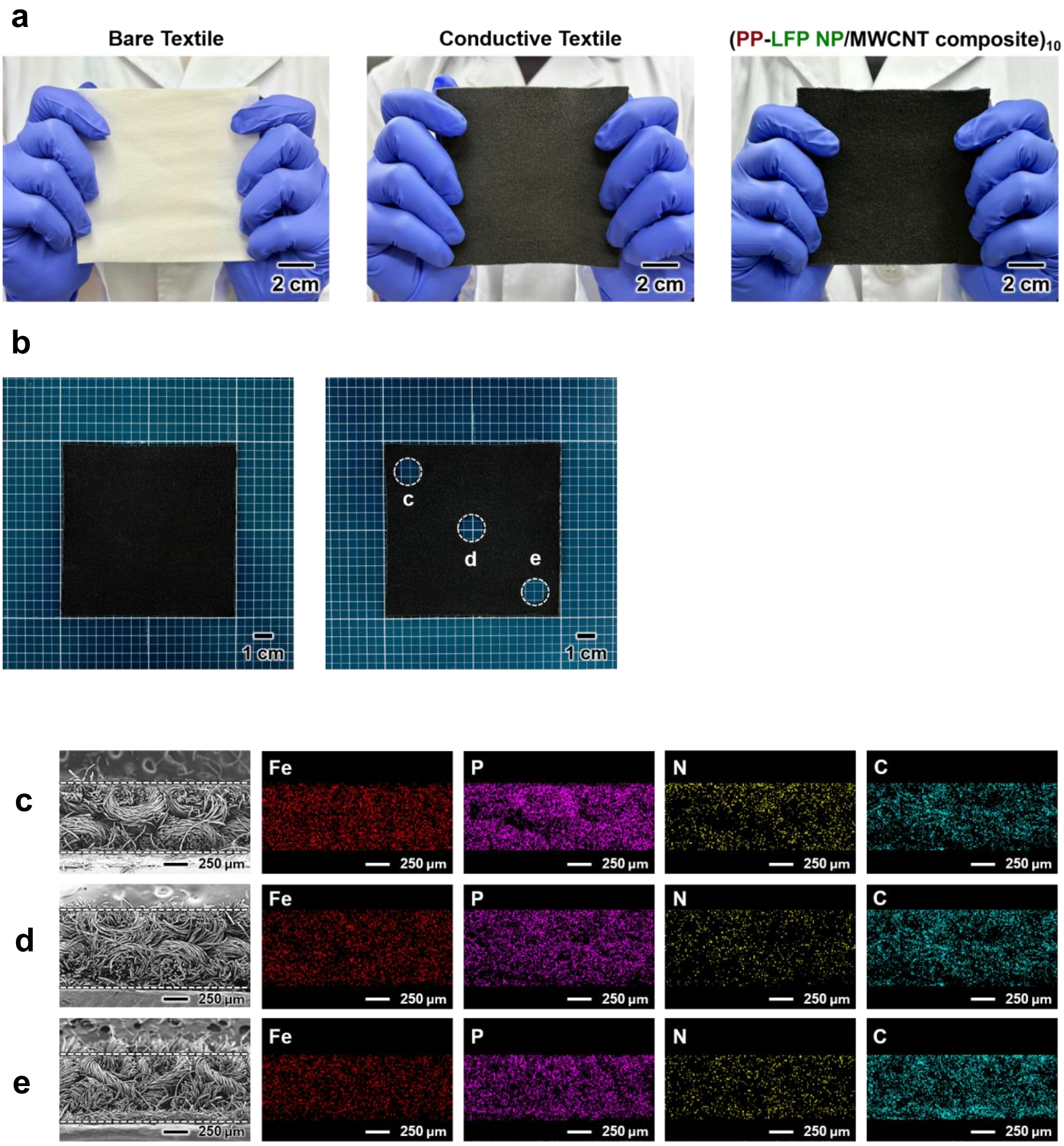

Fig. S14 Large-area textile electrode. a) Digital images of the 10 x 10 cm^2 bare textile, conductive textile, and the (PP-LFP NP/MWCNT composite)$_{10}$-coated textile electrode during the sequential deposition process. b) Digital image of three different regions punched from the 10 x 10 cm^2 (PP-LFP NP/MWCNT composite)$_{10}$-coated textile electrode. c-e) Cross-sectional FE-SEM and corresponding EDS mapping images obtained from the three different punched regions shown in b), confirming the highly uniform deposition and distribution of the electrode components throughout the large-area textile electrode.

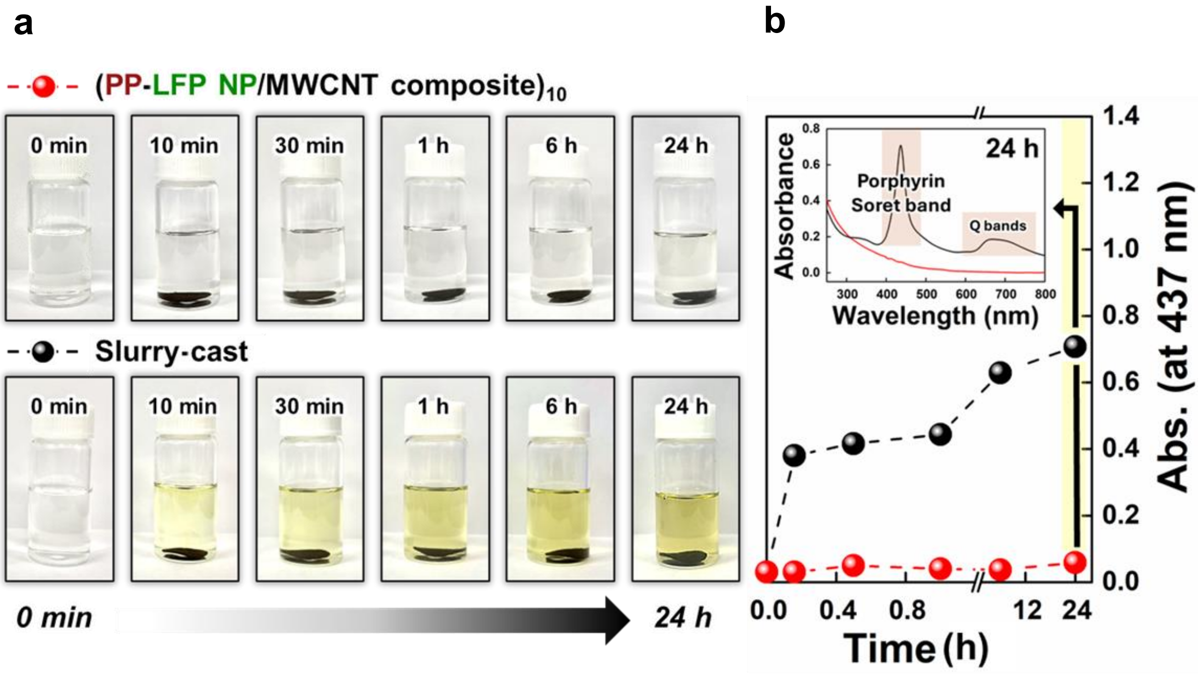

Fig. S15 Time-dependent dissolution behavior of porphyrin components. a) Digital images and b) UV-vis absorbance spectra showing the time-dependent dissolution behavior of porphyrin components from the (PP-LFP NP/MWCNT composite)$_{10}$-coated textile electrode and a slurry-cast textile electrode during electrolyte immersion. Upon immersion, the slurry-cast textile electrode rapidly released PP into the electrolyte, producing a green coloration and a pronounced Soret band at 437 nm, characteristic of porphyrin species. In contrast, the (PP-LFP NP/MWCNT composite)$_{10}$ multilayers on the textile substrate maintained a clear electrolyte even after 24 h of immersion and exhibited no detectable porphyrin-related absorption features in the UV-vis spectra, demonstrating effective suppression of porphyrin dissolution.

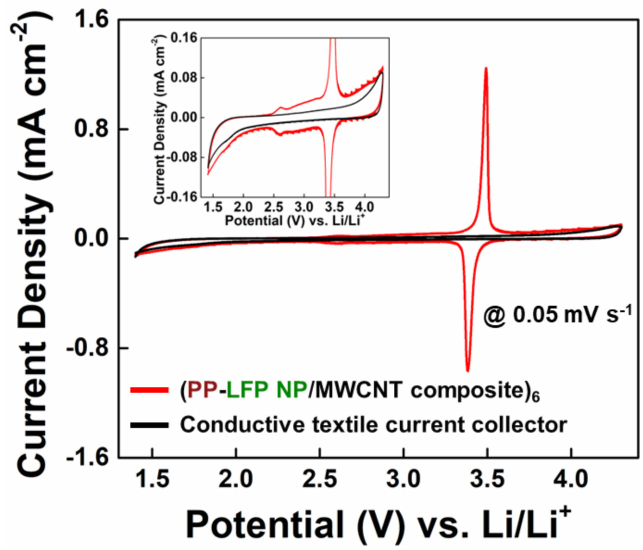

Fig. S16 Cyclic voltammetry (CV) curves. CV curves of the conductive textile current collector and the (PP-LFP NP/MWCNT composite)$_6$-coated textile electrode at a scan rate of 0.05 mV s^{-1} within a potential window of 1.4–4.3 V (*vs*. Li/Li^+). The substantially larger current response observed after deposition of the (PP-LFP NP/MWCNT composite)$_6$ multilayers indicates that the dominant charge-storage contribution arises from the active multilayer coating rather than from the textile current collector.

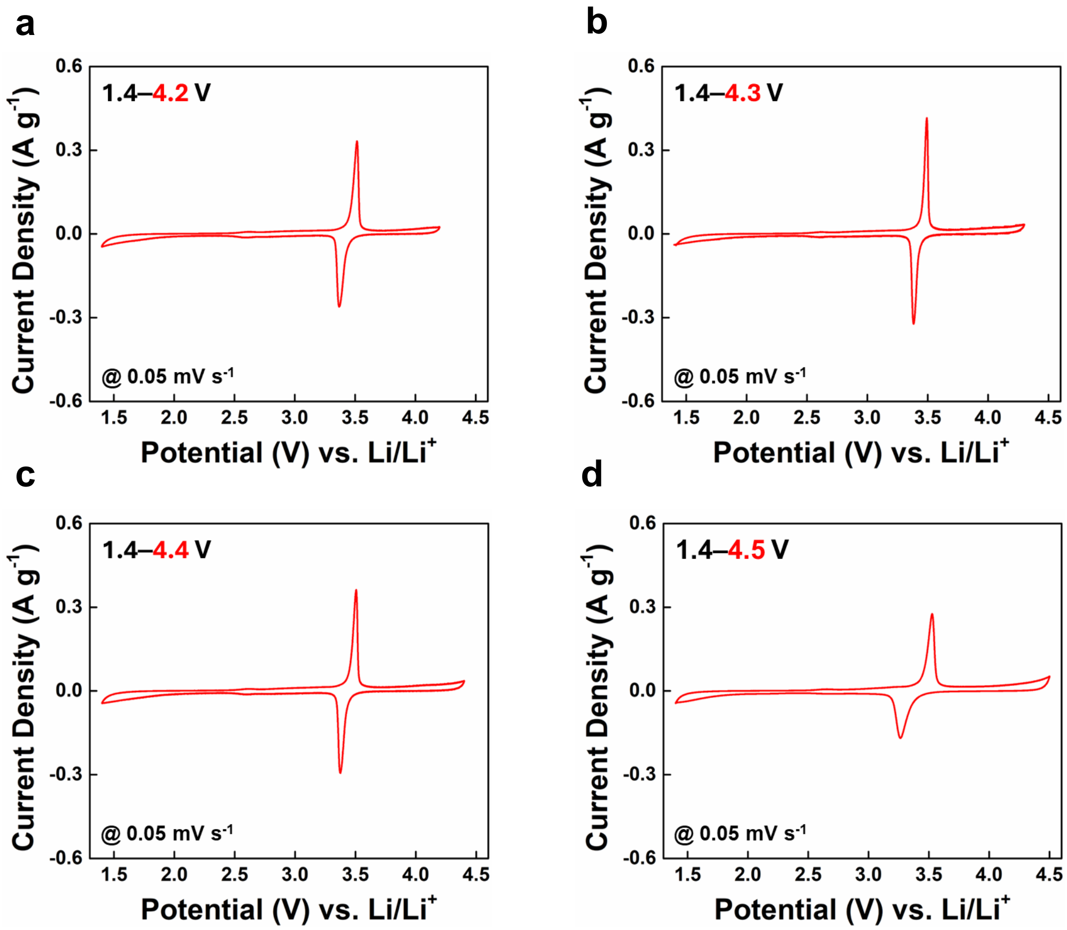

Fig. S17 CV curves with varying upper cut-off voltage. CV curves of the (PP-LFP NP/MWCNT composite)$_6$-coated textile electrodes at a scan rate of 0.05 mV s^{-1} with varying upper cut-off potentials: a) 4.2 V, b) 4.3 V, c) 4.4 V and d) 4.5 V. The cells cycled with cut-off potentials of 4.2 and 4.3 V exhibited stable and reversible electrochemical behavior, whereas those operated above 4.4 V showed signs of irreversible redox reactions, accompanied by larger ΔE_p values, indicative of increased internal resistance. On the basis of these observations, the operating potential window for the (PP-LFP NP/MWCNT composite)$_m$ textile cathodes was defined as 1.4–4.3 V.

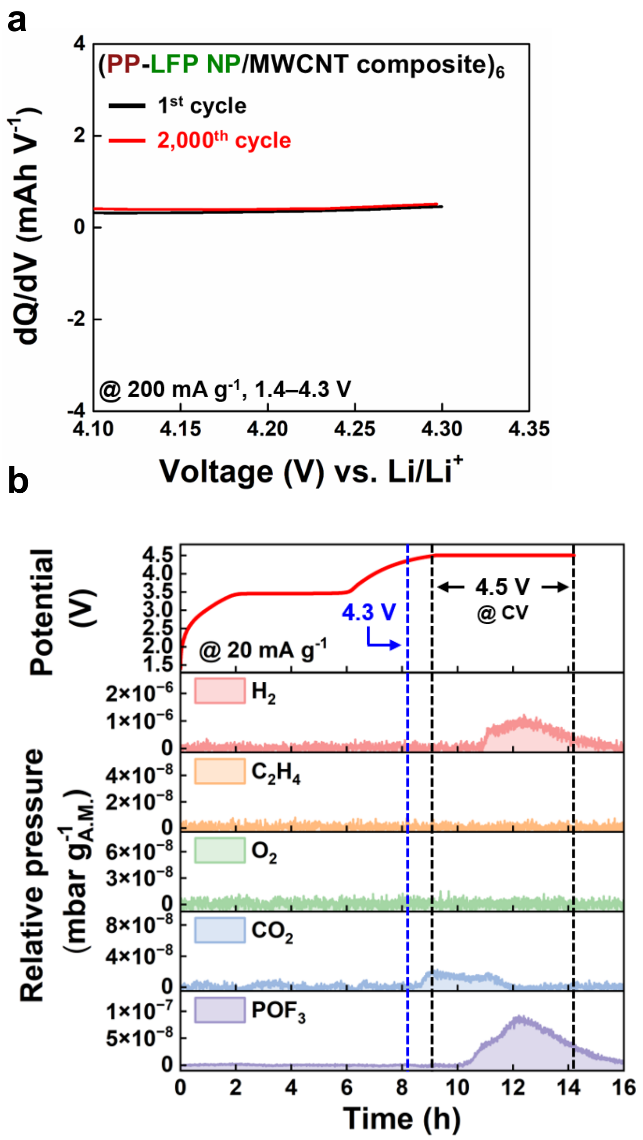

Fig. S18 dQ/dV profiles and Differential electrochemical mass spectrometry (DEMS) analysis. a) dQ/dV profiles of the (PP-LFP NP/MWCNT composite)$_6$-coated textile electrode performed at a current density of 200 mA g^{-1} recorded at the 1st and 2,000th cycles. b) DEMS profiles of the (PP-LFP NP/MWCNT composite)$_6$ textile electrode recorded during electrochemical cycling up to 4.3 V, showing negligible gas evolution and minimal electrolyte decomposition within the applied voltage window of 1.4–4.3 V (*vs.* Li/Li$^+$).

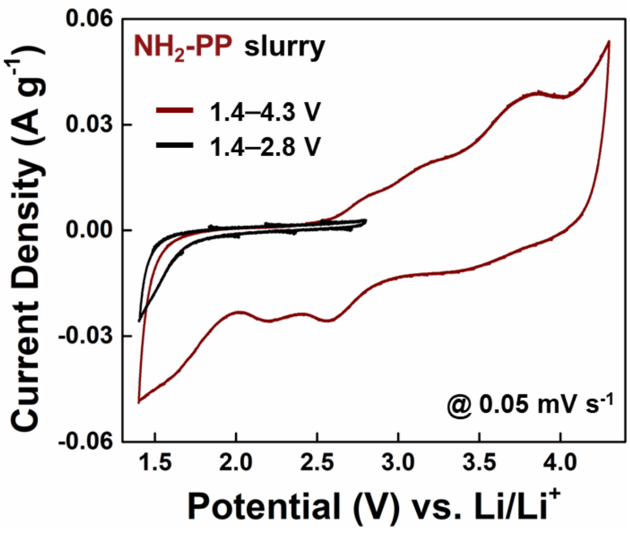

Fig. S19 CV curves. CV curves of NH_2-PP slurry-cast electrodes at a scan rate of 0.05 mV s^{-1} within different potential windows of 1.4–2.8 V and 1.4–4.3 V (*vs.* Li/Li^+). When cycled within a voltage window of 1.4–4.3 V, the electrode exhibited distinct oxidation peaks at 2.85, 3.15, and 3.8 V, along with corresponding reduction peaks at 2.2, 2.55, and 3.4 V [S23]. In contrast, operation within a narrower window of 1.4–2.8 V resulted in a substantially smaller enclosed area and the absence of well-defined redox peaks. These results confirm that NH_2-PP actively participates in the redox processes only within the extended voltage range of 1.4–4.3 V.

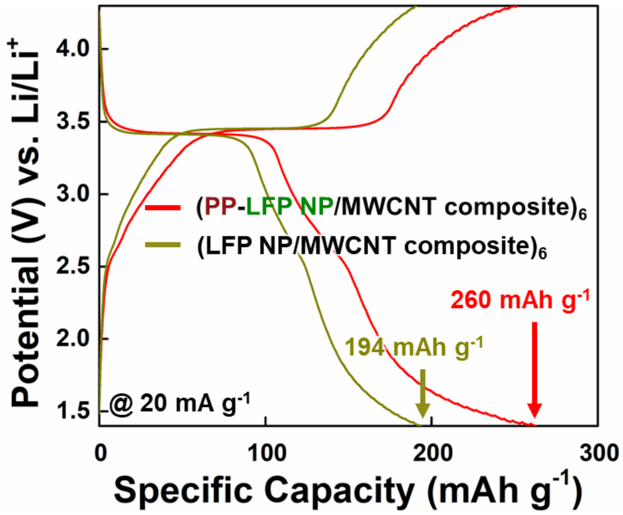

Fig. S20 Galvanostatic Charage-Discharge (GCD) profiles with and without NH_2-PP. GCD profiles of the (PP-LFP NP/MWCNT composite)$_6$-coated textile electrode and the PP-free (LFP NP/MWCNT composite)$_6$-coated textile electrode performed at a current density of 20 mA g^{-1}.

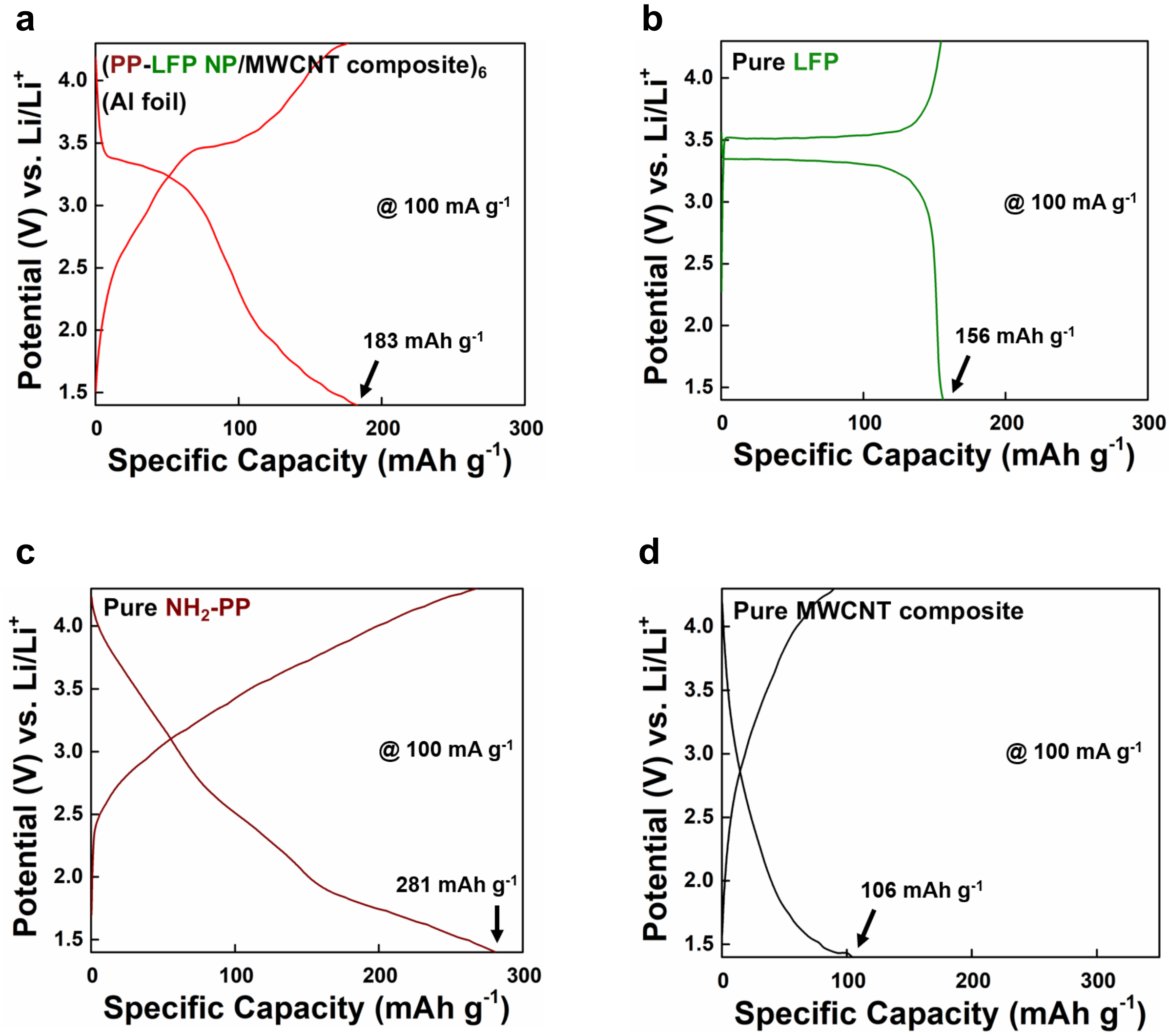

Fig. S21 Specific capacities of pure electrode components. GCD profiles of a) (PP-LFP NP/MWCNT composite)$_6$ multilayers on a flat 2D Al foil, b) pure LFP, c) pure NH_2-PP, and d) pure MWCNT composite electrodes using a conventional slurry casting method performed at a current density of 100 mA g^{-1}.

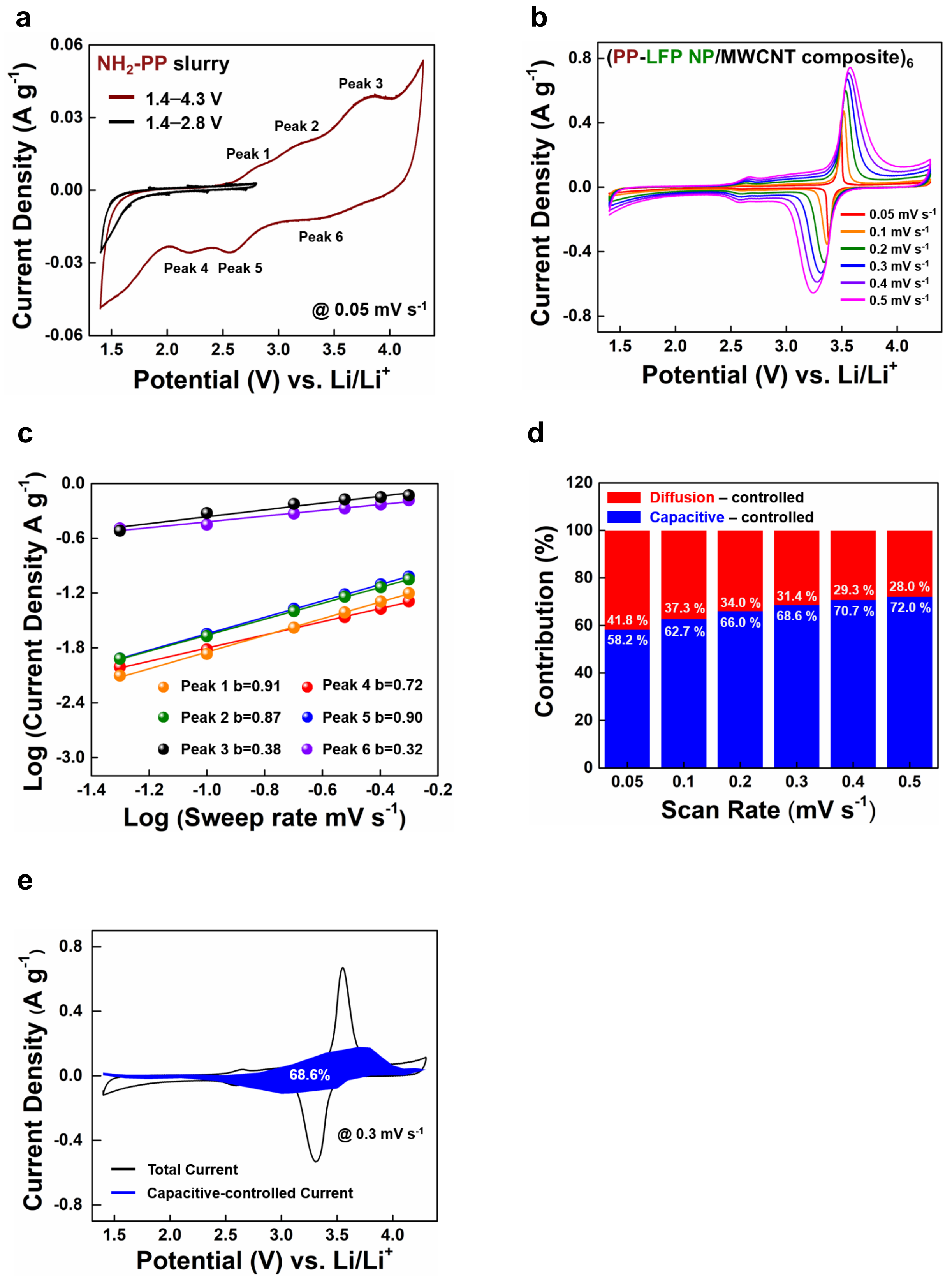
a
NH2-PP slurry
1.4–4.3 V
1.4–2.8 V
Peak 1
Peak 2
Peak 3
Peak 4
Peak 5
Peak 6
@ 0.05 mV s-1
Current Density (A g-1)
Potential (V) vs. Li/Li+
b
(PP-LFP NP/MWCNT composite)6
0.05 mV s-1
0.1 mV s-1
0.2 mV s-1
0.3 mV s-1
0.4 mV s-1
0.5 mV s-1
Current Density (A g-1)
Potential (V) vs. Li/Li+
c
Peak 1 b=0.91
Peak 2 b=0.87
Peak 3 b=0.38
Peak 4 b=0.72
Peak 5 b=0.90
Peak 6 b=0.32
Log (Current Density A g-1)
Log (Sweep rate mV s-1)
d
Diffusion – controlled
Capacitive – controlled
41.8 %
58.2 %
37.3 %
62.7 %
34.0 %
66.0 %
31.4 %
68.6 %
29.3 %
70.7 %
28.0 %
72.0 %
Contribution (%)
Scan Rate (mV s-1)
e
68.6%
@ 0.3 mV s-1
Total Current
Capacitive-controlled Current
Current Density (A g-1)
Potential (V) vs. Li/Li+

Fig. S22 Electrochemical kinetics of the (PP-LFP NP/MWCNT composite)$_6$-coated textile electrode. a) CV curves of NH_2-PP slurry-cast electrodes at a scan rate of 0.05 mV s^{-1} within different potential windows of 1.4–2.8 V and 1.4–4.3 V (*vs.* Li/Li^+). When cycled within a voltage window of 1.4–4.3 V, the electrode exhibited distinct oxidation peaks at 2.85, 3.15, and 3.8 V, along with corresponding reduction peaks at 2.2, 2.55, and 3.4 V [S23]. b) CV curves of the (PP-LFP NP/MWCNT composite)$_6$-coated textile electrode at various scan rates ranging from 0.05 to 0.5 mV s^{-1} within a potential window of 1.4–4.3 V (*vs.* Li/Li^+). c) Logarithmic plots of the (PP-LFP/WMCNT composite)$_6$-coated textile electrode within 1.4–4.3 V (*vs.* Li/Li^+). d) Scan rate-dependent charge contribution of the (PP-LFP/MWCNT composite)$_6$ electrode. e) Capacitive-controlled current contribution of the (PP-LFP NP/MWCNT composite)$_6$-coated textile electrode at a scan rate of 0.3 mV s^{-1}. The blue-colored region corresponds to the capacitive-controlled current contribution, accounting for ~68.6% of the total current response.

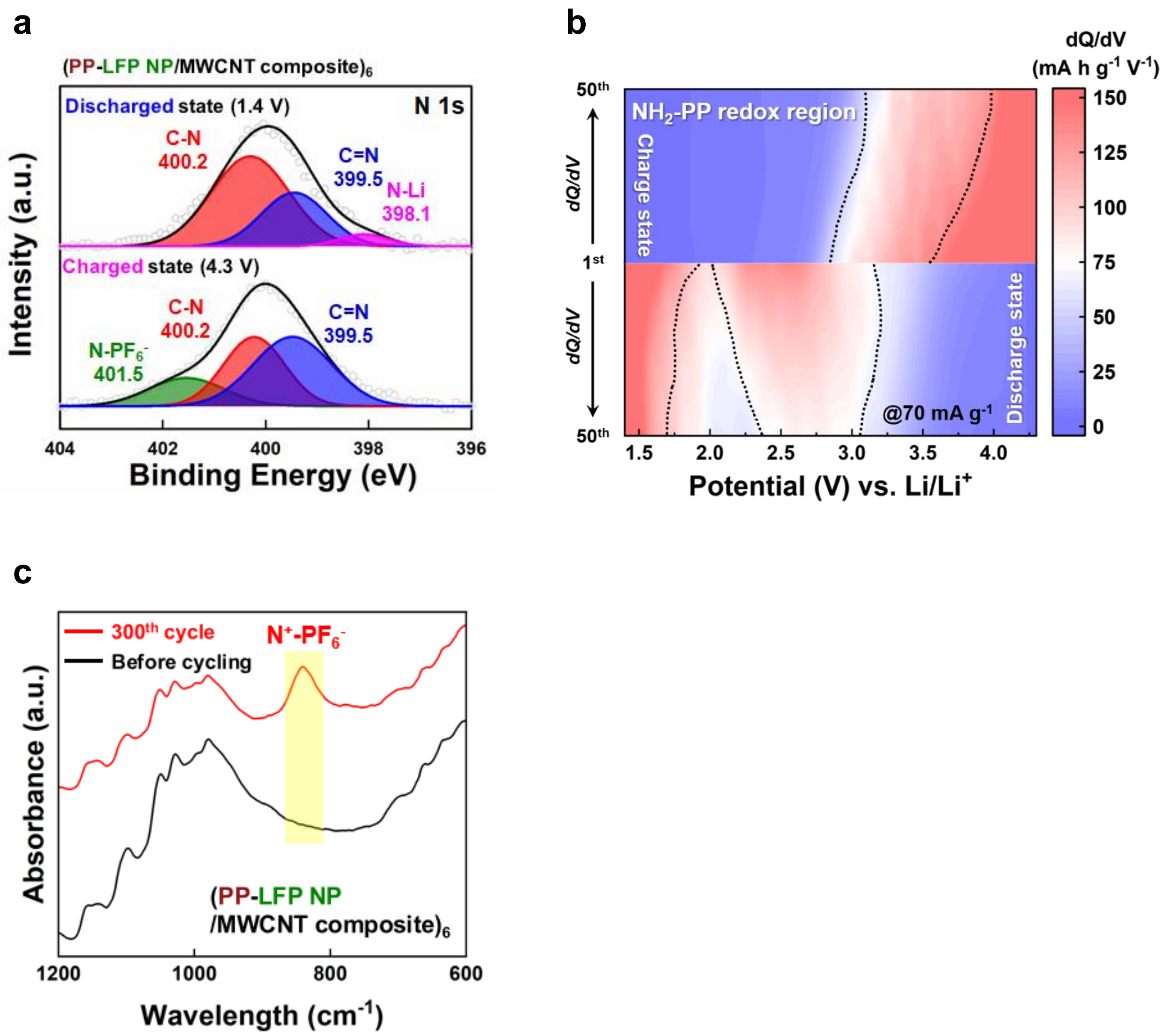

Fig. S23 Redox mechanism of NH_2-PP. a) Deconvoluted N 1s XPS spectra of the (PP-LFP NP/MWCNT composite)$_6$-coated textile electrode at the discharged (1.4 V) and charged (4.3 V) states after the 50th cycle performed at a current density of 200 mA g^{-1} within a potential window of 1.4–4.3 V (*vs*. Li/Li$^+$). b) dQ/dV profiles of the NH_2-PP slurry performed at a current density of 70 mA g^{-1} over 50 cycles. c) FTIR spectra evolution of (PP-LFP NP/MWCNT composite)$_6$-coated textile electrode before and after 300 cycles performed at a current density of 1,000 mA g^{-1} under a cut-off voltage of ~2.5 V.

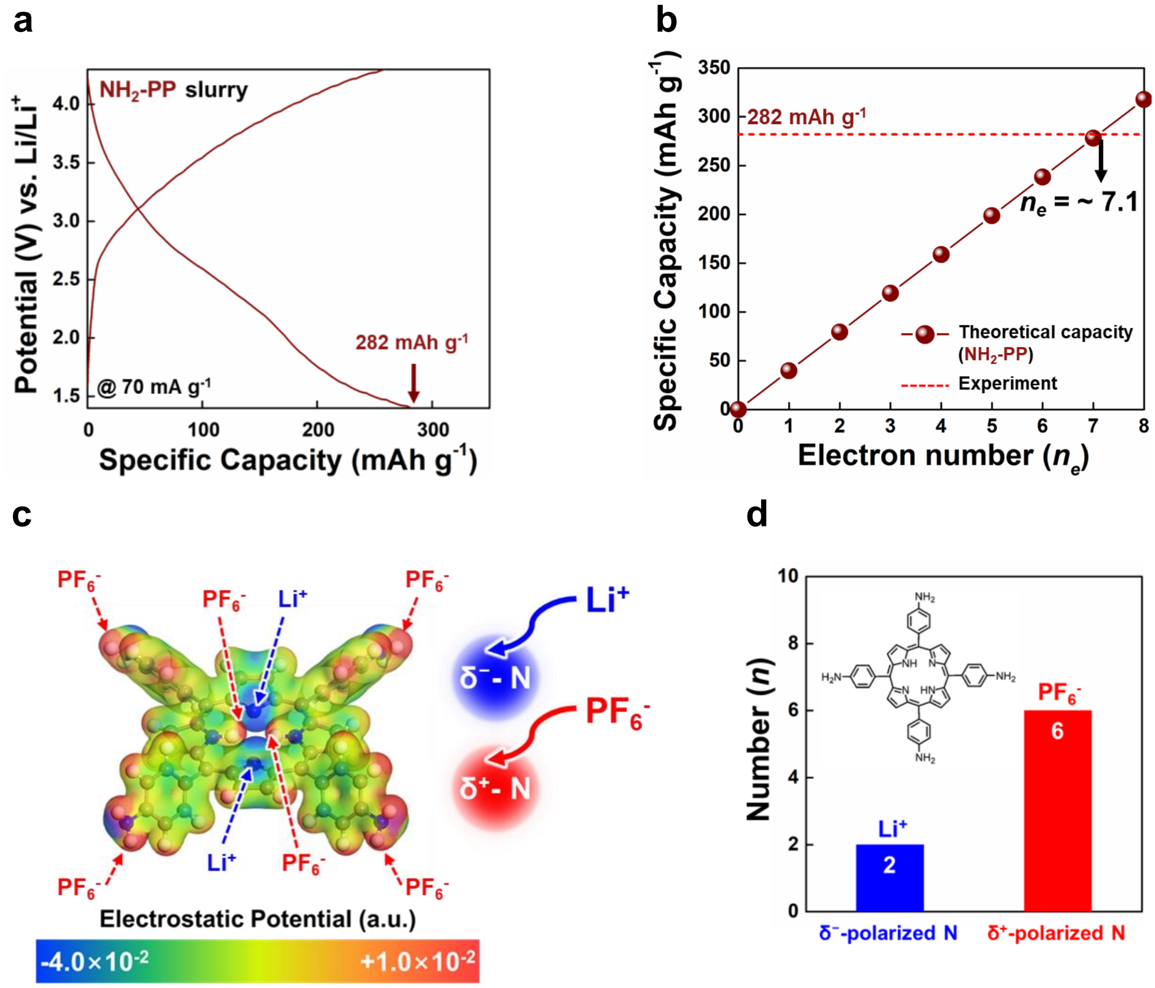

Fig. S24 Electrochemical analysis and DFT calculation of NH_2-PP. a) GCD profile of the NH_2-PP slurry-cast electrode performed at a current density of 70 mA g^{-1}. b) Estimation of the transferred electron number (n_e) for NH_2-PP based on the theoretical capacity analysis. The theoretical gravimetric capacity (C_{theo}) was calculated as follows: $C_{theo} = \frac{n \cdot F}{3.6\times M_w}$ c) Electrostatic potential mapping (ESP) illustrating the localized charge distribution (positive potentials; red and negative potentials; blue) and d) its electrostatic interaction with electrolyte species, highlighting the corresponding number of Li^+ and PF_6^- ions. From the measured discharge capacity of ~282 mAh g^{-1}, the corresponding electron transfer number was calculated to be ~7.1. Considering the multiple electrostatically active sites of NH_2-PP, the molecule is theoretically capable of accommodating up to eight electrons. In addition, two δ^--polarized nitrogen sites are identified as favorable coordination sites for Li^+ ions, whereas six δ^+-polarized regions associated with the porphyrin framework are involved in electrostatic interactions with PF_6^- ions.

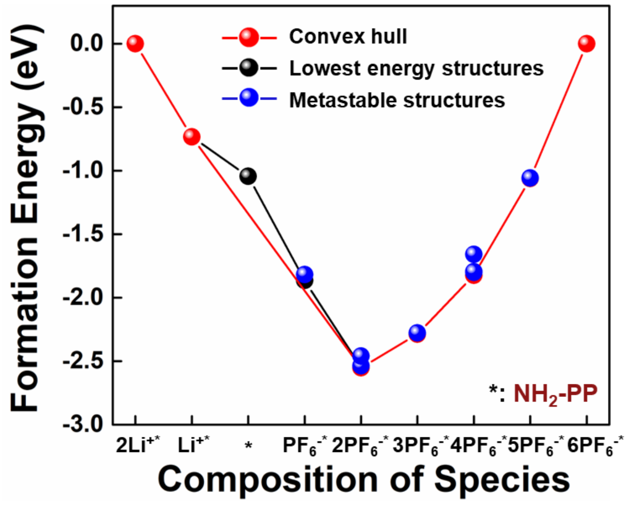

Fig. S25 Convex hull diagram calculated from formation energies. The red dot line defines the convex hull by connecting thermodynamically stable phases, representing the lowest-energy states accessible at each composition. The black dot line traces the minimum-energy structures identified at discrete composition, while the blue dots denote metastable isomers with different binding geometries. Notably, the pristine and one PF_6^--bound states are located above the convex hull, indicating thermodynamic instability with respect to phase separation into neighboring stable compositions. Consequently, these configurations do not constitute energetically favored intermediates under equilibrium conditions, leading to a direct transition between the doubly PF_6^--bound state and the Li^+-bound state along the thermodynamically preferred pathway.

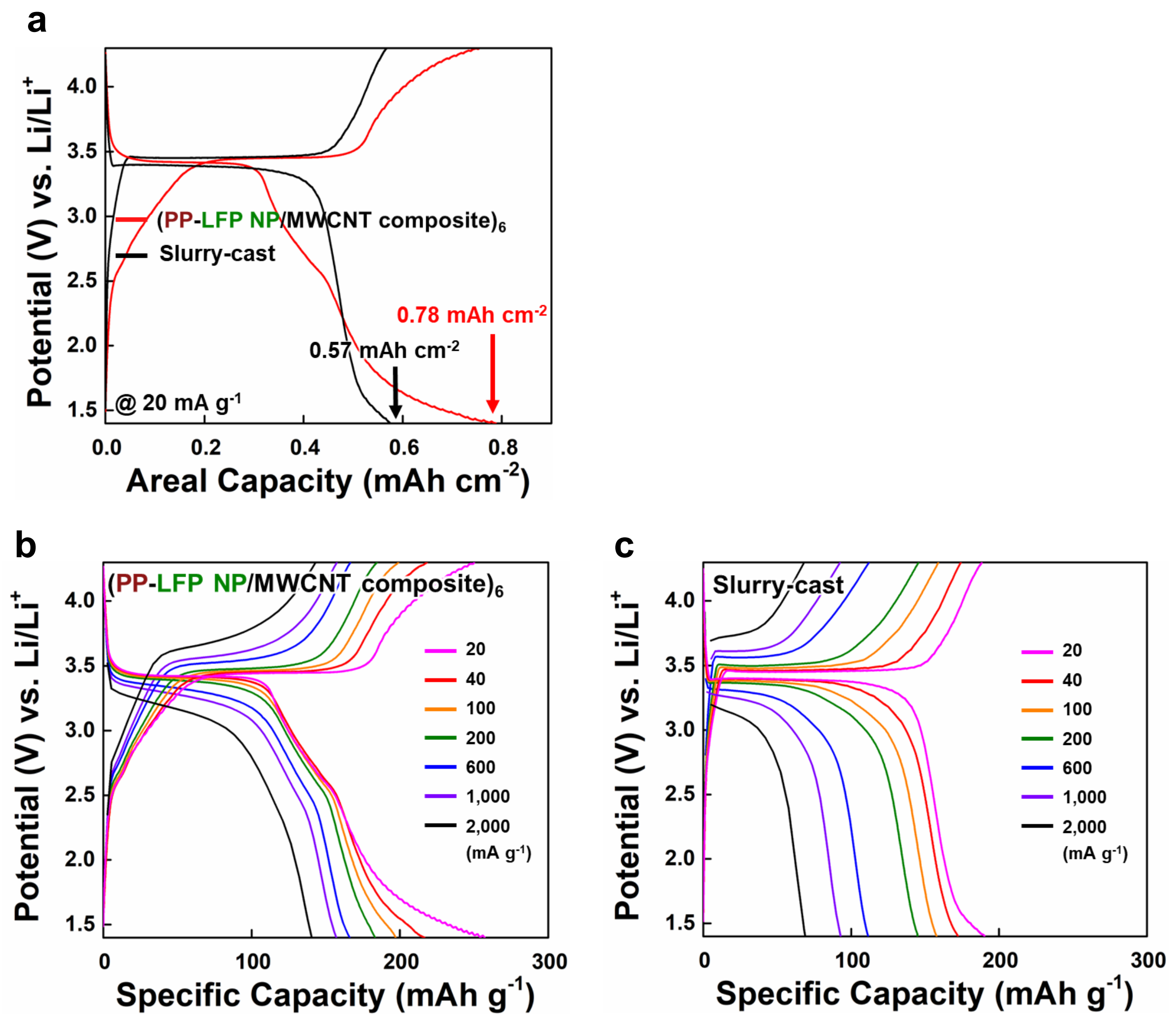

Fig. S26 Electrochemical analyses. a) GCD profiles of the (PP-LFP NP/MWCNT composite)$_6$-coated textile electrode and the slurry-cast electrode with the same loading mass (i.e., 3 mg cm^{-2}) performed at a current density of 20 mA g^{-1}. GCD profiles of b) the (PP-LFP NP/MWCNT composite)$_6$-coated textile electrode and c) the slurry-cast electrode performed at various current densities ranging from 20 to 2,000 mA g^{-1}.

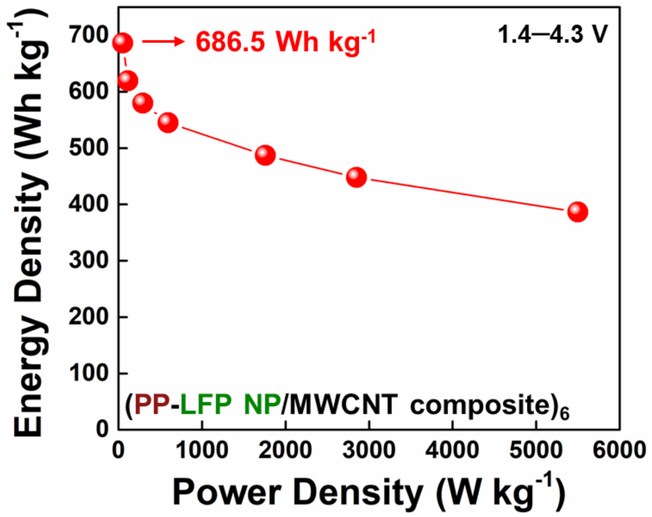

Fig. S27 Energy and power densities. Energy and power densities of the (PP-LFP NP/MWCNT composite)$_6$-coated textile electrode performed at various current densities ranging from 20 to 2,000 mA g^{-1}.

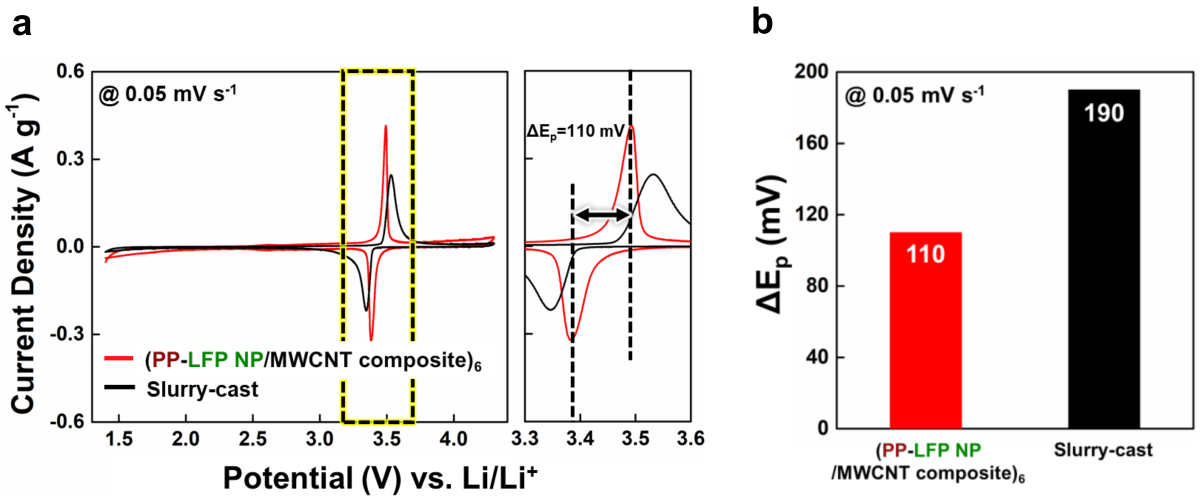

Fig. S28 CV curves and ΔE_p values. a) CV curves and b) ΔE_p values of the (PP-LFP NP/MWCNT composite)$_6$-coated textile electrode and the slurry-cast electrode at a scan rate of 0.05 mV s^{-1} in a potential window of 1.4–4.3 V (*vs.* Li/Li^+). The slurry-cast electrode exhibited broadened LFP redox peaks with a large peak-to-peak separation (ΔE_p = 190 mV), whereas the main electrode displayed much sharper and more well-defined peaks with a smaller ΔE_p of 110 mV, indicating more efficient charge-transfer kinetics.

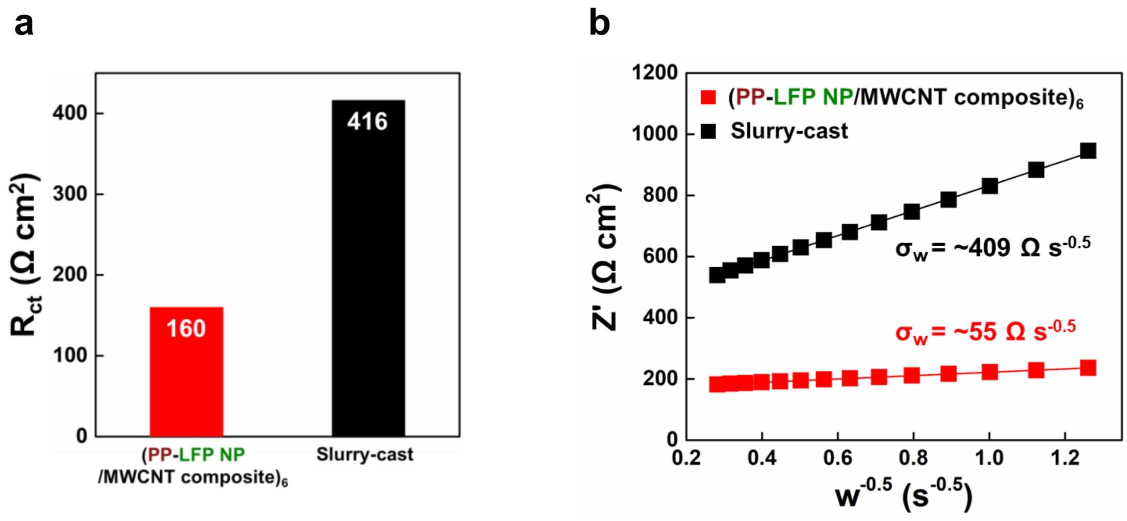

Fig. S29 Electrochemical impedance spectroscopy (EIS). a) Charge transfer resistance (R_{ct}) and b) Warburg impedance coefficient (σ_w) values of the (PP-LFP NP/MWCNT composite)$_6$-coated textile electrode and the slurry-cast electrode. In this case, the R_{ct} value of the (PP-LFP NP/MWCNT composite)$_6$-coated textile electrode was smaller than that of the slurry-cast electrode. This behavior is attributed to the well-defined ion-transport pathways and strong interfacial connectivity within the multilayer architecture. Accordingly, the σ_w value was calculated using the following equation: $Z' = R_s + R_{ct} + \sigma_w \omega^{-0.5}$

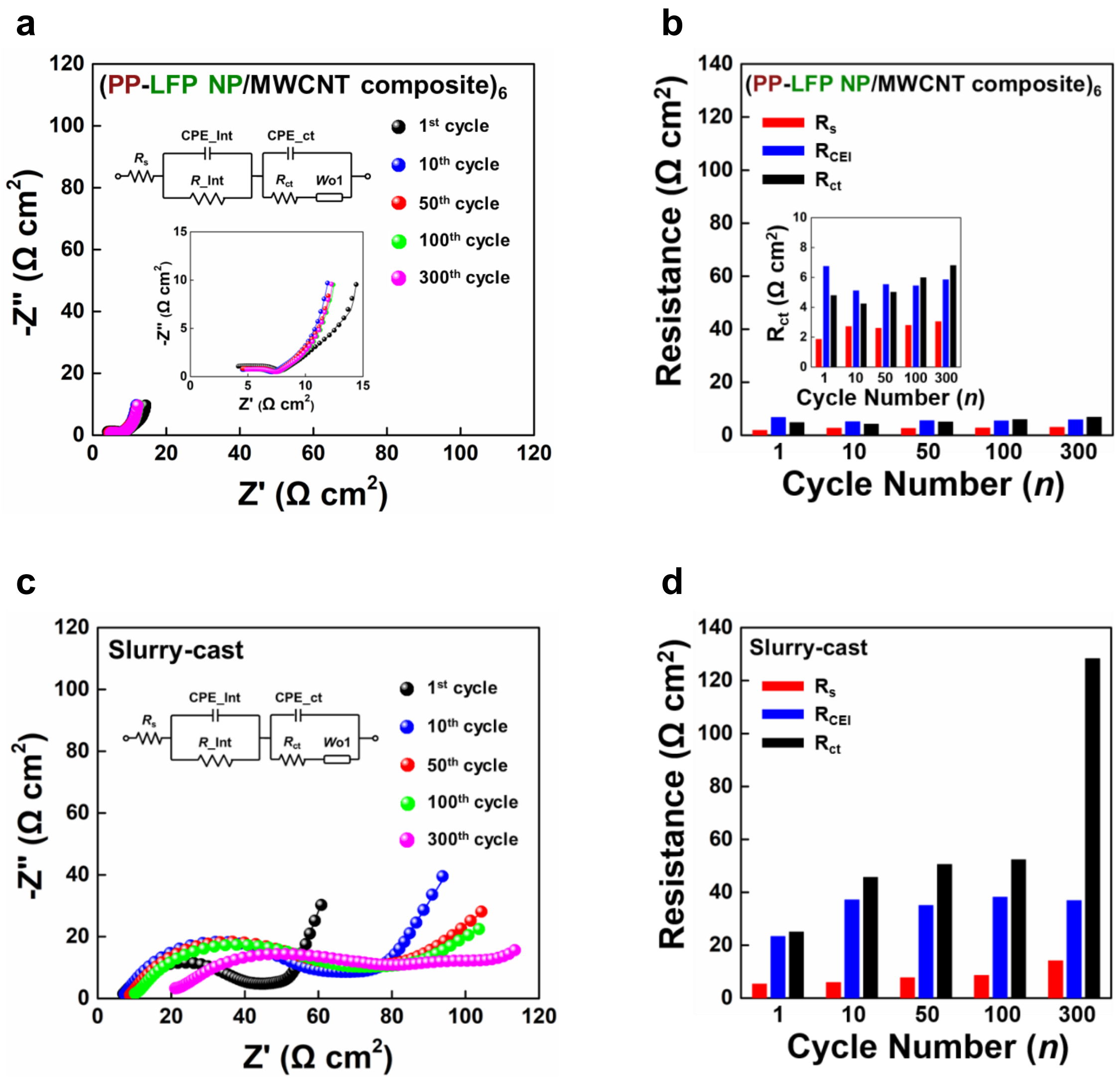

Fig. S30 Nyquist plots. Nyquist plots and Evolution of electrochemical impedance parameters of a, b) the (PP-LFP/MWCNT composite)$_6$ textile electrode and c, d) the slurry-cast electrode recorded at different electrochemical cycle number performed at a current density of 1,000 mA g^{-1}.

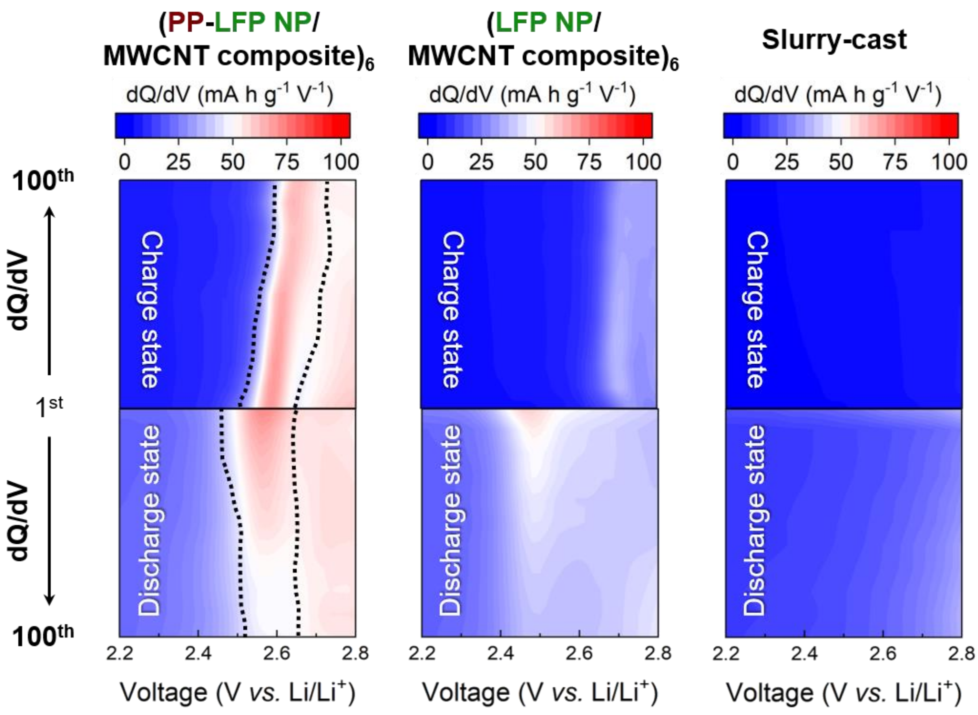

Fig. S31 dQ/dV plots. dQ/dV plots of the (PP-LFP NP/MWCNT composite)$_6$ electrode, the porphyrin-free (LFP NP/MWCNT composite)$_6$ electrode, and the slurry-cast electrode during cycling. The (PP-LFP NP/MWCNT composite)$_6$ electrode exhibited distinct additional redox evolution over a broad voltage region compared to the other two electrodes, indicating the electrochemical contribution of NH_2-PP enabled by the structurally integrated LbL-assembled electrode architecture.

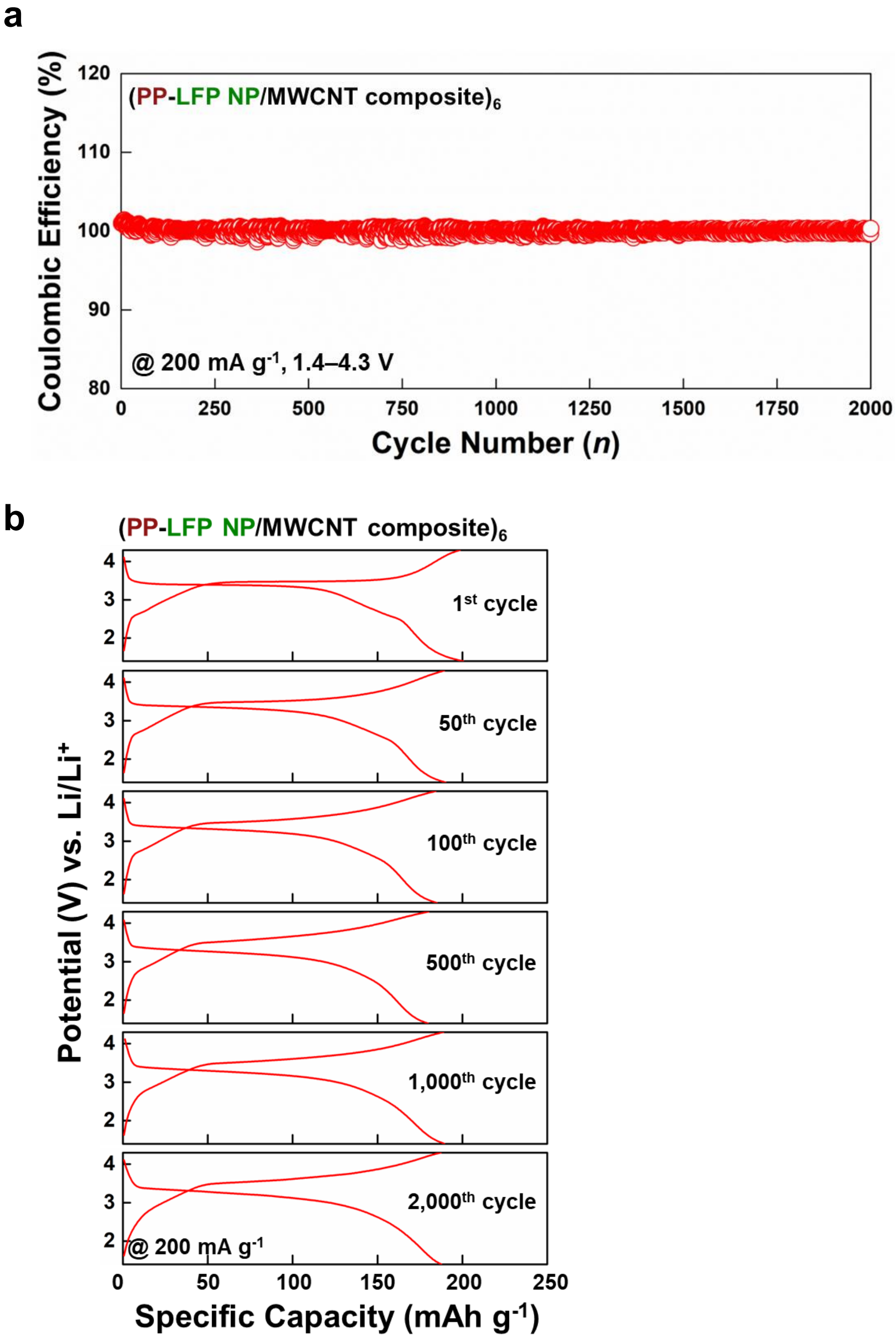

Fig. S32 Coulombic efficiency and GCD profiles. a) Coulombic efficiency profiles of the (PP-LFP NP/MWCNT composite)$_6$-coated textile electrode measured over 2,000 cycles at a current density of 200 mA g^{-1}, demonstrating highly stable Coulombic efficiency near 100% throughout prolonged cycling. b) GCD profiles of the (PP-LFP NP/MWCNT composite)$_6$-coated textile electrode recorded at the 1st, 50th, 100th, 500th, 1,000th, and 2,000th cycles performed at a current density of 200 mA g^{-1}.

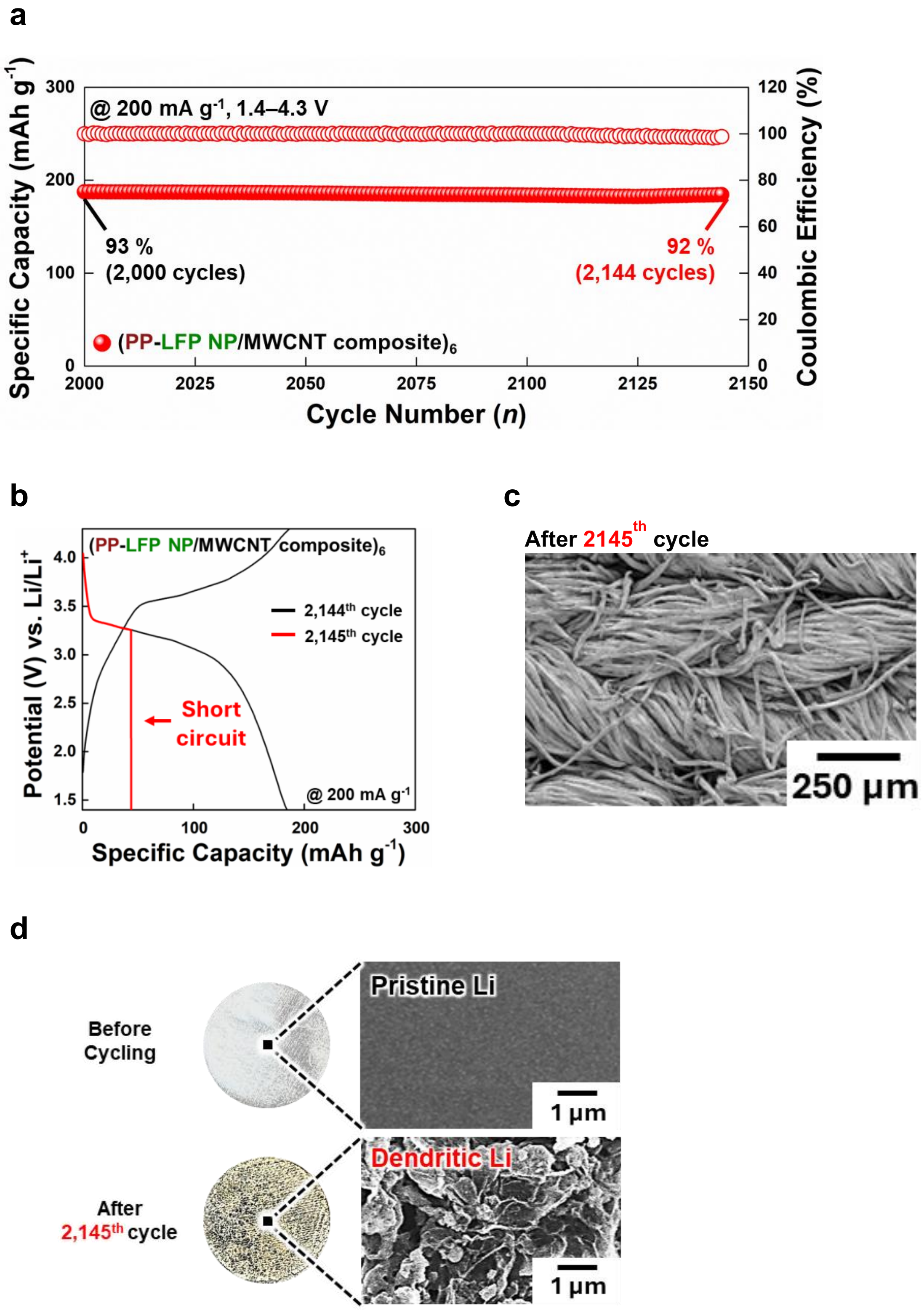
a
@ 200 mA g-1, 1.4–4.3 V
93 %
(2,000 cycles)
92 %
(2,144 cycles)
(PP-LFP NP/MWCNT composite)6
Specific Capacity (mAh g-1)
Coulombic Efficiency (%)
Cycle Number (n)
b
(PP-LFP NP/MWCNT composite)6
2,144th cycle
2,145th cycle
Short circuit
@ 200 mA g-1
Potential (V) vs. Li/Li+
Specific Capacity (mAh g-1)
c
After 2145th cycle
250 μm
d
Before Cycling
Pristine Li
1 μm
After 2,145th cycle
Dendritic Li
1 μm

Fig. S33 Failure analysis of the (PP-LFP NP/MWCNT composite)$_6$-coated textile electrode after prolonged cycling at 200 mA g^{-1}. a) Long-term cycling performance of the (PP-LFP NP/MWCNT composite)$_6$-coated textile electrode near the failure point at a current density of 200 mA g^{-1} within a voltage window of 1.4-4.3 V. b) GCD profiles of the electrode at the 2,144th and 2,145th cycles, showing abrupt failure caused by an internal short circuit. c) FE-SEM image of the (PP-LFP NP/MWCNT composite)$_6$-coated textile electrode after the 2,145th cycle, revealing the well-preserved framework after prolonged cycling. d) Digital and FE-SEM images of pristine Li metal anodes before cycling and cycled Li metal after the 2,145th cycle, respectively, showing pronounced dendritic Li growth after prolonged cycling.

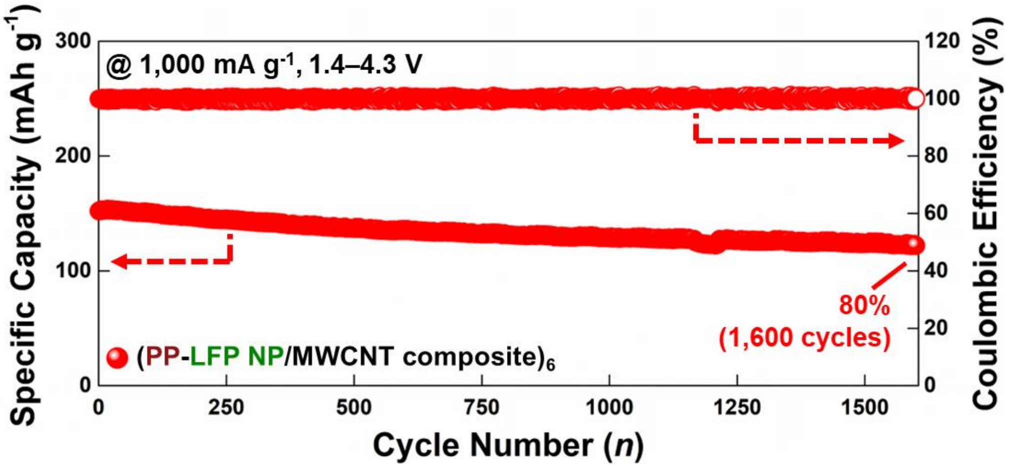

Fig. S34 Cycling performance. Cycling performance of the (PP-LFP NP/MWCNT composite)$_6$-coated textile electrode as a function of cycle number (n) performed at a current density of 1,000 mA g^{-1}.

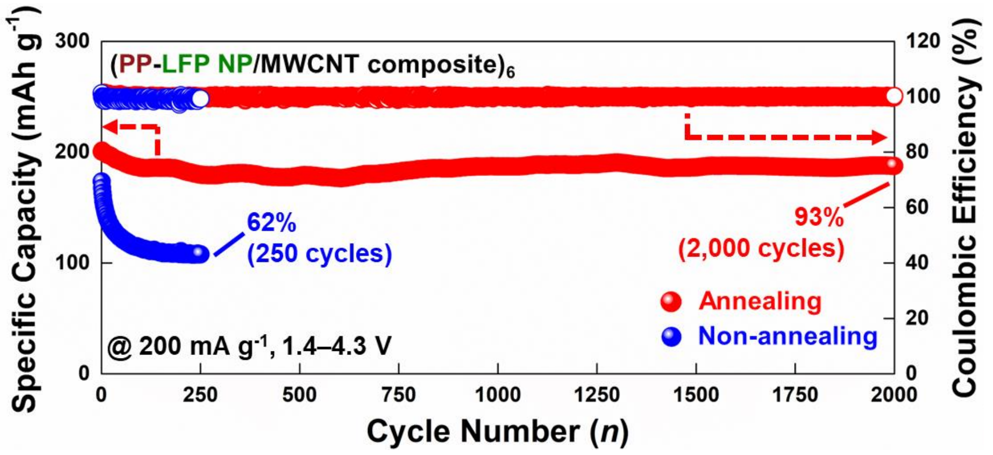

Fig. S35 Cycling performance. Comparison of the cycling performance of the (PP-LFP NP/MWCNT composite)$_6$-coated textile electrode without (blue) and with (red) thermal annealing performed at a current density of 200 mA g^{-1}.

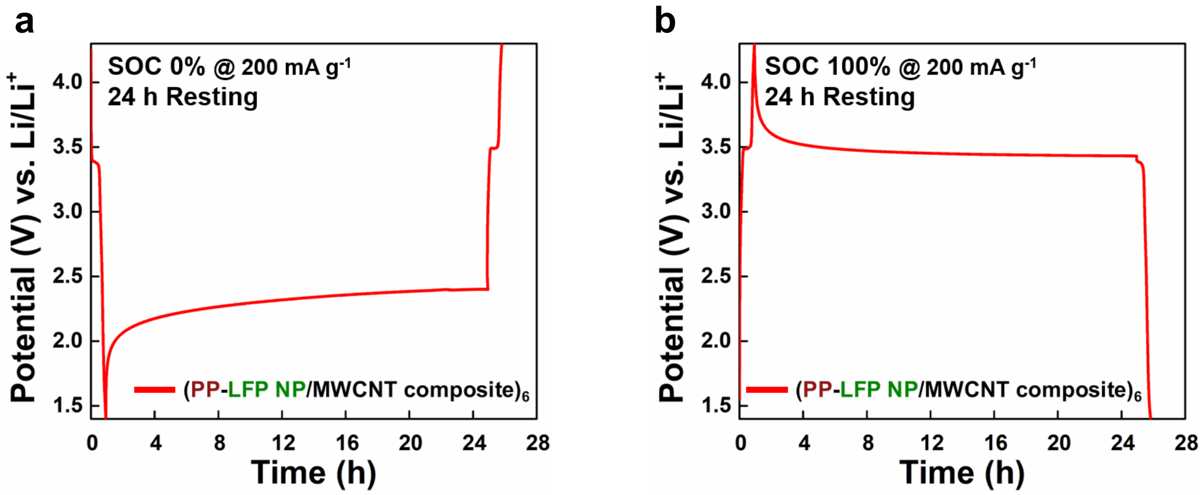

Fig. S36 Self-discharge behavior. Self-discharge behavior of the (PP-LFP NP/MWCNT composite)$_6$-coated textile electrodes evaluated by open-circuit voltage (OCV) retention measurements at different states of charge (SOC) performed at a current density of 200 mA g^{-1}. The cells were charged/discharged to a) 0% SOC and b) 100% SOC, followed by 24 h open-circuit resting while monitoring the voltage variation.

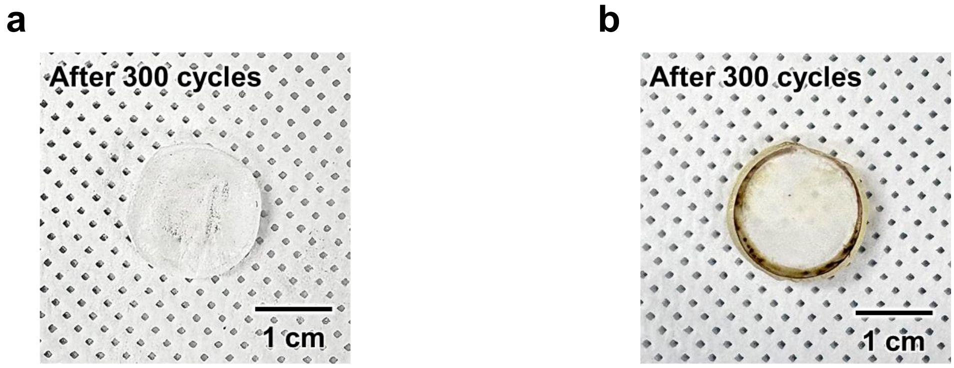

Fig. S37 Immobilization behavior of NH_2-PP. Digital images of separators disassembled from a) the (PP-LFP/MWCNT composite)$_6$ cell and b) slurry-cast cell after 300 cycles. The test of these cells was performed at a current density of 1,000 mA g^{-1}.

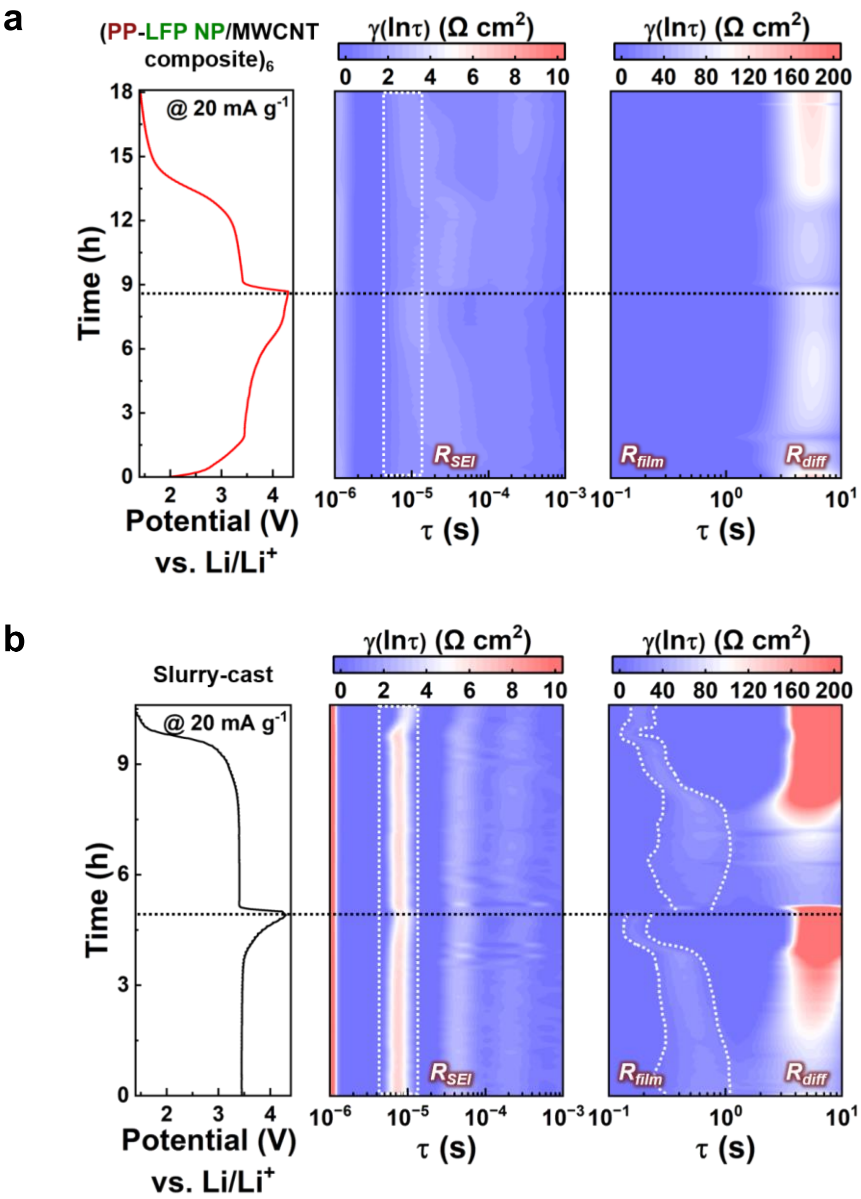

Fig. S38 Immobilization behavior of NH_2-PP. In-situ DRT analysis of Li metal anodes assembled with a) the (PP-LFP NP/MWCNT composite)$_6$ textile electrode (top) and b) the slurry-cast electrode (bottom) during cycling performed at a current density of 20 mA g^{-1} (after 300 cycles at 1,000 mA g^{-1}). The DRT contour plots reveal the evolution of the R_{SEI}, interfacial organic-film resistance (R_{film}), and Li-ion diffusion resistance (R_{diff}) during prolonged electrochemical operation.

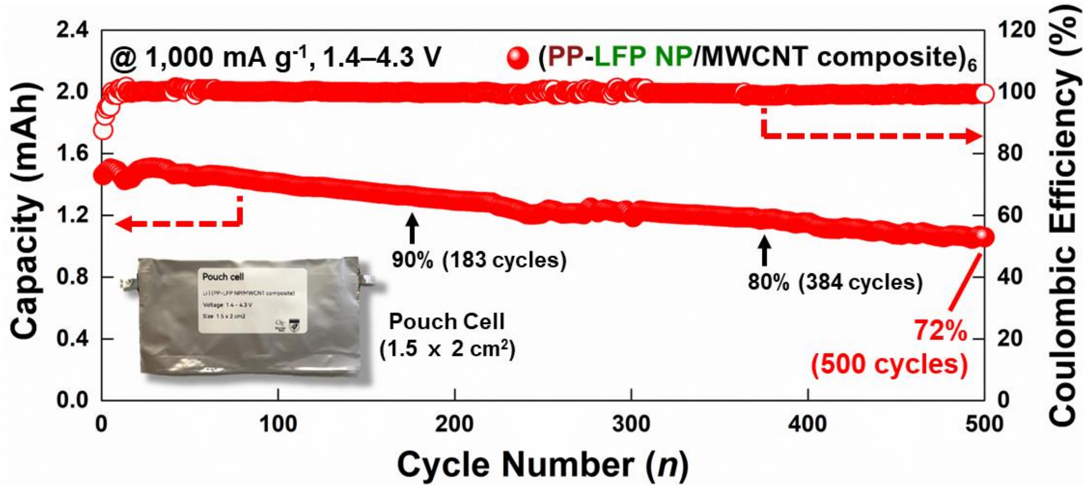

Fig. S39 Pouch cell test. Capacity retention of the (PP-LFP NP/MWCNT composite)$_6$-coated textile electrode (1.5 × 2 cm^2 configuration)-based pouch cells performed at a current density of 1,000 mA g^{-1} within a potential window of 1.4–4.3 V (*vs*. Li/Li$^+$).

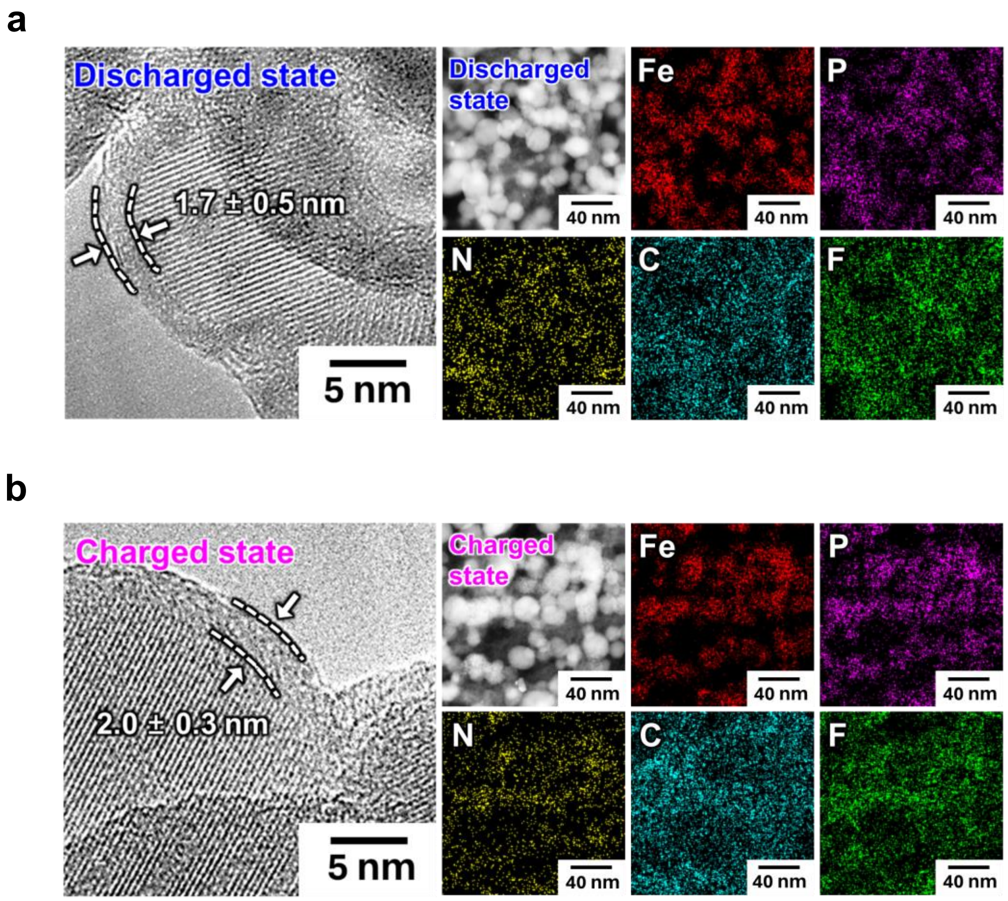

Fig. S40 CEI layer characterization. HADDF-STEM images and corresponding EDS mapping images of the CEI layer of the (PP-LFP NP/MWCNT composite) multilayers at a) the discharged (1.4 V) and b) charged (4.3 V) states after 3rd cycle at a scan rate of 0.05 mV s^{-1} within a potential window of 1.4–4.3 V (*vs*. Li/Li^+).

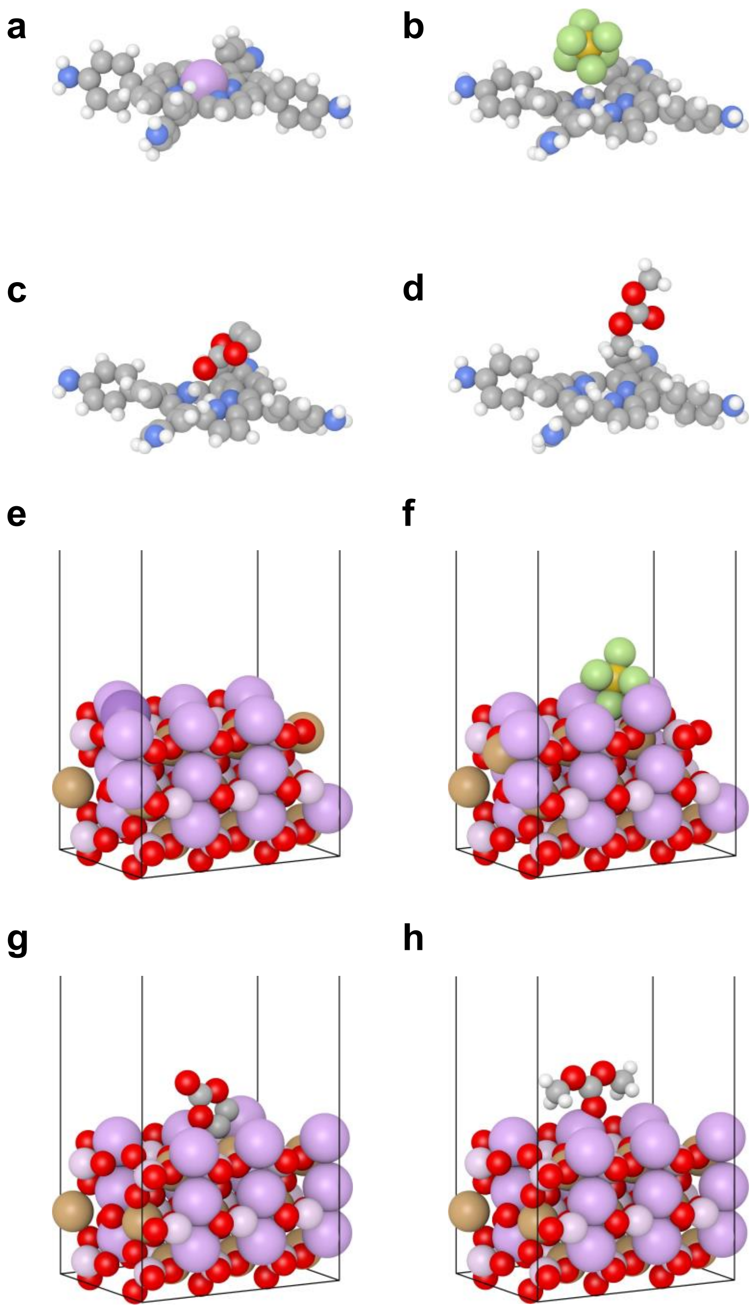

Fig. S41 DFT-optimized adsorption configurations of representative electrolyte-related species on NH_2-PP and the LFP (010) surface. a) Li^+/NH_2-PP, b) PF_6^-/NH_2-PP, c) EC/NH_2-PP, d) DMC/NH_2-PP, e) Li^+/LFP (010), f) PF_6^-/LFP (010), g) EC/LFP (010), and h) DMC/LFP (010). The adsorption configurations indicate species-dependent interfacial affinity within the PP-LFP system, with carbonate solvent molecules preferentially interacting with the LFP (010) surface and Li^+ interacting more favorably with NH_2-PP. (Atom colors: C, black; N, blue; H, white; O, red; Fe, brown; Li in LFP, dark purple; adsorbed Li^+, deep purple; P in LFP, light purple; P in PF_6^-, orange; F, green)

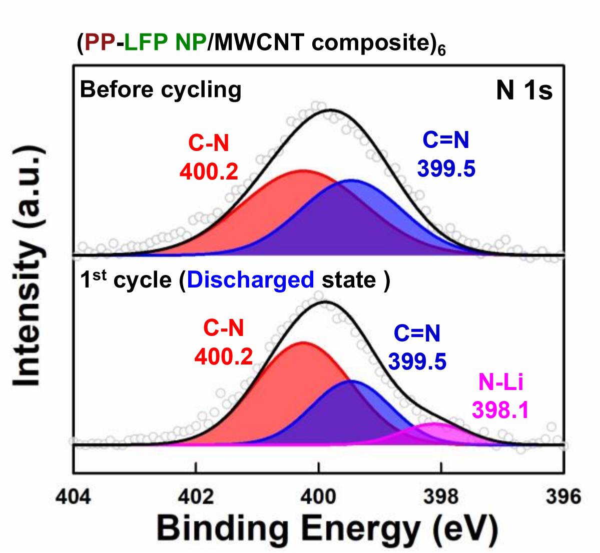

Fig. S42 XPS analysis. Deconvoluted N 1s XPS spectra of the (PP-LFP NP/MWCNT composite)$_6$-coated textile electrode before cycling and at the discharged state (1.4 V) after the 1st cycle performed at a current density of 20 mA g^{-1} within a potential window of 1.4–4.3 V (*vs*. Li/Li$^+$).

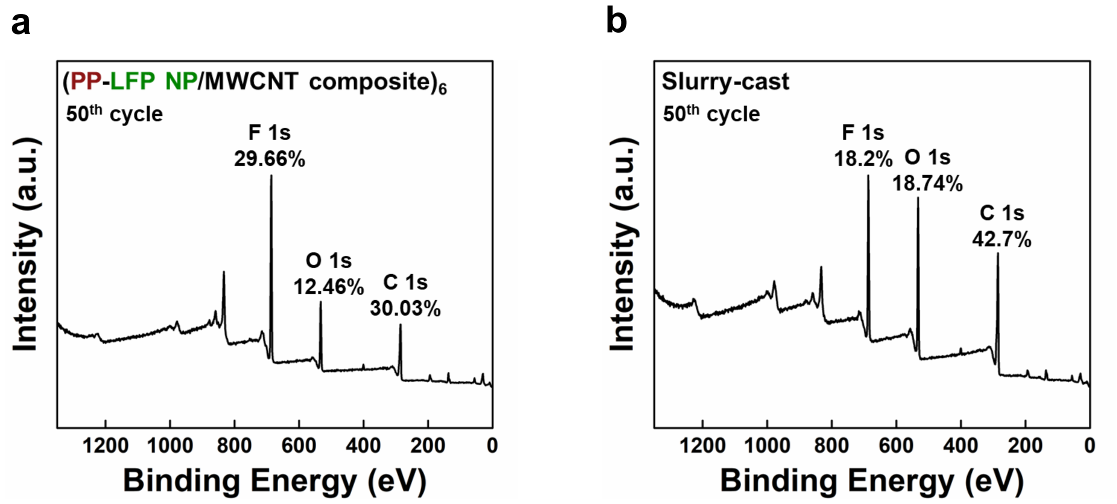

Fig. S43 XPS spectra of CEI layer. Wide-scan XPS spectra of a) the (PP-LFP NP/MWCNT composite)$_6$-coated textile electrode and b) the slurry-cast electrode after the 50th cycle performed at a current density of 200 mA g^{-1}.

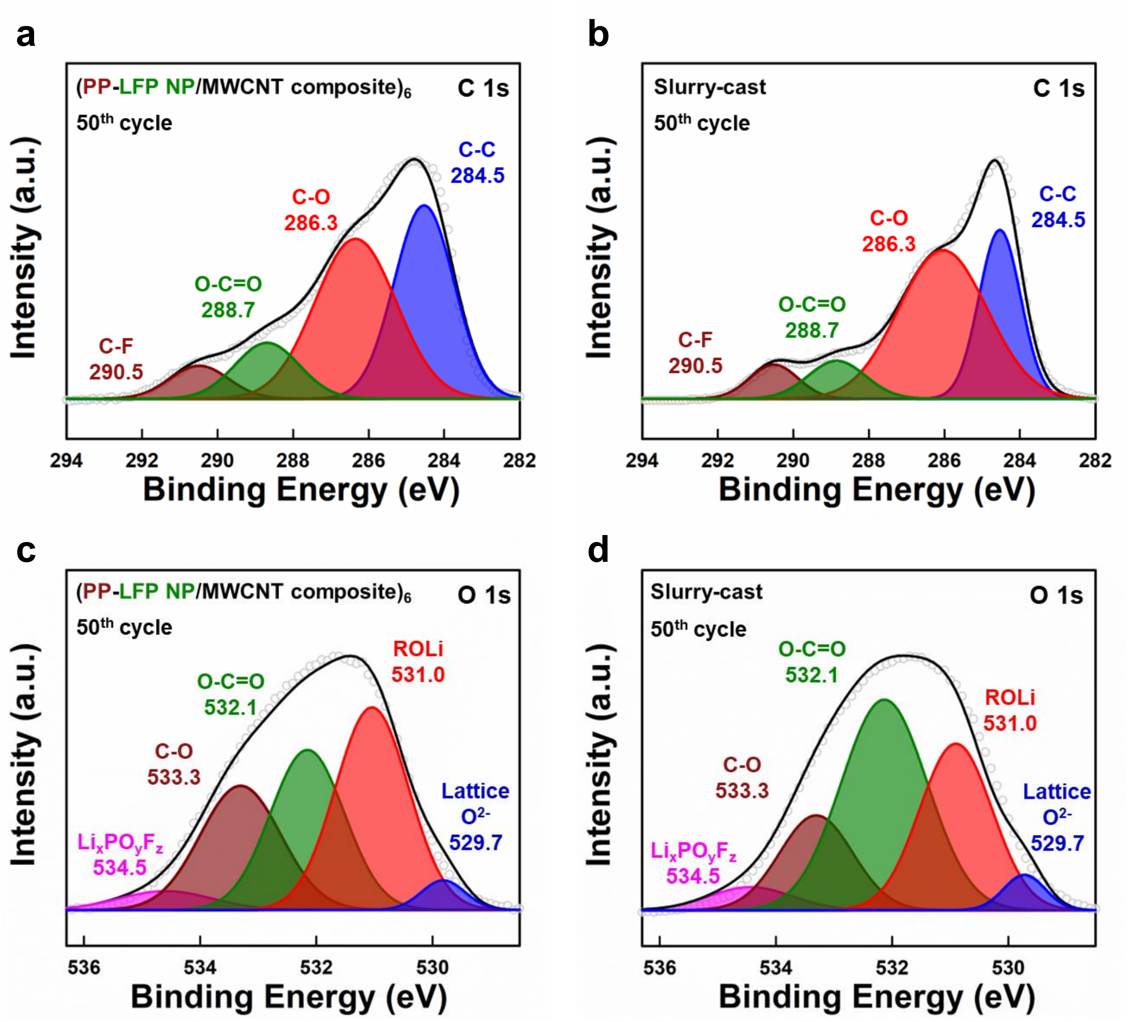

Fig. S44 XPS spectra of CEI layer. Deconvoluted C 1s XPS spectra of a) the (PP-LFP NP/MWCNT composite)$_6$-coated textile electrode and b) the slurry-cast electrode, recorded after the 50th cycle performed at a current density of 200 mA g^{-1}. Deconvoluted O 1s XPS spectra of c) the (PP-LFP NP/MWCNT composite)$_6$-coated textile electrode and d) the slurry-cast electrode, recorded after the 50th cycle performed at a current density of 200 mA g^{-1}.

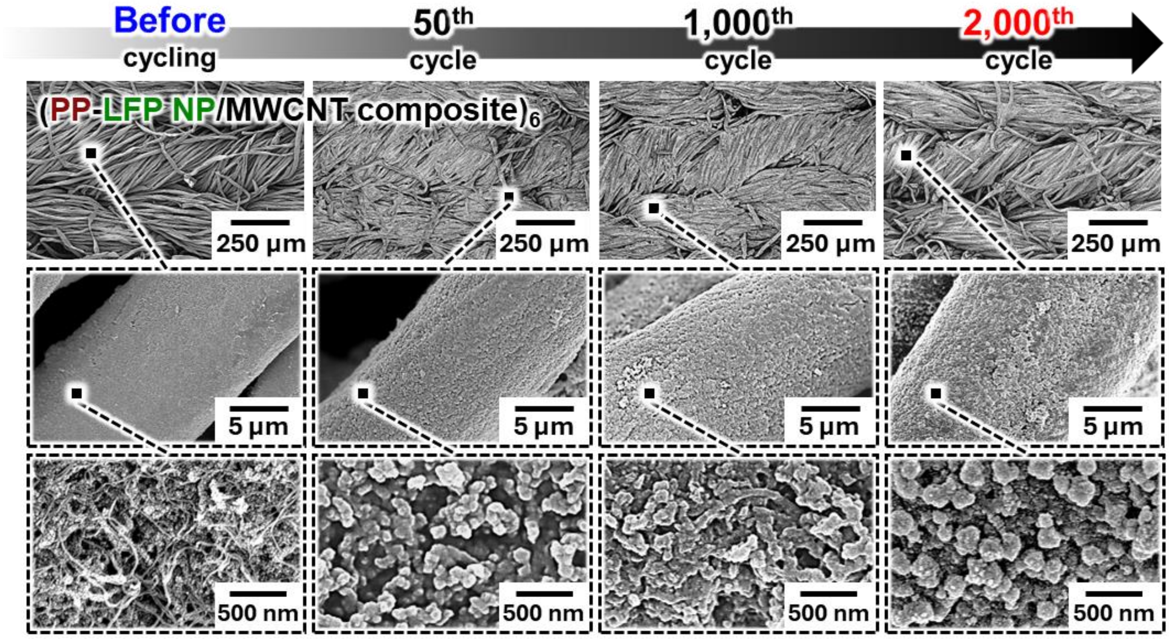

Fig. S45 Morphological characteristics after cycling. Planar FE-SEM images of (PP-LFP NP/MWCNT composite)$_6$-coated textile electrodes before cycling, after the 50th, the 1,000th and the 2,000th cycles performed at a current density of 200 mA g^{-1}.

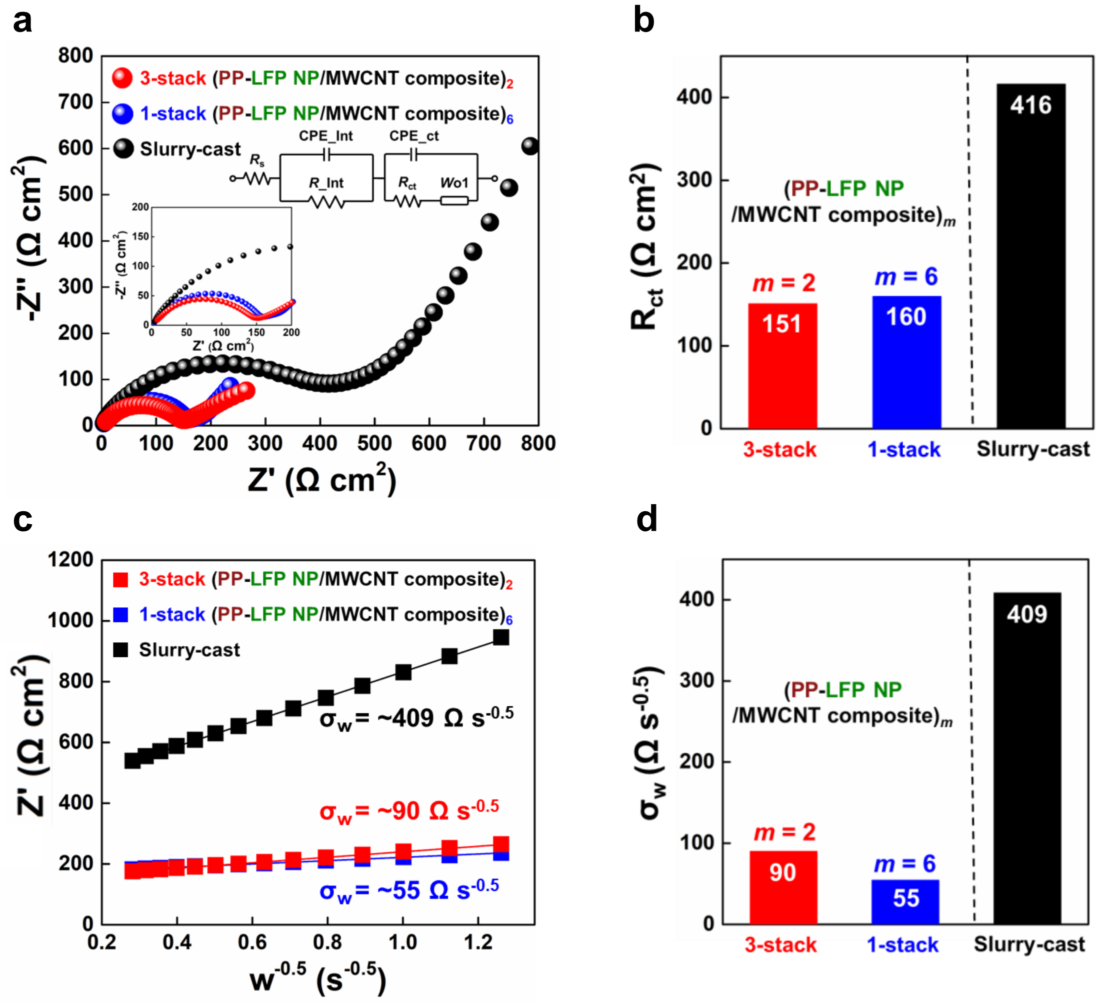

Fig. S46 Electrochemical impedance spectroscopy (EIS). a) Nyquist plots, b) charge transfer resistance (R_{ct}), and c, d) warburg impedance coefficient (σ_w) values of the 3-stack cell assembled using the (PP-LFP NP/MWCNT composite)$_2$-coated textile electrodes, the 1-stack (PP-LFP NP/MWCNT composite)$_6$-coated textile electrode and the slurry-cast electrode with the same loading mass (i.e., 3 mg cm^{-2}).

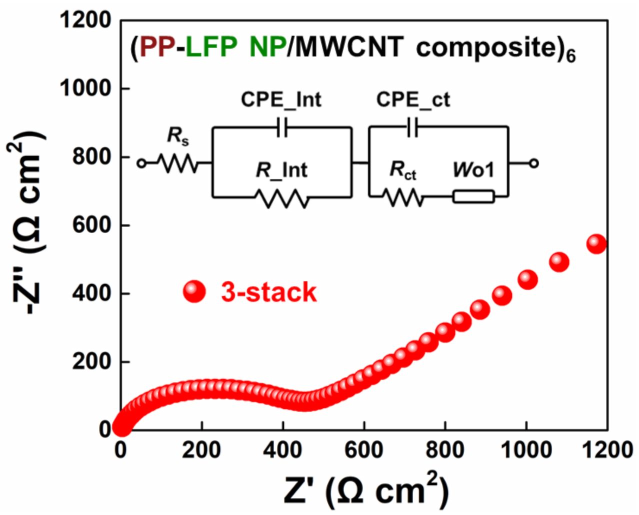

Fig. S47 Nyquist plot. Nyquist plot of 3-stack (PP-LFP NP/MWCNT composite)$_6$-coated textile electrode (i.e. total loading mass ~9.2 mg cm^{-2}).

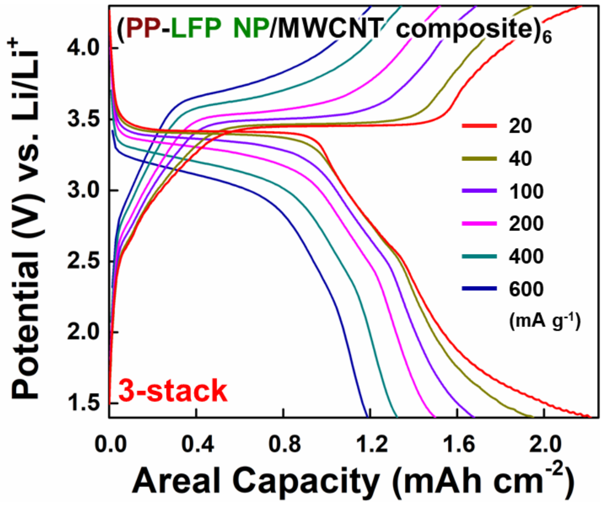

Fig. S48 GCD profiles. GCD profiles of the 3-stack (PP-LFP NP/MWCNT composite)$_6$-coated textile electrode at various current densities ranging from 20 to 600 mA g^{-1}.

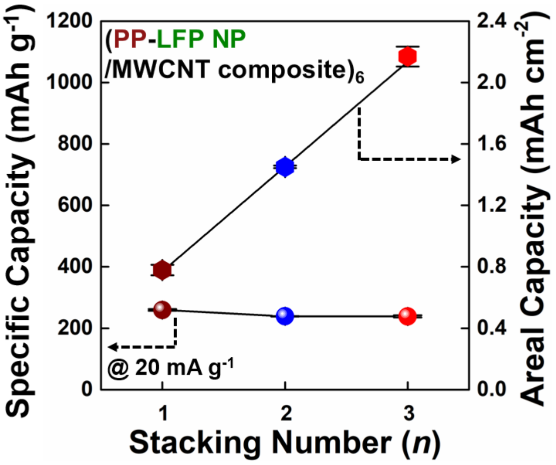

Fig. S49 Specific and areal capacities. Specific and areal capacities of the (PP-LFP/MWCNT composite)$_6$ electrode as a function of electrode stack number (*n*).

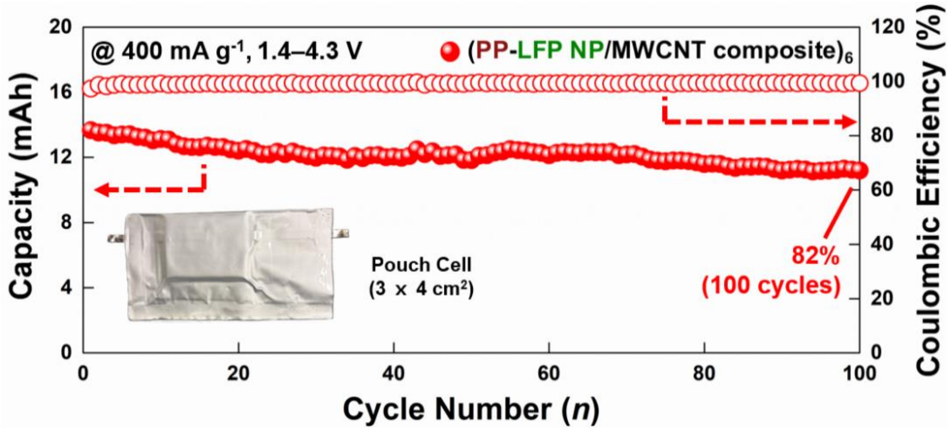

Fig. S50 Pouch cell test. Capacity retention of the 3-stack (PP-LFP NP/MWCNT composite)$_6$-coated textile electrode (3 × 4 cm^2 configuration)-based pouch cells performed at a current density of 400 mA g^{-1} within a potential window of 1.4–4.3 V (*vs*. Li/Li$^+$).

Table S1 Calculated energy densities and power densities of the (PP-LFP NP/MWCNT composite)$_6$-coated textile electrode performed at various current densities.

Current density (mA g^{-1})	Average voltage (V)	Specific capacity (mAh g^{-1})	Energy density (Wh kg^{-1})	Power density (W kg^{-1})
20	2.67	257.12	686.51	53
40	2.86	216.61	619.50	114
100	2.94	197.17	579.68	294
200	2.97	183.39	544.67	594
600	2.93	166.19	486.94	1,758
1,000	2.85	157.15	447.88	2,850
2,000	2.75	140.50	386.38	5,500

Experimental energy density: $C \times V_{avg}$

(V_{avg}: the average discharge voltage, C: the specific capacity)

The average discharge voltage (V_{avg}):

$$V_{avg} = \frac{\sum V_i \Delta Q_i}{\sum \Delta Q_i} \quad \text{(S5)}$$

(V_i and ΔQ_i: the discharge voltage and the corresponding incremental capacity at the ith data point, respectively.)

All values were calculated based on the total material loading mass.

Table S2 Performance comparison of previously reported cathodes based on layered Ni-rich oxides.

Cathode active material	Design	Mass loading	Operation condition	Specific capacity	Cycling stability	Reference
LFP	High energy ligand-stabilized LFP nanoparticle	3.0 mg cm^{-2}	1.4-4.3 V 25 °C	260 mAh g^{-1} @ ~0.1 C	Initial 200 mAh g^{-1} @ 1 C, 2,000 cycles (93%) Initial 144 mAh g^{-1} @ 10 C, 1,300 cycles (80%)	Our work
NMC	Strain-retardant coherent perovskite phase stabilized $LiNi_{0.8}Co_{0.1}Mn_{0.1}O_2$	5 mg cm^{-2}	3.0-4.3 V 25 °C	210 mAh g^{-1} @ 0.1 C	Initial: ~185 mAh g^{-1} @ 1 C, 200 cycles (94.7%)	[S24]
NMC	Dual modification: lithium sulfate coating & sulfur doping	-	3.0-4.3 V	207 mAh g^{-1} @ 0.1 C	Initial 186.9 mAh g^{-1} @ 1 C, 200 cycles (88.3%)	[S25]
NMC	Sb_2Se_3-modified NMC90	5 mg cm^{-2}	3.0-4.3 V 30 °C	~215 mAh g^{-1} @ 0.1 C	Initial: ~180 mAh g^{-1} @ 1 C, 100 cycles (96.6%) Initial: ~170 mAh g^{-1} @ 5 C, 500 cycles (80.2%)	[S26]
NMC	LiF/Li_3BO_3-modified NMC	~3.6 mg cm^{-2}	2.8-4.5 V 25 °C	~235 mAh g^{-1} @ 0.1 C	Initial: 205.4 mAh g^{-1} @ 1 C, 500 cycles (70.5%) Initial: 184.2 mAh g^{-1} @ 5 C, 300 cycles (79.2%)	[S27]
Ni	Graphene-coated LNO ($LiNiO_2$)	~5 mg cm^{-2}	2.8-4.3 V 30 °C	~230 mAh g^{-1} @ 0.1 C	Initial: ~175 mAh g^{-1} @ 1 C, 100 cycles (81.1%)	[S28]
NM	Mo–NM90 ($Li(Ni_{0.89}Mn_{0.1}Mo_{0.01})O_2$)	5 mg cm^{-2}	2.7-4.3 V 30 °C	234 mAh g^{-1} @ 0.1 C	Initial: ~217 mAh g^{-1} @ 0.5 C, 100 cycles (92.8%)	[S29]
NM	Mn-substituted Co-free cathode	5.2 mg cm^{-2}	2.8-4.8 V	~210 mAh g^{-1} @ 0.1 C	Initial: ~202 mAh g^{-1} @ 0.3 C, 100 cycles (92.1%)	[S30]
NC	$LiNi_{0.94}Co_{0.05}Te_{0.01}O_2$	~3 mg cm^{-2}	2.7-4.6 V 30 °C	239 mAh g^{-1} @ 0.1 C	Initial: ~205 mAh g^{-1} @ 0.5 C, 200 cycles (94.5%)	[S31]
NCA	W-Doped $Li[Ni_{0.95}Co_{0.04}Al_{0.01}]O_2$	~4 mg cm^{-2}	2.7-4.3 V 30 °C	242 mAh g^{-1} @ 0.1 C	Initial: ~230 mAh g^{-1} @ 0.5 C, 100 cycles (90.3%)	[S32]

Table S3 Performance comparison of previously reported cathodes based on LFP.

Cathode active material	Design	Specific capacity	Cycling stability	Reference
LFP	High energy ligand-stabilized LFP NP	260 mAh g^{-1} @ ~0.1 C	Initial 200 mAh g^{-1} @ 1 C, 2,000 cycles (93%)<br>Initial 144 mAh g^{-1} @ 10 C, 1,300 cycles (80%)	Our work
LFP	Nanoconfined electrolytes<br>: Nanoconfined channel of graphene oxide laminar membranes (GOLMS)	~165 mAh g^{-1} @ 0.1C	Initial: ~120 mAh g^{-1} @ 2 C, 100 cycles (80%)	[S33]
LFP	Ni80-30LMFP ($LiMn_{0.5}Fe_{0.5}PO_4$)<br>(Ni content: 80%, blending ratio: 30%)	187 mAh g^{-1} @ 0.1 C	Initial: 180 mAh g^{-1} @ 0.3 C, ~100 cycles (93%)	[S34]
LFP	$Li_4Fe_{0.5}Mn_{0.5}V(PO_4)_3$	220 mAh g^{-1} @ 0.1 C	Initial: ~169.4 mAh g^{-1} @ 1 C, 500 cycles (83%)	[S35]
LFP	$Zn(OAc)_2$·DEA-based LFP<br>(Zinc acetate-diethanolamine complex)	169.9 mAh g^{-1} @ 0.2 C	Initial: ~126 mAh g^{-1} @ 5 C, 1,500 cycles (64%)	[S36]
LFP	N-doped graphene based LFP	170 mAh g^{-1} @ 0.2C	Initial: ~168 mAh g^{-1} @ 1 C, 200 cycles (87.2%)<br>Initial: ~133.1 mAh g^{-1} @ 10 C, 50 cycles (92.3%)	[S37]
LFP	LFP/PMMA-g-NR binder<br>(PMMA-*g*-NR: poly(methyl methacrylate)-grafted -natural rubber)	~170 mAh g^{-1} @ 0.1 C	Initial: 104 mAh g^{-1} @ 5 C, 1200 cycles (64%)<br>Initial: 78 mAh g^{-1} @ 10 C, 1200 cycles (64%)	[S38]
LFP	MWCNT incipient coating method	157.5 mAh g^{-1} @ 0.1 C	Initial: 146 mAh g^{-1} @ 1 C, 100 cycles (98.96%)	[S39]
LFP	Encapsulated O, F-codoped carbon matrix	169.9 mAh g^{-1} @ 0.1 C	Initial: ~165 mAh g^{-1}@ 1 C, 500 cycles (97.5%)	[S40]

Table S4. Performance comparison with previously reported LFP cathodes based on total electrode mass.

Cathode active material	Design	Electrode weight (including CC)	Electrode-level specific capacity	Reference
LFP	High energy ligand-stabilized LFP NP /conductive textile current collector	25.46 mg cm^{-2}	30.7 mAh g^{-1} @ ~0.1 C	Our work
LFP	LFP/Al	21.8 mg cm^{-2}	116.5 mAh g^{-1} @ 0.5 C	[S41]
LFP	LFP/Al	9.37 mg cm^{-2}	70.6 mAh g^{-1} @ 0.1 C	[S42]
LFP	LFP/Al	6.5 mg cm^{-2}	27.9 mAh g^{-1} @ 0.05 C	[S43]

Table S5. Performance comparison with previously reported inorganic cathodes.

Cathode active material	Design	Mass loading	Operation condition	Specific capacity	Areal capacity	Reference
LFP	3-stack High energy ligand-stabilized LFP NP	9.2 mg cm^{-2}	1.4-4.3 V<br>25 °C	239 mAh g^{-1}<br>@ ~0.1 C	2.2 mAh cm^{-2}	Our work
LFP	$LiMn_{0.48}Fe_{0.48}Mg_{0.03}Nb_{0.01}PO_4$	~12 mg cm^{-2}	2.0-4.4 V<br>25 °C	~140 mAh g^{-1}<br>@ 0.1 C	1.7 mAh cm^{-2}	[S44]
LFP	LFP/CNT/$Ti_3C_2T_x$	10 mg cm^{-2}	2.0-4.2 V<br>25 °C	~155 mAh g^{-1}<br>@ 0.1 C	1.4 mAh cm^{-2}	[S45]
LFP	Zwitterionic polymer binder/LFP	10.6 mg cm^{-2}	2.5V-4.2V<br>25 °C	154.6 mAh g^{-1}<br>@ 0.1 C	1.64 mAh cm^{-2}	[S46]
LFP	PBMP/LFP electrode	8 mg cm^{-2}	2.5V-4.5V<br>25 °C	154 mAh g^{-1}<br>@ 0.1 C	1.23 mAh cm^{-2}	[S47]
NMC	Co concentration-gradient single-crystalline $LiNi_{0.5}Co_{0.2}Mn_{0.3}O_2$	~11.0 mg cm^{-2}	3.0–4.2 V<br>25 °C	158.4 mAh g^{-1}<br>@ 0.5 C	1.74 mAh cm^{-2}	[S48]
NMC	LZTO-coated $LiNi_{0.82}Co_{0.15}Mn_{0.03}O_2$	9.34 mg cm^{-2}	2.8-4.7 V<br>25 °C	~221.8 mAh g^{-1}<br>@ 0.2 C	2.07 mAh cm^{-2}	[S49]
NMC	Amorphous $LiTaO_3$-coated single-crystalline $LiNi_{0.82}Co_{0.12}Mn_{0.06}O_2$	11.0 mg cm^{-2}	2.5-4.3 V<br>25 °C	183.5 mAh g^{-1}<br>@ 0.1 C	2.02 mAh cm^{-2}	[S50]
Ni	$Li_{1.06}Ni_{0.90}W_{0.04}O_2$	8 mg cm^{-2}	2.0-4.5 V<br>25 °C	231 mAh g^{-1}<br>@ 0.1 C	1.85 mAh cm^{-2}	[S51]

Supplementary References

[S1] Y. Ko, M. Kwon, W. K. Bae, B. Lee, S. W. Lee, J. Cho. Flexible supercapacitor electrodes based on real metal-like cellulose papers. Nat. Commun. **8**(1), 536 (2017). https://doi.org/10.1038/s41467-017-00550-3

[S2] C. H. Kwon, Y. Ko, D. Shin, M. Kwon, J. Park, W. K. Bae, S. W. Lee, J. Cho. High-power hybrid biofuel cells using layer-by-layer assembled glucose oxidase-coated metallic cotton fibers. Nat. Commun. **9**(1), 4479 (2018). https://doi.org/10.1038/s41467-018-06994-5

[S3] M. Kang, D. Nam, J. Ahn, Y. J. Chung, S. W. Lee, Y.-B. Choi, C. H. Kwon, J. Cho. A mediator-free multi-ply biofuel cell using an interfacial assembly between hydrophilic enzymes and hydrophobic conductive oxide nanoparticles with pointed apexes. Adv. Mater. **35**(51), 2304986 (2023). https://doi.org/10.1002/adma.202304986

[S4] G. Decher. Fuzzy nanoassemblies: Toward layered polymeric multicomposites. Science. **277**(5330), 1232–1237 (1997). https://doi.org/doi:10.1126/science.277.5330.1232

[S5] F. Caruso, R. A. Caruso, H. Möhwald. Nanoengineering of inorganic and hybrid hollow spheres by colloidal templating. Science. **282**(5391), 1111–1114 (1998). https://doi.org/doi:10.1126/science.282.5391.1111

[S6] T. H. Wan, M. Saccoccio, C. Chen, F. Ciucci. Influence of the discretization methods on the distribution of relaxation times deconvolution: Implementing radial basis functions with drttools. Electrochim. Acta. **184**, 483–499 (2015). https://doi.org/https://doi.org/10.1016/j.electacta.2015.09.097

[S7] D. Nam, Y. Heo, S. Cheong, Y. Ko, J. Cho. Amphiphilic ligand exchange reaction-induced supercapacitor electrodes with high volumetric and scalable areal capacitances. Appl. Surf. Sci. **440**, 730–740 (2018). https://doi.org/10.1016/j.apsusc.2018.01.153

[S8] C. Choi, D. S. Ashby, D. M. Butts, R. H. DeBlock, Q. Wei, J. Lau, B. Dunn. Achieving high energy density and high power density with pseudocapacitive materials. Nat. Rev. Mater. **5**(1), 5–19 (2020). https://doi.org/10.1038/s41578-019-0142-z

[S9] M. J. Frisch, G. W. Trucks, H. B. Schlegel, G. E. Scuseria, M. A. Robb, J. R. Cheeseman, G. Scalmani, V. Barone, B. Mennucci, G. A. Petersson, et al. (2009). Gaussian 09. Gaussian, Inc., Wallingford, CT.

[S10] A. D. Becke. Density-functional thermochemistry. Iii. The role of exact exchange. J. Chem. Phys. **98**(7), 5648–5652 (1993). https://doi.org/10.1063/1.464913

[S11] C. Lee, W. Yang, R. G. Parr. Development of the colle-salvetti correlation-energy formula into a functional of the electron density. Phys. Rev. B **37**(2), 785–789 (1988). https://doi.org/10.1103/PhysRevB.37.785

[S12] W. J. Hehre, R. Ditchfield, J. A. Pople. Self—consistent molecular orbital methods. Xii. Further extensions of gaussian—type basis sets for use in molecular orbital studies of organic molecules. J. Chem. Phys. **56**(5), 2257–2261 (1972). https://doi.org/10.1063/1.1677527

[S13] G. Kresse, J. Furthmüller. Efficiency of ab-initio total energy calculations for metals and semiconductors using a plane-wave basis set. Comput. Mater. Sci. **6**(1), 15–50 (1996). https://doi.org/https://doi.org/10.1016/0927-0256(96)00008-0

[S14] G. Kresse, J. Hafner. Ab initio molecular-dynamics simulation of the liquid-metal--amorphous-semiconductor transition in germanium. Phys. Rev. B **49**(20), 14251–14269 (1994). https://doi.org/10.1103/PhysRevB.49.14251

[S15] G. Kresse, D. Joubert. From ultrasoft pseudopotentials to the projector augmented-wave method. Phys. Rev. B **59**(3), 1758–1775 (1999). https://doi.org/10.1103/PhysRevB.59.1758

[S16] J. P. Perdew, K. Burke, M. Ernzerhof. Generalized gradient approximation made simple. Phys. Rev. Lett. **77**(18), 3865–3868 (1996). https://doi.org/10.1103/PhysRevLett.77.3865

[S17] P. E. Blöchl. Projector augmented-wave method. Phys. Rev. B **50**(24), 17953–17979 (1994). https://doi.org/10.1103/PhysRevB.50.17953

[S18] H. J. Monkhorst, J. D. Pack. Special points for brillouin-zone integrations. Phys. Rev. B **13**(12), 5188–5192 (1976). https://doi.org/10.1103/PhysRevB.13.5188

[S19] K. Mathew, R. Sundararaman, K. Letchworth-Weaver, T. A. Arias, R. G. Hennig. Implicit solvation model for density-functional study of nanocrystal surfaces and reaction pathways. J. Chem. Phys. **140**(8), (2014). https://doi.org/10.1063/1.4865107

[S20] G. Sun, J. Gao, H. Li, L. Chen. Oxidized kinetic normal distribution models for sophisticated electrochemical windows. J. Phys. Chem. C **127**(20), 9554–9561 (2023). https://doi.org/10.1021/acs.jpcc.3c01873

[S21] D. S. Hall, J. Self, J. R. Dahn. Dielectric constants for quantum chemistry and li-ion batteries: Solvent blends of ethylene carbonate and ethyl methyl carbonate. J. Phys. Chem. C **119**(39), 22322–22330 (2015). https://doi.org/10.1021/acs.jpcc.5b06022

[S22] L. Wang, F. Zhou, Y. S. Meng, G. Ceder. First-principles study of surface properties of $\mathrm{li}\mathrm{fe}\mathrm{p}{\mathrm{o}}_{4}$: Surface energy, structure, wulff shape, and surface redox potential. Phys. Rev. B. **76**(16), 165435 (2007). https://doi.org/10.1103/PhysRevB.76.165435

[S23] Q. Xu, Z. Liu, Y. Jin, X. Yang, T. Sun, T. Zheng, N. Li, Y. Wang, T. Li, K. Wang, J. Jiang. A bipolar-type covalent organic framework on carbon nanotubes with enhanced density of redox-active sites for high-performance lithium-ion batteries. Energy Environ. Sci. **17**(15), 5451–5460 (2024). https://doi.org/10.1039/D4EE00520A

[S24] L. Wang, T. Liu, T. Wu, J. Lu. Strain-retardant coherent perovskite phase stabilized ni-rich cathode. Nature **611**(7934), 61–67 (2022). https://doi.org/10.1038/s41586-022-05238-3

[S25] X. Zhang, T. Wu, J. Jian, S. Lin, D. Sun, G. Fu, Y. Xu, Z. Liu, S. Li, H. Huo, Y. Ma, G. Yin, P. Zuo, X. Cheng, C. Du. Dual modification strategy for enhanced cycling and rate performance of ni-rich cathode materials in lithium-ion batteries. Small **20**(45), 2404488 (2024). https://doi.org/10.1002/smll.202404488

[S26] Y. Zou, Y. Tang, Q. Zheng, H. Zhang, Y. Yan, J. Xue, S. Zhou, J. Xu, W. Yin, H.-G. Liao, Y. Qiao, J. Bao, S.-G. Sun. Enabling the strengthened structural and interfacial stability of high-nickel lini0.9co0.05mn0.05o2 cathode by a coating-doping-microstructure regulation three-in-one strategy. Adv. Funct. Mater. **34**(41), 2406068 (2024). https://doi.org/10.1002/adfm.202406068

[S27] G. Yang, B. Liu, F. Lai, K. Xue, X. Zhang, H. Wang, M. Xie, C. Wang. Low-temperature synthesis of amorphous lif/li_3bo_3 interfaces with f, b co-doped subsurface for long-cycling and high-rate ni-rich cathodes. Nano Energy **140**, 111009 (2025). https://doi.org/10.1016/j.nanoen.2025.111009

[S28] K.-Y. Park, Y. Zhu, C. G. Torres-Castanedo, H. J. Jung, N. S. Luu, O. Kahvecioglu, Y. Yoo, J.-W. T. Seo, J. R. Downing, H.-D. Lim, M. J. Bedzyk, C. Wolverton, M. C. Hersam. Elucidating and mitigating high-voltage degradation cascades in cobalt-free linio2 lithium-ion battery cathodes. Adv. Mater. **34**(3), 2106402 (2022). https://doi.org/10.1002/adma.202106402

[S29] G.-T. Park, B. Namkoong, S.-B. Kim, J. Liu, C. S. Yoon, Y.-K. Sun. Introducing high-valence elements into cobalt-free layered cathodes for practical lithium-ion batteries. Nat. Energy **7**(10), 946–954 (2022). https://doi.org/10.1038/s41560-022-01106-6

[S30] T. Liu, L. Yu, J. Liu, J. Lu, X. Bi, A. Dai, M. Li, M. Li, Z. Hu, L. Ma, D. Luo, J. Zheng, T. Wu, Y. Ren, J. Wen, F. Pan, K. Amine. Understanding co roles towards developing co-free ni-rich cathodes for rechargeable batteries. Nat. Energy **6**(3), 277–286 (2021). https://doi.org/10.1038/s41560-021-00776-y

[S31] T. Yang, K. Zhang, Y. Zuo, J. Song, Y. Yang, C. Gao, T. Chen, H. Wang, W. Xiao, Z. Jiang, D. Xia. Ultrahigh-nickel layered cathode with cycling stability for sustainable lithium-ion batteries. Nat. Sustain. **7**(9), 1204–1214 (2024). https://doi.org/10.1038/s41893-024-01402-x

[S32] U.-H. Kim, N.-Y. Park, G.-T. Park, H. Kim, C. S. Yoon, Y.-K. Sun. High-energy w-

doped li[ni0.95co0.04al0.01]o2 cathodes for next-generation electric vehicles. Energy Storage Mater. **33**, 399–407 (2020). https://doi.org/10.1016/j.ensm.2020.08.013

[S33] Y. Yang, Z. Li, Z. Yang, Q. Zhang, Q. Chen, Y. Jiao, Z. Wang, X. Zhang, P. Zhai, Z. Sun, Y. Xiang, Y. Gong. Ultrafast lithium-ion transport engineered by nanoconfinement effect. Adv. Mater. **37**(8), 2416266 (2025). https://doi.org/10.1002/adma.202416266

[S34] S. Lee, K. Scanlan, S. Reed, A. Manthiram. Cost-effective layered oxide – olivine blend cathodes for high-rate pulse power lithium-ion batteries. Adv. Energy Mater. **15**(5), 2403002 (2025). https://doi.org/10.1002/aenm.202403002

[S35] Z. Sun, H. Zhang, S. Wang, Y. Niu, K. Zhang, Y. Xu. A novel ternary li-rich li4fe0.5mn0.5v(po4)3 cathode for ultra-high-capacity li-ion batteries with potential far beyond lfp via multi-electron reaction. Small **21**(46), e04821 (2025). https://doi.org/10.1002/smll.202504821

[S36] Z. Ma, Z. Zuo, L. Li, Y. Li. Unleash the capacity potential of lifepo4 through rocking-chair coordination chemistry. Adv. Funct. Mater. **32**(8), 2108692 (2022). https://doi.org/10.1002/adfm.202108692

[S37] Z. Zheng, F. Bei, L. Zhou, W. Xia, J. Sun, H. Qian. Efficient structural regulation platform for the controlled synthesis of lifepo4 cathodes with shorter li-ion diffusion paths. Langmuir **40**(4), 2396–2404 (2024). https://doi.org/10.1021/acs.langmuir.3c03801

[S38] K. Xia, Z. Yao, P. Zhao, Z. Liu, S. Zhao, Z. Peng, P. Zhang. Enabling 10c-rate fast charging in lifepo4 based lithium-ion battery by balancing the electronic and ionic conductivity paths in cathode. Chem. Eng. J. **506**, 160290 (2025). https://doi.org/10.1016/j.cej.2025.160290

[S39] H. An, K. Park. Lfp via nanoscale surface reforming with a tiny minimal amount of conductivity-enhancing material. Langmuir **41**(3), 1821–1829 (2025). https://doi.org/10.1021/acs.langmuir.4c04285

[S40] J. Lin, Y.-H. Sun, X. Lin. Metal-organic framework-derived lifepo4 cathode encapsulated in o,f-codoped carbon matrix towards superior lithium storage. Nano Energy **91**(106655 (2022). https://doi.org/10.1016/j.nanoen.2021.106655

[S41] J. Shang, W. Yu, L. Wang, C. Xie, H. Xu, W. Wang, Q. Huang, Z. Zheng. Metallic glass-fiber fabrics: A new type of flexible, super-lightweight, and 3d current collector for lithium batteries. Adv. Mater. **35**(26), 2211748 (2023). https://doi.org/10.1002/adma.202211748

[S42] C. Li, Z. Yang, X. Luo. Cellulose composite membranes induced by multiple hydrogen bonds as lightweight current collectors for high-performance batteries.

Commun Mater. **6**(1), 79 (2025). https://doi.org/10.1038/s43246-025-00802-6

[S43] E. Mados, I. Atar, Y. Gratz, M. Israeli, O. Kondrova, V. Fourman, D. Sherman, D. Golodnitsky, A. Sitt. Polymer-based lfp cathode/current collector microfiber-meshes with bi- and interlayered architectures for li-ion battery. J. Power Sources **603**, 234397 (2024). https://doi.org/10.1016/j.jpowsour.2024.234397

[S44] P. Vanaphuti, A. Manthiram. Enhancing the mn redox kinetics of limn0.5fe0.5po4 cathodes through a synergistic co-doping with niobium and magnesium for lithium-ion batteries. Small **20**(47), 2404878 (2024). https://doi.org/https://doi.org/10.1002/smll.202404878

[S45] S. Checko, Z. Ju, B. Zhang, T. Zheng, E. S. Takeuchi, A. C. Marschilok, K. J. Takeuchi, G. Yu. Fast-charging, binder-free lithium battery cathodes enabled via multidimensional conductive networks. Nano Lett. **24**(5), 1695–1702 (2024). https://doi.org/10.1021/acs.nanolett.3c04437

[S46] M. Yang, Z. Rong, X. Li, B. Yuan, W. Zhang. Zwitterionic polymer as binder for lifepo4 cathodes in lithium-ion batteries. Chem. Eng. J. **505**, 159332 (2025). https://doi.org/10.1016/j.cej.2025.159332

[S47] D. Zeng, C. Zhang, H. Chen, A. Zeng, J. Xu, Z. Shi, J. Xia, P. Chen, Z. Wang, K. Guo. Multifunctional copolymer dispersants in high solid content cathode slurries: From viscosity reduction to interfacial stabilization and improved lithium transport. Adv. Funct. Mater. **35**(47), 2507831 (2025). https://doi.org/10.1002/adfm.202507831

[S48] Y. Liang, X. Zhu, X. Fan, D. Li, F. Xu, H. Yu, L.-Z. Fan. Surface-enriched co engineering promoting electronic conductivity for single-crystalline ni-based layered oxide cathodes. Chem. Eng. J. **485**, 149575 (2024). https://doi.org/10.1016/j.cej.2024.149575

[S49] K. Wu, Z. Li, X. Chen. Designing a corrosion inhibiting layer to enhance cycling stability of 4.7 v ni-rich cathode. Adv. Funct. Mater. **34**(27), 2315327 (2024). https://doi.org/10.1002/adfm.202315327

[S50] M. J. You, J. Jung, Y. S. Byeon, J. Y. Jung, Y. Hong, M.-S. Park. Controlled crystallinity of litao3 surface layer for single-crystalline ni-rich cathodes for lithium-ion batteries and all-solid-state batteries. Chem. Eng. J. **483**(149199 (2024). https://doi.org/10.1016/j.cej.2024.149199

[S51] X. Gao, B. Li, G. Rousse, A. V. Morozov, M. Deschamps, E. Elkaïm, L. Zhang, K. Kummer, A. M. Abakumov, J.-M. Tarascon. Achieving high-voltage stability in li-rich ni-rich oxides with local w/ni(li) superstructure. Adv. Energy Mater. **15**(3), 2402793 (2025). https://doi.org/10.1002/aenm.202402793
